# Montmorillonite Clay-Promoted, Solvent-Free Cross-Aldol Condensations under Focused Microwave Irradiation

**DOI:** 10.3390/molecules19067317

**Published:** 2014-06-04

**Authors:** Damiano Rocchi, Juan F. González, J. Carlos Menéndez

**Affiliations:** Departamento de Química Orgánica y Farmacéutica, Facultad de Farmacia, Universidad Complutense, Madrid 28040, Spain; E-Mails: rocchid83@gmail.com (D.R.); juanfrangn@ucm.es (J.F.G.)

**Keywords:** heterogenous catalysis, clays as catalysts, Lewis acids, condensation reactions, green chemistry, aldehydes, enones

## Abstract

An environmentally benign, clean and general protocol was developed for the synthesis of aryl and heteroaryl *trans*-chalcones. This method involved solvent-free reaction conditions under microwave irradiation in the presence of a clay-based catalyst, and afforded the target compounds in good yields and short reaction times. Furthermore, the same conditions allowed the synthesis of symmetrical, diarylmethylene-α,β-unsaturated ketones from aromatic aldehydes and ketones.

## 1. Introduction

Chalcones (*trans*-1,3-diaryl-2-propen-1-ones) are a very important class of compounds due to their occurrence in Nature and their interesting and versatile pharmacological properties, as summarized in several reviews [1,2,3,4,5]. These properties include antineoplastic [5,6,7], antimalarial [8], antiviral (HIV) [9,10], antibacterial [4,11,12], antioxidant [12] and anti-inflammatory [4,13] activities, among others. These compounds are also flexible scaffolds for the construction of five- and six-membered rings or their subsequent elaboration into polycyclic systems [14,15]. Chalcones are traditionally accessed by cross-aldol condensations of aryl methyl ketones and aromatic aldehydes in the presence of alkali [16,17,18], a reaction that requires the use of an organic solvent and a highly polluting alkaline base and in most cases needs to be followed by purification by column chromatography, leading again to waste generation in the form of volatile organic solvents and discarded chromatographic stationary phases.

Economic and environmental concerns connected to the practice of organic synthesis have received especial attention in recent years. In this context, a particularly important area is the development of synthetic processes in the absence of solvents [19,20,21,22]. Microwave-assisted organic synthesis (MAOS) has emerged as an efficient and powerful tool in this area [23,24,25,26,27,28] and often leads to simple protocols, short processing times, increased product yields, energy savings [29] and lower costs, thereby enabling environmentally friendly processes [30]. On the other hand, methods based on the use of heterogeneous catalysts are widely used in industrial fine and pharmaceutical chemistry, and play an important role in the current bid for the development of green synthetic processes. In particular, there is much interest in the use of clays as solid acid catalysts because of their desirable properties such as environmental compatibility, non-corrosive and non-toxic nature, low cost and, furthermore, because they often allow very simple isolation procedures. To summarize, heterogeneous catalysis is crucial to chemical technology, and clays in particular are finding increasing applications as catalysts [31,32,33,34].

In this paper, we describe a method that combines the desirable features of both approaches and its application to the preparation of chalcones. The need for this research became apparent in the course of our program on the use of aryl and heteroaryl *trans*-chalcones in heterocyclic synthesis [35,36,37,38], when we noticed the shortcomings of the currently available protocols for the synthesis of the required starting materials. Thus, we present here our studies on a solvent-free protocol for their synthesis under microwave irradiation conditions in the presence of a montmorillonite clay catalyst, its application to a large number of examples in order to establish its scope and a brief study of its subsequent generalization to other types of substrates.

## 2. Results and Discussion

We started our study by examining the model cross aldol condensation reaction between acetophenone (**1a**) and benzaldehyde (**2a**) at a relatively large scale (8.6 mmol) in the presence of montmorillonite KSF (MKSF). The choice of catalyst was based on literature precedent for the thermal cross-condensation of acetophenone and benzaldehyde in the presence of clay although we were concerned about its generality, since the scope of this literature method was quite narrow [39]. Thus, we set out to optimize conditions for this model reaction by varying the following experimental parameters: temperature, time, stoichiometry, and amount of clay. In order to study the influence of temperature, an equimolecular mixture of **1a** and **2a** with 0.3 g/mol of the clay catalyst was irradiated under focused microwave for 1 hour. When the reaction mixture was heated at 100 °C or 120 °C, poor conversions were observed (Table 1, entries 1 and 2), but a further increase in the reaction temperature to 150 °C afforded chalcone **3a** in a very good 88% yield (Table 1, entry 3). The next parameter that we investigated was the amount of catalyst, and to this end we performed the reaction with an equimolecular mixture of **1a** and **2a**, for 1 hour at 150 °C, in the presence of MKSF 0.06, 0.12, 0.24 and 0.30 gram of clay per mol of **1a**. These experiments led us to select 0.24 g/mol as the optimal clay/substrate ratio (Table 1, entries 5 to 8). Under these conditions, reaction times below 1 h (entries 9 and 10) proved to be detrimental to conversion and were therefore avoided in further experiments. We also observed that an increase of the **1a**/**2a** ratio led to decreased yield (Table 1, entry 11). When the reaction was carried out in a multigram scale, an increase in the temperature was required in order to obtain chalcone **3a** in a good yield (Table 1, entry 12). Finally, in order to compare the performance of microwave irradiation *versus* conventional heating, the reaction was performed at 150 °C in an oil bath using the optimal **1a**/**2a** ratio and amount of clay. In this experiment, a very long reaction time (20 h) was needed for the reaction to achieve completion, and **3a** was isolated in a poor 16% yield, together with many unidentified by-products (Table 1, entry 13). To summarize these experiments, it can be concluded that focused microwave irradiation has a highly beneficial effect on the reaction, both in terms of yields and reaction times.

**Table 1:** Optimization of the synthesis of model chalcone **3a**. 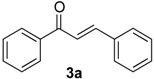

Entry	1a/2a	T (°C)	Time	KSF/substrate ratio (g/mol)	Yield (%) ^a^
1	1/1	100 °C	60 min	0.30	8
2	1/1	120 °C	60 min	0.30	23
3	1/1	150 °C	60 min	0.30	88
4	1/1	160 °C	60 min	0.30	88
5	1/1	150 °C	60 min	0.06	5
6	1/1	150 °C	60 min	0.12	81
7	1/1	150 °C	60 min	0.24	97
8	1/1	150 °C	60 min	0.30	88
9	1/1	150 °C	40 min	0.24	74
10	1/1	150 °C	20 min	0.24	50
11	2/1	150 °C	60 min	0.24	86
12	1/1	160 °C	60 min	0.24	78 ^b^
13	1/1	150 °C	20 h	0.29	16 ^c^

^a^ Isolated yields, when the reaction was performed under microwave irradiation at 0.86 mmol scale, except where noted otherwise; ^b^ Isolated yields, when the reaction was performed under microwave irradiation in a 30 mmol scale; ^c^ Isolated yield under reflux conditions.

**Scheme 1:**
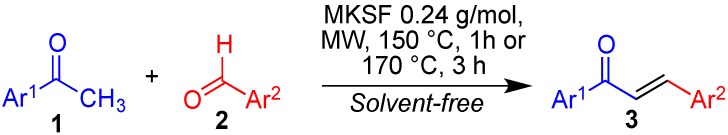
Clay-promoted synthesis of chalcones under microwave irradiation.

The substrate scope of the cross aldol condensation reaction in the presence of the MKSF clay catalyst was explored with a variety of substituted acetophenone substrates containing either electron donating or electron withdrawing groups, with the results shown in Scheme 1 and Table 2. As expected, the presence of electron-releasing substituents in the acetophenone component, which acts as the nucleophile, is slightly beneficial to yield (compare, for instance, the yields in entries 1, 10, 19, 23 and 26). The reaction was not sensitive to steric hindrance in the aldehyde, since it tolerates well the presence of *ortho* substituents (compare the yields in entries 4–5, 7–9 and 15–16). Generally speaking, the substituents at the aromatic ring of the aldehyde component did not have a significant influence in the yield, but it is remarkable that some of the best results corresponded to reactions with methoxybenzaldehyde derivatives (entries 2, 11, 15, 20), in spite of the fact that the electron-releasing nature of the methoxy substituent should lead to a lower reactivity as electrophiles.

**Table 2:** Scope and yields in the synthesis of chalcones **3**.

Entry	Ar^1^	Ar^2^		Compound	Yield (%) ^a^		
1	C_6_H_5_	C_6_H_5_		3a	97		
2	C_6_H_5_	4-MeOC_6_H_4_		3b	85		
3	C_6_H_5_	4-MeC_6_H_4_		3c	55		
4	C_6_H_5_	4-ClC_6_H_4_		3d	74		
5	C_6_H_5_	2-ClC_6_H_4_		3e	77		
6	C_6_H_5_	4-BrC_6_H_4_		3f	69		
7	C_6_H_5_	2-NO_2_C_6_H_4_		3g	51		
8	C_6_H_5_	3-NO_2_C_6_H_4_		3h	44		
9	C_6_H_5_	4-NO_2_C_6_H_4_		3i	43 (57) ^b^		
10	4-MeOC_6_H_4_	C_6_H_5_		3j	91		
11	4-MeOC_6_H_4_	4-MeOC_6_H_4_		3k	83		
12	4-MeOC_6_H_4_	4-BrC_6_H_4_		3l	73		
13	4-MeC_6_H_4_	C_6_H_5_		3m	95		
14	4-MeOC_6_H_4_	4-NO_2_C_6_H_4_		3n	55		
15	4-MeC_6_H_4_	2-MeOC_6_H_4_		3o	93		
16	4-MeC_6_H_4_	4-MeC_6_H_4_		3p	77		
17	4-MeC_6_H_4_	4-BrC_6_H_4_		3q	76		
18	4-MeC_6_H_4_	2-NO_2_C_6_H_4_		3r	66		
19	4-ClC_6_H_4_	C_6_H_5_		3s	54		
20	4-ClC_6_H_4_	3-OMeC_6_H_4_		3t	78		
21	4-ClC_6_H_4_	4-BrC_6_H_4_		3u	64		
22	4-ClC_6_H_4_	2-NO_2_C_6_H_4_		3v	48		
23	4-BrC_6_H_4_	C_6_H_5_		3w	60		
24	4-BrC_6_H_4_	4-BrC_6_H_4_		3x	86		
25	4-BrC_6_H_4_	4-NO_2_C_6_H_4_		3y	49		
26	4-NO_2_C_6_H_4_	C_6_H_5_		3z	65 ^c^		
27	4-NO_2_C_6_H_4_	4-OMeC_6_H_4_		3aa	64 ^c^		
28	4-NO_2_C_6_H_4_	4-MeC_6_H_4_		3ab	77 ^c^		
29	4-NO_2_C_6_H_4_	4-BrC_6_H_4_		3ac	68 ^c^		
30	4-NO_2_C_6_H_4_	4-NO_2_C_6_H_4_		3ad	60 ^c^		
31	C_6_H_5_	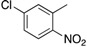		3ae	57		
32	C_6_H_5_	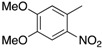		3af	63		
33	4-MeC_6_H_4_	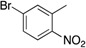		3ag	54		
34	4-MeC_6_H_4_	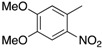		3ah	64		
35	4-BrC_6_H_4_	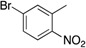		3ai	48		
36	C_6_H_5_	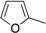		3aj	85		
37	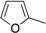	C_6_H_5_		3ak	60		
38	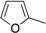	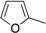		3al	97		
39	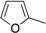	2-NO_2_C_6_H_4_		3am	45		
40	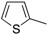	C_6_H_5_		3an	54		
41	C_6_H_5_	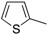		3ao	78		
42	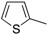	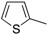		3ap	92		

^a^ General reaction conditions: A mixture of aldehyde **2** (0.86 mmol), ketone **1** (0.86 mmol) and MKSF (200 mg) in a sealed tube, was heated under microwave irradiation at 150 °C for 1 h; ^b^ Reaction conditions for the reaction proceeding in 57% yield: 170 °C, 3 h; ^c^ Reaction conditions for entries 26–30: A mixture of aldehyde **2** (0.86 mmol), ketone **1** (0.86 mmol) and MKSF (200 mg) in a sealed tube, was heated under microwave irradiation at 170 °C for 3 h.

**Scheme 2:**
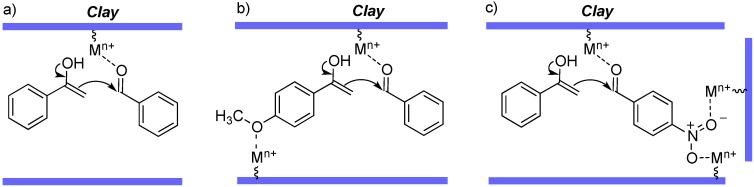
Explanation proposed for the observed substituent effects on yield.

Since the catalytic affect of the clay can be attributed to the Lewis acid activity of its metallic centers (Scheme 2a), the good reactivity of the methoxy derivatives can be explained by accepting that coordination of the oxygen atom in the OMe group with cationic centers in the clay attenuates its electron-releasing effect (Scheme 2b) [40]. Another special case was that of nitrobenzaldehyde derivatives which, again unexpectedly, gave relatively low yields (entries 7, 8, 9, 13, 18, 22, 25, 30–35). In this case, we propose that the lower reactivity is due to an increased activation energy associated to stabilizing interactions of the aldehyde with the clay by coordinating two of the cationic centers (Scheme 2c). In agreement to this explanation, we found an increase in yield from 43% to 57% for the case of compound **3i** when changing the conditions from 150 °C, 1 h to 170 °C, 3 h (enthy 9). Unfortunately, higher temperatures could not be used because they led to the decomposition of *p*-nitrobenzaldehyde. Finally, in order to demonstrate the wide application of this methodology, the reaction was carried out with selected examples of heteroaromatic aldehydes and ketones, to give good yields of compounds **3aj** to **3ap** (entries 36–42).

**Scheme 3:**
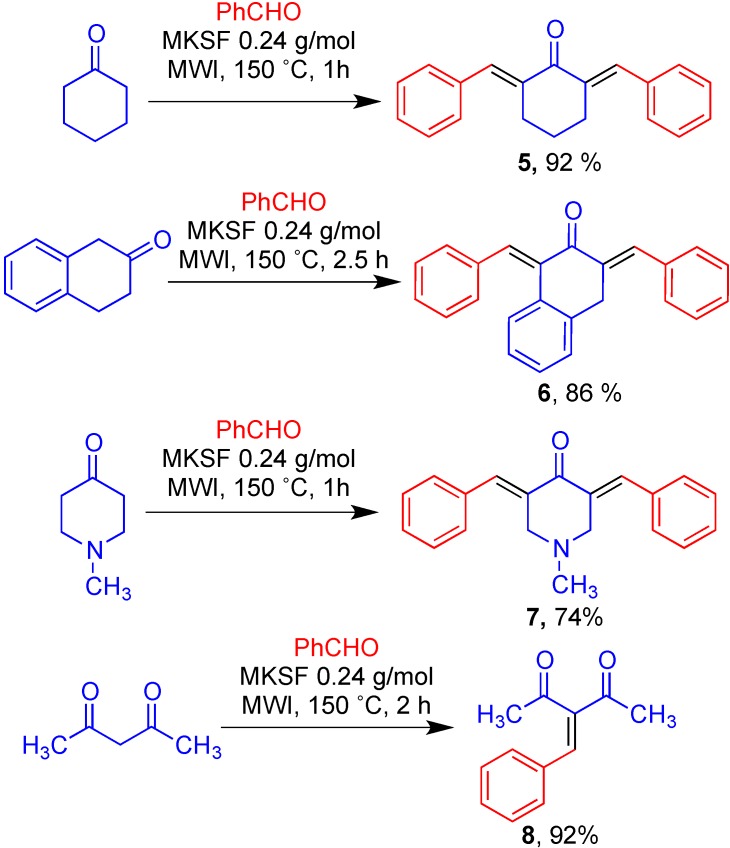
Additional examples showing the generality of the clay-promoted condensations.

In order to further demonstrate the generality of this methodology, the pseudo three-component, double aldol reactions of representative aliphatic ketones with two equivalents of benzaldehyde were briefly examined under the optimal microwave conditions previously developed, leading to compounds **5**–**7** in good to excellent yields. Also, one example of a Knoevenagel reaction was carried out, affording compound **8** [41] in an excellent 92% yield (Scheme 3).

## 3. Experimental Section

### 3.1. General Information

Melting points were measured in open capillary tubes and are uncorrected. A CEM Discover focused microwave synthesizer with a maximum microwave power level of 400 W and microwave frequency of 2,444 MHz was employed. The ^1^H-NMR and ^13^C-NMR spectra were recorded on a Bruker (Avance) 250 MHz NMR instrument maintained by the CAI de Resonancia Magnética, Universidad Complutense, using unless indicated otherwise CDCl_3_ as solvent and the residual CHCl_3_ as reference. Chemical shifts are given in parts per million (δ scale) and the coupling constants are given in Hertz. Silica gel-G plates (Merck) were used for TLC analysis. Elemental analyses were measured by the CAI de Microanálisis Elemental, Universidad Complutense, on a Leco 932 CHNS analyser. IR spectra were recorded on a Perkin Elmer Paragon 1000 FT IR instrument (neat samples on a NaCl window). Montmorillonite KSF (product number 28,153-0) was purchased from Sigma-Aldrich (Madrid, Spain) and used as received. This particular clay has 20–25 μm particle size and a surface area of 20–40 m^2^/g.

### 3.2. General Procedure for Cross Aldol Condensations

A mixture of the suitable aldehyde (1.0 mmol), acetophenone (1.0 mmol) and clay catalyst (240 mg) was warmed at 150 °C in a sealed tube under microwave irradiation, for 1 h. In the reactions involving solid starting materials, they were thoroughly mixed by grinding in a mortar before irradiation. The reaction mixture was diluted with hot ethanol (20 mL), the catalyst was filtered off, the solvent was evaporated and the residue was purified by crystallization (EtOH) for solid chalcones (compounds **3a**–**3d**, **3f**–**3m**, **3p**–**3ar**) or by column chromatography (silica gel, ethyl acetate/hexanes) for oily chalcones (compounds **3e**, **3o**), to afford the pure final products. All yields were calculated from isolated products. Characterization data for previously unknown compounds are given below. For full characterization data, see the Supporting Information.

*(E)*-*3-(5-Bromo-2-nitrophenyl)-1-(p-tolyl)-2-propen-1-one* (**3ag**). White solid (54%). M.p. 164–166 °C. IR ν_max_ (KBr): 1663, 1598, 1520 cm^−1^. ^1^H-NMR δ: 8.08 (1H, d, *J =* 15.7 Hz), 8.00–7.83 (4H, m), 7.68 (1H, dd, *J =* 9.0, 2.8 Hz), 7.32 (3H, m), 2.45 (3H, s). ^13^C-NMR δ: 189.6, 147.2, 144.5, 138.5, 134.8, 133.6, 133.3, 132.3, 129.7, 129.1, 128.6, 128.3, 126.7, 21.9. Anal. Calcd. for C_16_H_12_BrNO_3_: C, 55.51; H, 3.49; N, 4.05. Found: C, 55.28; H, 3.21 N, 3.95.

*(E)-3-(4,5-Dimethoxy-2-nitrophenyl)-1-(p-tolyl)-2-propen-1-one* (**3ah**). White solid (64%). M.p. 180–182 °C. ^1^H-NMR δ 8.20 (d, *J =* 15.7 Hz, 1H), 7.97–7.91 (m, 2H), 7.67 (s, 1H), 7.36–7.29 (m, 2H), 7.20 (d, *J =* 15.7 Hz, 1H), 7.07 (s, 1H), 4.06 (s, 3H), 4.01 (s, 3H), 2.45 (s, 3H). ^13^C-NMR δ 190.8, 153.3, 150.0, 144.0, 141.4, 141.1, 135.0, 129.5, 129.1, 126.7, 126.2, 110.2, 108.1, 56.7, 56.6, 21.8. Anal. Calcd. for C_18_H_17_NO_5_: C, 66.05; H, 5.23; N, 4.28. Found: C, 59.93; H, 5.09; N, 4.15.

*(E)-3-(5-Bromo-2-nitrophenyl)-1-(4-bromophenyl)-2-propen-1-one* (**3ai**). White solid (48%). M.p. 191–193 °C. ^1^H-NMR, DMSO-d_6_) δ: 8.49 (1H, d, *J =* 2.7 Hz), 8.16 (2H, d, *J =* 9.0 Hz), 8.05 (1H, d, *J =* 9.0 Hz), 7.98 (2H, d, *J =* 3.8 Hz), 7.92 (1H, dd, *J =* 9.0, 2.7 Hz), 7.82 (2H, d, *J =* 9.0 Hz). ^13^C-NMR (63 MHz, DMSO-d_6_) δ: ^13^C-NMR (63 MHz, DMSO) δ 188.0, 147.8, 137.7, 135.9, 133.7, 132.0, 131.8, 130.9, 127.9, 127.6, 126.9, 126.8. Anal. Calcd. for C_15_H_9_Br_2_NO_3_: C, 43.83; H, 2.21; N, 3.41. Found: C, 43.57; H, 2.39; N, 3.17.

## 4. Conclusions

In conclusion, we have developed a solvent-free, inexpensive and fast microwave-assisted method for cross aldol condensations, catalysed by the acidic clay montmorillonite KSF, with a broad scope of application. In comparison to previously reported methods, where strong acids or bases are normally required, the protocol reported here constitutes a user- and environment-friendly alternative that proceeds normally in good to excellent yields.

## Supplementary Materials

Supplementary materials can be accessed at: http://www.mdpi.com/1420-3049/19/6/7317/s1.
