# Supplementary material for: Montmorillonite Clay-Promoted, Solvent-Free Cross-Aldol Condensations under Focused Microwave Irradiation
Source: Molecules. 2014 Jun 4;19(6):7317–26. doi: 10.3390/molecules19067317 (PMC6271373; doi:10.3390/molecules19067317)
Supplement: Supplementary file 1 [file molecules-19-07317-s001.pdf]

Supporting Information

Table of Contents

1. General Procedures and Characterization Data of All Compounds **S1–S7**

2. ^{1}H and ^{13}C NMR Spectra of All Compounds **S8–S52**

3. References **S53–S55**

1. General Procedure and Characterization Data of All Compounds Listed in Table 2 and Scheme 1

1.1. General Procedure

Chalcone synthesis was performed in a sealed tube under solvent-free conditions. A mixture of the corresponding aldehyde (1.0 mmol), acetophenone (1.0 mmol) and catalyst (240 mg) was warmed at 150 ℃ under microwave irradiation, for 1 h or at 170 ℃ for 3h (compounds **3aa**–**3ad**). The reaction mixture was diluted with hot ethanol (20 mL), the catalyst was filtered off, and then the solvent was evaporated, and the residue purified by crystallization for solid chalcones or by column chromatography (silica gel, ethyl acetate/hexanes, 9/1) to afford pure chalcones. All the yields were calculated from isolated products. The spectral data of isolated compounds are listed below.

***(E)*-1,3-Diphenylpropenone 3a (table 2, entry 1)** [1]
Pale-yellow solid (97%), mp 55–56 ℃. 1H NMR (250 MHz, $CDCl_3$) δ 8.03 (dd, *J* = 8.2, 1.4 Hz, 2H), 7.82 (d, *J* = 15.7 Hz, 1H), 7.71–7.64 (m, 2H), 7.60–7.47 (m, 4H), 7.45–7.38 (m, 3H). The spectroscopic data were consistent with those of an authentic commercial sample and with those reported in the literature.

***(E)*-3-(4-Methoxyphenyl)-1-phenyl-2-propen-1-one 3b (table 2, entry 2)** [2]
Pale yellow solid (85%). ^{1}H NMR (250 MHz, $CDCl_3$) δ 8.01 (dd, *J* = 6.8, 1.6 Hz, 2H), 7.79 (d, *J* = 15.7 Hz, 1H), 7.61 (d, *J* = 8.8 Hz, 2H), 7.58–7.46 (m, 3H), 7.42 (d, *J* = 15.7 Hz, 1H), 6.94 (d, *J* = 8.8 Hz, 2H), 3.86 (s, 3H). ^{13}C NMR (63 MHz, $CDCl_3$) δ = 188.8, 163.5, 144.0, 135.1, 131.1, 130.9, 130.4, 129.0, 128.4, 121.9, 113.9, 55.6. The spectroscopic data were consistent with those reported in the literature.

***(E)*-1-Phenyl-3-(*p*-tolyl)-2-propen-1-one 3c (table 2, entry 3)** [3]
White solid (55 %). ^{1}H NMR (250 MHz, $CDCl_3$) δ 8.03 (d, *J* = 6.9 Hz, 2H), 7.81 (d, *J* = 15.7 Hz, 1H), 7.63–7.43 (m, 6H), 7.23 (d, *J* = 8.0 Hz, 2H), 2.39 (s, 3H). ^{13}C NMR (63 MHz, $CDCl_3$) δ 190.7, 145.0, 141.2, 138.4, 132.7, 132.2, 129.8, 128.7, 128.5, 121.1, 21.6. The spectroscopic data were consistent with those reported in the literature.

***(E)*-3-(4-Chlorophenyl)-1-phenyl-2-propen-1-one 3d (table 2, entry 4)** [2]
Pale yellow solid (74%), ^{1}H NMR (250 MHz, $CDCl_3$) δ 8.09–8.02 (m, 2H), 7.78 (d, *J* = 15.7 Hz, 1H), 7.67–7.46 (m, 6H), 7.46–7.37 (m, 2H). ^{13}C NMR (63 MHz, $CDCl_3$) δ 190.2, 143.3, 138.1, 136.5,

133.4, 133.0, 129.7, 129.3, 128.8, 128.6, 122.5. The spectroscopic data were consistent with those reported in the literature.

***(E)*-3-(2-Chlorophenyl)-1-phenyl-2-propen-1-one 3e (table 2, entry 5)** [4]

Pale yellow oil (77%). ^{1}H NMR (250 MHz, $CDCl_3$) δ 8.19 (d, *J* = 15.8 Hz, 1H), 8.06–7.98 (m, 2H), 7.79–7.73 (m, 1H), 7.62–7.41 (m, 6H), 7.37–7.30 (m, 2H). ^{13}C NMR (63 MHz, $CDCl_3$) δ 190.6, 140.8, 138.1, 135.6, 133.4, 133.1, 131.3, 130.5, 128.8, 128.8, 127.9, 127.2, 124.9 The spectroscopic data were consistent with those reported in the literature.

***(E)*-3-(4-Bromophenyl)-1-phenyl-2-propen-1-one 3f (table 2, entry 6)** [2]

Pale yellow solid (69%). ^{1}H NMR (250 MHz, $CDCl_3$) δ 8.02 (dd, *J* = 8.3, 1.4 Hz, 2H), 7.75 (d, *J* = 15.7 Hz, 1H), 7.62–7.57 (m, 2H), 7.56–7.45 (m, 6H). ^{13}C (63 MHz, $CDCl_3$) 190.3, 143.5, 138.1, 133.9, 133.1, 132.3, 129.9, 128.8, 128.6, 124.9, 122.7. The spectroscopic data were consistent with those reported in the literature.

***(E)*-3-(2-Nitrophenyl)-1-phenyl-2-propen-1-one 3g (table 2, entry 7)** [5]

White solid (51 %). ^{1}H NMR (250 MHz, $CDCl_3$) δ 8.16 (d, *J* = 15.7 Hz, 1H), 8.12–8.02 (m, 3H), 7.81–7.68 (m, 2H), 7.68–7.50 (m, 4H), 7.36 (d, *J* = 15.7 Hz, 1H). ^{13}C NMR (63 MHz, $CDCl_3$) δ 190.5, 148.6, 140.3, 137.5, 133.7, 133.3, 131.4, 130.5, 129.4, 128.9, 128.8, 127.4, 125.1. The spectroscopic data were consistent with those reported in the literature.

***(E)*-3-(3-Nitrophenyl)-1-phenyl-2-propen-1-one 3h (table 2, entry 8)** [6]

Pale yellow solid (44%). ^{1}H NMR (250 MHz, $CDCl_3$) δ 8.50 (t, *J* = 1.9 Hz, 1H), 8.25 (ddd, *J* = 8.2, 2.2, 1.0 Hz, 1H), 8.04 (dd, *J* = 8.3, 1.4 Hz, 2H), 7.92 (d, *J* = 7.7 Hz, 1H), 7.83 (d, *J* = 15.8 Hz, 1H), 7.71–7.46 (m, 4H). ^{13}C NMR (63 MHz, $CDCl_3$) δ = 189.8, 148.8, 141.7, 137.6, 136.7, 134.5, 133.4, 130.2, 128.9, 128.7, 124.8, 124.7, 122.4. The spectroscopic data were consistent with those reported in the literature.

***(E)*-3-(4-Nitrophenyl)-1-phenyl-2-propen-1-one 3i (table 2, entry 9)** [7]

Pale yellow solid (43%). ^{1}H NMR (250 MHz, $CDCl_3$) δ 8.28 (d, *J* = 8.9 Hz, 2H), 8.04 (dd, *J* = 8.3, 1.3 Hz, 2H), 7.88–7.75 (m, 3H), 7.70–7.59 (m, 2H), 7.57–7.48 (m, 2H); ^{13}C NMR (63 MHz, $CDCl_3$) δ 189.7, 141.6, 141.1, 137.6, 133.5, 129.0, 128.9, 128.7, 125.8, 124.3. The spectroscopic data were consistent with those reported in the literature.

***(E)*-1-(4-Methoxyphenyl)-3-phenyl-2-propen-1-one 3j (table 2, entry 10)** [8]

Pale yellow solid (91%). ^{1}H NMR (250 MHz, $CDCl_3$) δ 8.05 (d, *J* = 8.9 Hz, 2H), 7.81 (d, *J* = 15.7 Hz, 1H), 7.68–7.62 (m, 2H), 7.55 (d, *J* = 15.7 Hz, 1H), 7.45–7.34 (m, 3H), 6.98 (d, *J* = 8.9 Hz, 2H), 3.87 (s, 3H). ^{13}C NMR (63 MHz, $CDCl_3$) δ 188.7, 163.5, 144.0, 135.2, 131.2, 130.9, 130.4, 129.0, 128.4, 121.9, 113.9, 55.6. The spectroscopic data were consistent with those reported in the literature.

***(E)*-1,3-bis(4-Methoxyphenyl)-2-propen-1-one 3k (table 2, entry 11)** [9]

Pale yellow solid (83%). ^{1}H NMR (250 MHz, $CDCl_3$) δ 8.03 (d, *J* = 8.9 Hz, 2H), 7.78 (d, *J* = 15.6 Hz, 1H), 7.59 (d, *J* = 8.8 Hz, 2H), 7.42 (d, *J* = 15.6 Hz, 1H), 7.03–6.87 (m, 4H), 3.87 (s, 3H), 3.83 (s, 3H).

^{13}C NMR (63 MHz, $CDCl_3$) δ 188.8, 163.3, 161.6, 143.9, 131.4, 130.8, 130.2, 127.9, 119.6, 114.4, 113.9, 55.6, 55.5. The spectroscopic data were consistent with those reported in the literature.

***(E)*-3-(4-Bromophenyl)-1-(4-methoxyphenyl)-2-propen-1-one 3l (table 2, entry 12)** [10]
Pale orange solid (73%). ^{1}H NMR (250 MHz, $CDCl_3$) δ 8.03 (d, *J* = 8.9 Hz, 2H), 7.73 (d, *J* = 15.6 Hz, 1H), 7.62–7.43 (m, 5H), 6.98 (d, *J* = 8.9 Hz, 2H), 3.89 (s, 3H). ^{13}C NMR (63 MHz, $CDCl_3$) δ 188.5, 163.7, 142.6, 134.1, 132.3, 131.0, 131.0, 129.9, 124.7, 122.5, 114.0, 55.7. The spectroscopic data were consistent with those reported in the literature.

***(E)*-3-Phenyl-1-(4-methylphenyl)-2-propen-1-one 3m (table 2, entry 13)** [11]
Pale yellow solid (95%). ^{1}H NMR (250 MHz, $CDCl_3$) δ 8.01–7.94 (m, 2H), 7.85 (d, *J* = 15.7 Hz, 1H), 7.71–7.64 (m, 2H), 7.57 (d, *J* = 15.7 Hz, 1H), 7.49–7.42 (m, 3H), 7.34 (dd, *J* = 8.5, 0.6 Hz, 2H), 2.47 (s, 3H). ^{13}C NMR (63 MHz, $CDCl_3$) δ 190.1, 144.5, 143.8, 135.7, 135.1, 130.5, 129.5, 129.1, 128.8, 128.5, 122.2, 21.8. The spectroscopic data were consistent with those reported in the literature.

***(E)*-3-(4-Nitrophenyl)-1-(4-methoxyphenyl)-2-propen-1-one 3n (table 2, entry 14)** [12]
Pale yellow solid (55%). ^{1}H NMR (250 MHz, $CDCl_3$) δ 8.24 (d, *J* = 8.8 Hz, 1H), 8.02 (d, *J* = 8.9 Hz, 1H), 7.83–7.72 (m, 1H), 7.63 (d, *J* = 15.7 Hz, 1H), 6.97 (d, *J* = 8.9 Hz, 1H), 3.87 (s, 1H). ^{13}C NMR (63 MHz, $CDCl_3$) δ = 187.8, 163.9, 148.4, 141.3, 140.8, 131.1, 130.5, 128.9, 125.7, 124.2, 114.1, 55.6. The spectroscopic data were consistent with those reported in the literature.

***(E)*-3-(2-Methoxyphenyl)-1-(4-methylphenyl)-2-propen-1-one 3o (table 2, entry 15)** [13]
Yellow oil (93%). ^{1}H NMR (250 MHz, $CDCl_3$) δ 8.16 (d, *J* = 15.9 Hz, 1H), 8.02–7.94 (m, 2H), 7.73–7.61 (m, 2H), 7.45–7.36 (m, 1H), 7.37–7.30 (m, 2H), 7.09–6.93 (m, 2H), 3.94 (s, 3H), 2.46 (s, 3H). ^{13}C NMR (63 MHz, $CDCl_3$) δ 190.7, 158.8, 143.4, 140.0, 136.0, 131.7, 129.3, 129.2, 128.8, 124.1, 122.9, 120.8, 111.3, 55.6, 21.8. The spectroscopic data were consistent with those reported in the literature.

***(E)*-3-(4-Methylphenyl)-1-(4-methylphenyl)-2-propen-1-one 3p (table 2, entry 16)** [14]
Pale yellow solid (77%). ^{1}H NMR (250 MHz, $CDCl_3$) δ 7.97 (d, *J* = 8.2 Hz, 1H), 7.89–7.78 (m, 1H), 7.61–7.45 (m, 2H), 7.33 (d, *J* = 7.9 Hz, 1H), 7.25 (d, *J* = 8.0 Hz, 1H), 2.46 (s, 3H), 2.42 (s, 3H). ^{13}C NMR (63 MHz, $CDCl_3$) δ 190.2, 144.6, 143.6, 141.0, 135.8, 132.3, 129.8, 129.4, 128.7, 128.5, 121.1, 21.8, 21.6. The spectroscopic data were consistent with those reported in the literature.

***(E)*-3-(4-Bromophenyl)-1-(4-methylphenyl)-2-propen-1-one 3q (table 2, entry 17)** [15]
Pale yellow solid (76%). ^{1}H NMR (250 MHz, $CDCl_3$) δ 8.01–7.92 (m, 1H), 7.76 (d, *J* = 15.7 Hz, 1H), 7.62–7.48 (m, 5H), 7.38–7.30 (m, 2H), 2.46 (s, 3H). ^{13}C NMR (63 MHz, $CDCl_3$) δ 189.8, 144.0, 143.0, 135.5, 134.0, 132.3, 129.9, 129.5, 128.8, 124.8, 122.6, 21.8. The spectroscopic data were consistent with those reported in the literature.

***(E)*-3-(2-Nitrophenyl)-1-(4-methylphenyl)-2-propen-1-one 3r (table 2, entry 18)** [16]
White solid (66%). ^{1}H NMR (250 MHz, $CDCl_3$) δ 7.92 (d, *J* = 15.9 Hz, 1H), 7.89–7.84 (m, 1H), 7.78–7.68 (m, 2H), 7.60–7.44 (m, 2H), 7.41–7.29 (m, 1H), 7.19–7.03 (m, 3H), 2.24 (s, 3H). ^{13}C NMR

(63 MHz, $CDCl_3$) δ 190.0, 148.6, 144.2, 139.8, 134.9, 133.7, 131.5, 130.4, 129.5, 129.3, 129.0, 127.4, 125.1, 21.8. The spectroscopic data were consistent with those reported in the literature.

***(E)*-1-(4-Chlorophenyl)-3-phenyl-2-propen-1-one 3s (table 2, entry 19)** [4]
Pale yellow solid (54%). ^{1}H NMR (250 MHz, CDCl3) δ 7.97 (d, *J* = 8.6 Hz, 2H), 7.82 (d, *J* = 15.7 Hz, 1H), 7.68–7.60 (m, 2H), 7.50 (d, *J* = 6.6 Hz, 2H), 7.43 (dd, *J* = 7.1, 3.9 Hz, 4H). ^{13}C NMR (63 MHz, $CDCl_3$) δ 189.2, 145.4, 139.3, 136.6, 134.8, 130.8, 130.0, 129.1, 129.0, 128.6, 121.5. The spectroscopic data were consistent with those reported in the literature.

***(E)*-1-(4-Chlorophenyl)-3-(3-methoxyphenyl)-2-propen-1-one 3t (table 2, entry 20)** [4]
Pale yellow solid (78%). ^{1}H NMR (250 MHz, $CDCl_3$) δ 8.04–7.96 (m, 2H), 7.81 (d, *J* = 15.7 Hz, 1H), 7.55–7.45 (m, 3H), 7.43–7.33 (m, 1H), 7.29–7.24 (m, 1H), 7.20–7.16 (m, 1H), 7.01 (ddd, *J* = 8.1, 2.6, 1.0 Hz, 1H), 3.89 (s, 3H). ^{13}C NMR (63 MHz, $CDCl_3$) δ 189.3, 160.1, 145.4, 139.4, 136.6, 136.2, 130.1, 130.0, 129.1, 121.9, 121.3, 116.6, 113.6, 55.5. The spectroscopic data were consistent with those reported in the literature.

***(E)*-1-(4-Chlorophenyl)-3-(4-bromophenyl)-2-propen-1-one 3u (table 2, entry 21)** [17]
Pale yellow solid (64%). ^{1}H NMR (250 MHz, $CDCl_3$) δ 8.03–7.95 (m, 1H), 7.77 (d, *J* = 15.7 Hz, 1H), 7.62–7.56 (m, 1H), 7.56–7.45 (m, 3H). ^{13}C NMR (63 MHz, $CDCl_3$) δ 189.0, 144.0, 139.5, 136.4, 133.7, 132.4, 130.0, 130.0, 129.1, 125.2, 122.0. The spectroscopic data were consistent with those reported in the literature.

***(E)*-1-(4-Chlorophenyl)-3-(2-nitrophenyl)-2-propen-1-one 3v (table 2, entry 22)** [16]
Brown solid (48%). ^{1}H NMR (250 MHz, $CDCl_3$) δ 8.17 (d, *J* = 15.7 Hz, 1H), 8.16–8.09 (m, 1H), 8.04–7.96 (m, 2H), 7.79–7.68 (m, 2H), 7.67–7.57 (m, 1H), 7.56–7.48 (m, 1H), 7.30 (d, *J* = 15.7 Hz, 1H). ^{13}C NMR (63 MHz, $CDCl_3$) δ 189.5, 148.8, 140.9, 139.8, 135.8, 133.8, 131.4, 130.7, 130.4, 129.4, 129.2, 127.0, 125.2. The spectroscopic data were consistent with those reported in the literature.

***(E)*-1-(4-Bromophenyl)-3-phenyl-2-propen-1-one 3w (table 2, entry 23)** [4]
Pale yellow solid (60%). ^{1}H NMR (250 MHz, $CDCl_3$) δ 7.96–7.89 (m, 1H), 7.85 (d, *J* = 15.7 Hz, 1H), 7.72–7.64 (m, 4H), 7.51 (d, *J* = 15.7 Hz, 1H), 7.49–7.42 (m, 3H). ^{13}C NMR (63 MHz, $CDCl_3$) δ 189.5, 145.6, 137.1, 134.8, 132.1, 130.9, 130.2, 129.2, 128.7, 128.1, 121.6. The spectroscopic data were consistent with those reported in the literature.

***(E)*-1-(4-Bromophenyl)-3-(4-bromophenyl)-2-propen-1-one 3x (table 2, entry 24)** [18]
Pale yellow solid (86%). ^{1}H NMR (250 MHz, $CDCl_3$) δ 7.96–7.89 (m, 1H), 7.85 (d, *J* = 15.7 Hz, 1H), 7.72–7.64 (m, 4H), 7.51 (d, *J* = 15.7 Hz, 1H), 7.49–7.42 (m, 3H). ^{13}C NMR (63 MHz, $CDCl_3$) δ 189.2, 144.1, 136.8, 133.7, 132.4, 132.1, 130.1, 130.0, 128.2, 125.2, 122.0. The spectroscopic data were consistent with those reported in the literature.

***(E)*-1-(4-Bromophenyl)-3-(4-nitrophenyl)-2-propen-1-one 3y (table 2, entry 25)** [19]
Pale yellow solid (49%). ^{1}H NMR (250 MHz, CDCl3) δ 8.28 (d, *J* = 8.8 Hz, 1H), 7.90 (d, *J* = 8.6 Hz, 1H), 7.87–7.74 (m, 2H), 7.67 (d, *J* = 8.6 Hz, 1H), 7.59 (d, *J* = 15.7 Hz, 1H). ^{13}C NMR (63 MHz,

$CDCl_3$) δ = 188.6, 148.8, 142.2, 140.9, 136.3, 132.3, 130.2, 129.1, 128.8, 125.2, 124.4. The spectroscopic data were consistent with those reported in the literature.

***(E)*-1-(4-Nitrophenyl)-3-phenyl-2-propen-1-one 3z (table 2, entry 26)** [10]
Yellow solid (60%). ^1H NMR (250 MHz, CDCl3) δ 8.36 (d, *J* = 9.0 Hz, 2H), 8.15 (d, *J* = 9.0 Hz, 2H), 7.86 (d, *J* = 15.7 Hz, 1H), 7.67 (dd, *J* = 6.8, 2.8 Hz, 2H), 7.54–7.39 (m, 4H). ^{13}C NMR (63 MHz, $CDCl_3$) δ 189.2, 150.2, 147.0, 143.2, 134.4, 131.4, 129.6, 129.3, 128.9, 124.0, 121.4. The spectroscopic data were consistent with those reported in the literature.

***(E)*-3-(4-Methoxyphenyl)-1-(4-nitrophenyl)-2-propen-1-one 3aa (table 2, entry 27)** [20]
Pale yellow solid (64%). ^1H NMR (250 MHz, CDCl3) δ 8.35 (d, *J* = 8.9 Hz, 2H), 8.13 (d, *J* = 8.9 Hz, 2H), 7.82 (d, *J* = 15.6 Hz, 1H), 7.62 (d, *J* = 8.8 Hz, 2H), 7.36 (d, *J* = 15.6 Hz, 1H), 6.96 (d, *J* = 8.8 Hz, 2H), 3.87 (s, 3H). ^{13}C NMR (63 MHz, $CDCl_3$) δ 189.2, 162.4, 150.1, 146.9, 143.6, 130.8, 129.5, 127.2, 124.0, 119.1, 114.7, 55.6. The spectroscopic data were consistent with those reported in the literature.

***(E)*-3-(4-Methylphenyl)-1-(4-nitrophenyl)-2-propen-1-one 3ab (table 2, entry 28)** [21]
Pale yellow solid (77%). ^1H NMR (250 MHz, CDCl3) δ 8.35 (d, *J* = 9.0 Hz, 2H), 8.14 (d, *J* = 9.0 Hz, 2H), 7.83 (d, *J* = 15.7 Hz, 1H), 7.56 (d, *J* = 8.2 Hz, 2H), 7.44 (d, *J* = 15.7 Hz, 1H), 7.25 (d, *J* = 8.0 Hz, 2H), 2.41 (s, 3H). ^{13}C NMR (63 MHz, $CDCl_3$) δ 189.3, 150.1, 147.1, 143.4, 142.2, 131.7, 130.0, 129.5, 128.9, 124.0, 120.4, 21.8. The spectroscopic data were consistent with those reported in the literature.

***(E)*-3-(4-Bromophenyl)-1-(4-nitrophenyl)-2-propen-1-one 3ac (table 2, entry 29)** [19]
Pale yellow solid (68%). ^1H NMR (250 MHz, CDCl3) δ 8.36 (d, *J* = 9.0 Hz, 2H), 8.14 (d, *J* = 9.0 Hz, 2H), 7.79 (d, *J* = 15.7 Hz, 1H), 7.59 (d, *J* = 8.7 Hz, 2H), 7.52 (d, *J* = 8.7 Hz, 2H), 7.44 (d, *J* = 15.7 Hz, 1H). ^{13}C NMR (63 MHz, $CDCl_3$) δ 188.9, 150.3, 145.5, 142.9, 133.3, 132.5, 130.2, 129.6, 125.8, 124.1, 121.8. The spectroscopic data were consistent with those reported in the literature.

***(E)*-3-(4-Nitrophenyl)-1-(4-nitrophenyl)-2-propen-1-one 3ad (table 2, entry 30)** [22]
Pale yellow solid (60%), mp 204-206. ^1H NMR (250 MHz, DMSO-d_6) δ 8.42 (s, 4H), 8.33 (d, *J* = 8.8 Hz, 2H), 8.23 (d, *J* = 8.8 Hz, 2H), 8.18 (d, *J* = 15.7 Hz, 1H), 7.90 (d, *J* = 15.7 Hz, 1H). ^{13}C NMR (63 MHz, DMSO-d_6) δ 188.3, 150.1, 148.3, 142.5, 141.8, 140.9, 130.2, 130.1, 125.8, 124.0, 124.0. The spectroscopic data were consistent with those reported in the literature.

***(E)*-3-(5-Chloro-2-nitrophenyl)-1-phenyl-2-propen-1-one 3ae (table 2, entry 31)** [23]
White solid (57%). ^1H NMR (250 MHz, $CDCl_3$) δ: 8.11 (1H, d, *J* = 15.7 Hz), 8.03 (2H, d, *J* = 8.8 Hz), 7.70 (1H, d, *J* = 2.8 Hz), 7.64–7.45 (5H, m), 7.33 (1H, d, *J* = 15.7 Hz) ^{13}C NMR (63 MHz, $CDCl_3$) δ: 190.1, 146.7, 140.3, 139.1, 137.2, 133.5, 133.4, 130.3, 129.3, 128.9, 128.2, 126.7. The spectroscopic data were consistent with those reported in the literature.

***(E)*-3-(4,5-Dimethoxy-2-nitrophenyl)-1-phenyl-2-propen-1-one 3af (table 2, entry 32)** [24]
White solid (63%). ^1H NMR (250 MHz, $CDCl_3$) δ: 8.20 (1H, d, *J* = 15.7 Hz), 8.08–7.94 (2H, m), 7.67 (1H, s), 7.64–7.43 (3H, m), 7.17 (1H, d, *J* = 15.7 Hz), 7.05 (1H, s), 4.03 (3H, s), 3.99 (3H, s). ^{13}C

NMR (63 MHz, $CDCl_3$) δ: 191.6, 153.4, 150.1, 141.7, 141.5, 137.6, 133.2, 129.0, 128.8, 126.8, 126.2, 110.2, 108.1, 56.8, 56.7. The spectroscopic data were consistent with those reported in the literature.

***(E)*-1-Phenyl-3-(furan-2-yl)-2-propen-1-one 3aj (table 2, entry 36)** [25]
White solid (85%). ^{1}H NMR (250 MHz, $CDCl_3$) δ: 7.88 (1H, d, *J* = 15.7 Hz), 7.65 (3H, q, *J* = 2.8 Hz), 7.50–7.35 (4H, m), 7.34 (1H, d, *J* = 3.8 Hz), 6.60 (1H, dd, *J* = 3.8, 2.7 Hz). ^{13}C NMR (63 MHz, $CDCl_3$) δ 178.2, 153.8, 146.7, 144.2, 134.8, 130.8, 129.1, 128.7, 121.2, 117.7, 112.7. The spectroscopic data were consistent with those reported in the literature.

***(E)*-3-Phenyl-1-(furan-2-yl)-2-propen-1-one 3ak (table 2, entry 37)** [26]
White solid (60%). IR ν_{max} (KBr): 3120, 1660, 1602, 1552 cm^{-1} ^{1}H NMR (250 MHz, $CDCl_3$) δ. 8.03 (2H, dd, *J* = 7, 2 Hz), 7.65–7.38 (6H, m), 6.72 (1H, d, *J* = 3 Hz), 6.52 (1H, dd, *J* = 3, 2 Hz). ^{13}C NMR (63 MHz, $CDCl_3$) δ 190.3, 152.2, 145.5, 138.7, 133.3, 131.2, 129.1, 128.9, 119.8, 116.8, 113.2. The spectroscopic data were consistent with those reported in the literature.

***(E)*-1,3-bis(Furan-2-yl)-2-propen-1-one 3al (table 2, entry 38)** [27]
White solid (97%). IR ν_{max} (KBr): 3124, 1659, 1600, 1463 cm^{-1}.^{1}H NMR (250 MHz, $CDCl_3$) δ 7.56 (1H, dd, *J* = 2, 1 Hz), 7.54 (1H, d, *J* = 15 Hz), 7.44 (1H, d, *J* = 1 Hz), 7.28–7.13 (2H, m), 6.63 (1H, d, *J* = 3 Hz), 6.49 (1H, dd, *J* = 4, 2 Hz), 6.42 (1H, dd, *J* = 3, 2 Hz). ^{13}C NMR (63 MHz, $CDCl_3$) δ 177.9, 153.8, 151.7, 146.7, 145.2, 130.0, 118.9, 117.6, 116.5, 112.8, 112.6. The spectroscopic data were consistent with those reported in the literature.

***(E)*-1-(Furan-2-yl)-3-(2-nitrophenyl)-2-propen-1-one 3am (table 2, entry 39)** [27]
White solid (45%). IR ν_{max} (KBr): 1659, 1610, 1519 cm^{-1}. ^{1}H NMR (250 MHz, $CDCl_3$) δ 8.18 (1H, d, *J* = 16 Hz), 8.00 (1H, dd, *J* = 8, 1 Hz), 7.72–7.57 (3H, m), 7.54–7.45 (1H, m), 7.31 (1H, dd, *J* = 4, 1 Hz), 7.20 (1H, s), 6.55 (1H, dd, *J* = 4, 2 Hz). ^{13}C NMR (63 MHz, CDCl3) δ 177.5, 153.3, 148.8, 147.1, 139.3, 133.6, 131.3, 130.5, 129.4, 126.4, 125.1, 118.6, 112.9. The spectroscopic data were consistent with those reported in the literature.

***(E)*-3-Phenyl-1-(thiophen-2-yl)-2-propen-1-one 3an (table 2, entry 40)** [28]
White solid (54%). IR ν_{max} (KBr): 1651, 1592, 1414 cm^{-1}. ^{1}H NMR (250 MHz, $CDCl_3$) δ 7.87 (1H, dd), 7.85 (1H, d, *J* = 15 Hz), 7.69 (1H, dd, *J* = 5, 1 Hz), 7.67–7.61 (2H, m), 7.48–7.38 (3H, m), 7.19 (1H, dd, *J* = 5, 4 Hz). ^{13}C NMR (63 MHz, $CDCl_3$) δ 182.2, 145.6, 144.3, 134.8, 134.1, 132.0, 130.7, 129.1, 128.6, 128.4, 121.7. The spectroscopic data were consistent with those reported in the literature.

***(E)*-1-Phenyl-3-(thiophen-2-yl)-2-propen-1-one 3ao (table 2, entry 41)** [29]
White solid (78%). IR ν_{max} (KBr): 3086, 1659, 1588 cm^{-1}. ^{1}H NMR (250 MHz, $CDCl_3$) δ 8.06–7.87 (3H, m), 7.53 (2H, dt, *J* = 14, 7 Hz), 7.42 (1H, d, *J* = 5 Hz), 7.33 (1H, d, *J* = 15 Hz), 7.12–7.04 (1H, m). ^{13}C NMR (63 MHz, $CDCl_3$) δ 190.1, 140.5, 138.2, 137.4, 135.4, 132.9, 132.2, 129.0, 128.7, 128.5, 120.9. The spectroscopic data were consistent with those reported in the literature.

***(E)*-1,3-bis(Thiophen-2-yl)-2-propen-1-one 3ap (table 2, entry 42)** [30]

White solid (78%). IR ν_{max} (KBr): 1639, 1576, 1410 cm^{-1}. ^{1}H NMR (250 MHz, $CDCl_3$) δ 7.96 (1H, d, *J* = 15 Hz), 7.84 (1H, dd, *J* = 4, 1 Hz), 7.67 (1H, dd, *J* = 5, 1 Hz), 7.42 (1H, d, *J* = 5 Hz), 7.36 (0H, d, *J* = 4 Hz), 7.21 (1H, d, *J* = 16 Hz), 7.20–7.15 (1H, m), 7.09 (1H, dd, *J* = 5, 4 Hz). ^{13}C NMR (63 MHz, $CDCl_3$) δ 181.7, 180.6, 145.6, 140.2, 136.6, 134.0, 132.3, 131.8, 129.0, 128.5, 128.4, 120.5. The spectroscopic data were consistent with those reported in the literature.

(2*E*,6*E*)-2,6-Dibenzylidenecyclohexanone 5 [31]

White solid (92%). IR ν_{max} (KBr): cm^{-1}. ^{1}H NMR (250 MHz, $CDCl_3$) δ 7.81 (s, 2H), 7.52–7.32 (m, 10H), 2.94 (td, *J* = 6.6, 2.0 Hz, 4H), 1.79 (p, *J* = 6.3 Hz, 2H). ^{13}C NMR (63 MHz, $CDCl_3$) δ 190.5, 137.1, 136.3, 136.1, 130.5, 128.7, 128.5, 28.6, 23.2. The spectroscopic data were consistent with those reported in the literature.

(1*E*,3*E*)-1,3-Dibenzylidene-3,4-dihydronaphthalen-2(1*H*)-one 6

white solid (86%). Mp 152–154 °C (lit. 154–156 °C) [32]; IR ν_{max} (KBr): cm^{-1}. ^{1}H NMR (250 MHz, $CDCl_3$). δ 7.64 (t, *J* = 1.8 Hz, 1H), 7.44 (s, 1H), 7.33–7.27 (m, 3H), 7.24–7.13 (m, 2H), 7.08 (dd, *J* = 7.2, 3.9 Hz, 4H), 7.02–6.93 (m, 2H), 6.87–6.76 (m, 1H), 3.91 (d, *J* = 1.7 Hz, 2H).^{13}C NMR (63 MHz, $CDCl_3$) δ 191.7, 137.0, 135.8, 135.7, 135.4, 135.3, 132.0, 131.4, 130.3, 129.7, 129.0, 128.7, 128.6, 128.4, 128.1, 126.5, 32.7.

(3*E*,5*E*)-3,5-Dibenzylidene-1-methylpiperidin-4-one 7 [33]

White solid (74%). IR ν_{max} (KBr): 3059, 2784, 1651, 1614 cm^{-1}. ^{1}H NMR (250 MHz, $CDCl_3$) δ 7.83 (s, 1H), 7.51–7.34 (m, 4H), 3.78 (d, *J* = 1.4 Hz, 2H), 2.47 (s, 1H). ^{13}C NMR (63 MHz, $CDCl_3$) δ 187.0, 136.7, 135.3, 133.0, 130.5, 129.2, 128.7, 57.1, 45.8. The spectroscopic data were consistent with those reported in the literature.

3-Benzylidenepentane-2,4-dione 8 [34]

As a yellow solid (92%). ^{1}H NMR (250 MHz, $CDCl_3$) δ 7.49 (s, 1H), 7.40 (s, 5H), 2.43 (s, 3H), 2.29 (s, 3H). ^{13}C NMR (63 MHz, $CDCl_3$) δ 205.8, 196.6, 142.9, 139.9, 133.0, 130.8, 129.8, 129.2, 31.8, 26.7. The spectroscopic data were consistent with those reported in the literature.

2. ^{1}H and ^{13}C NMR Spectra of All Compounds

Figure S1. *(E)*-3-(4-methoxyphenyl)-1-phenyl-2-propen-1-one **3b**.

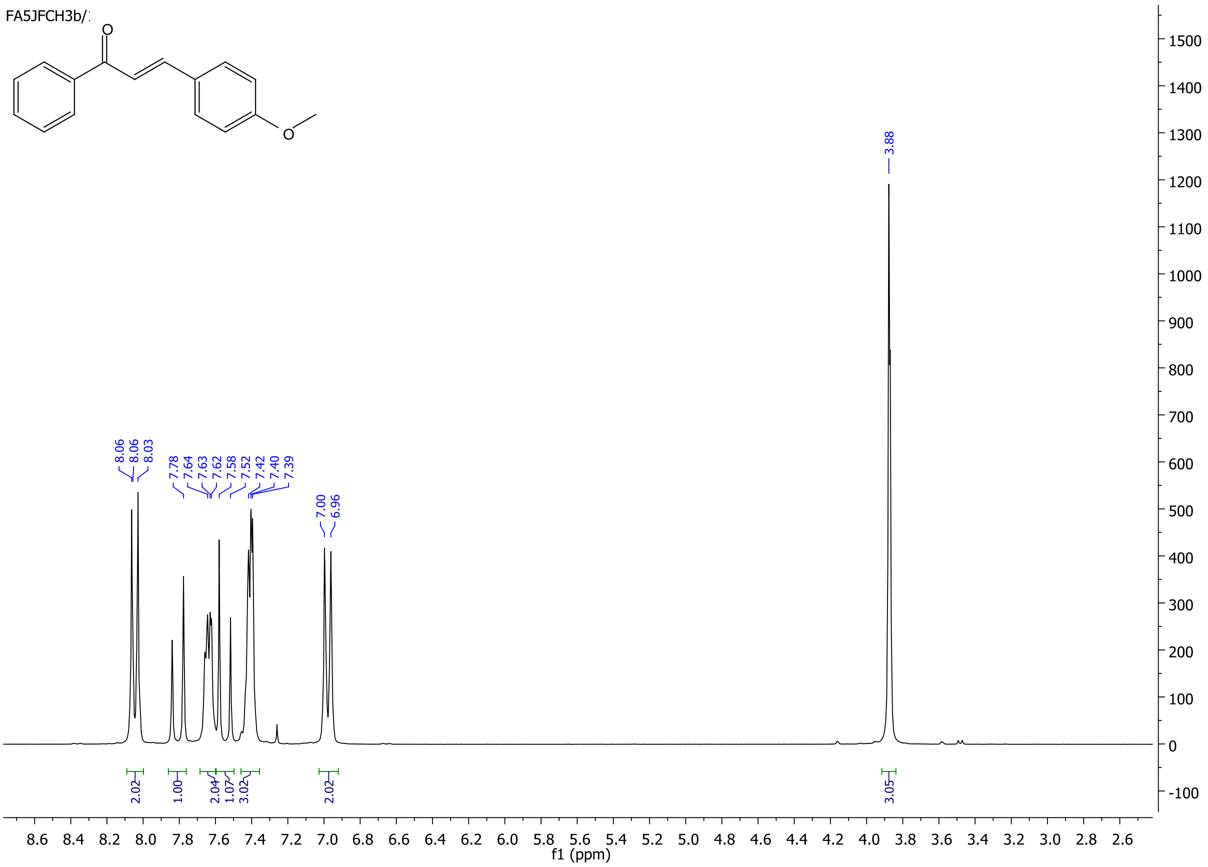

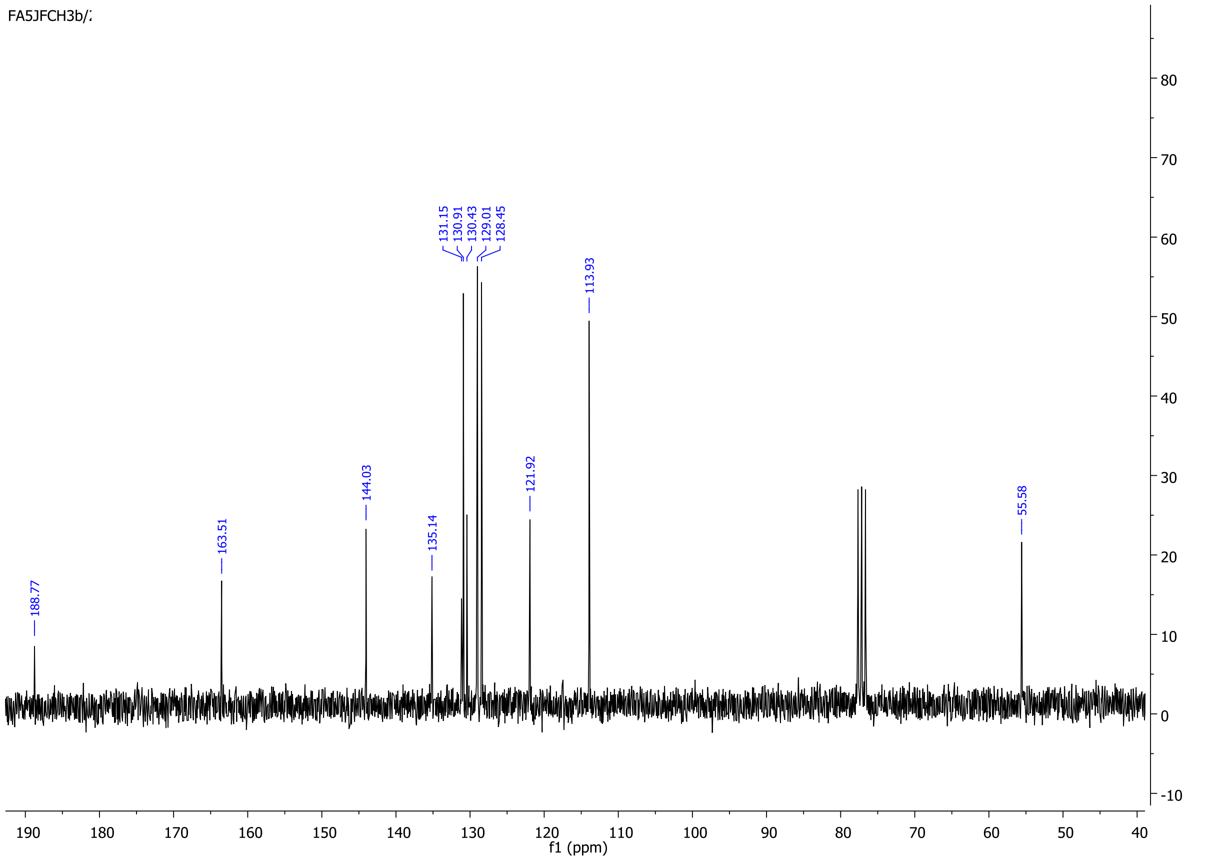

Figure S2. (*E*)-1-phenyl-3-(*p*-tolyl)-2-propen-1-one **3c**.

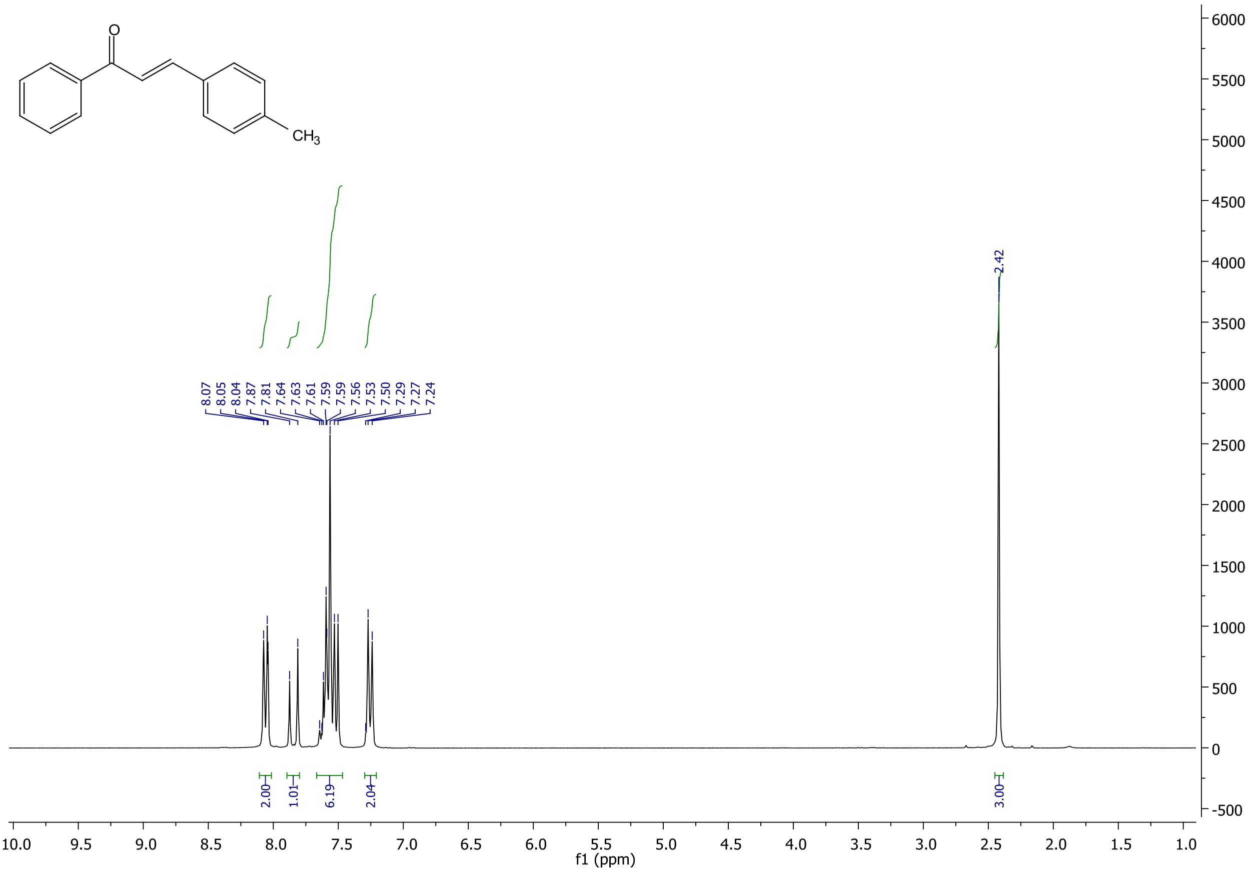

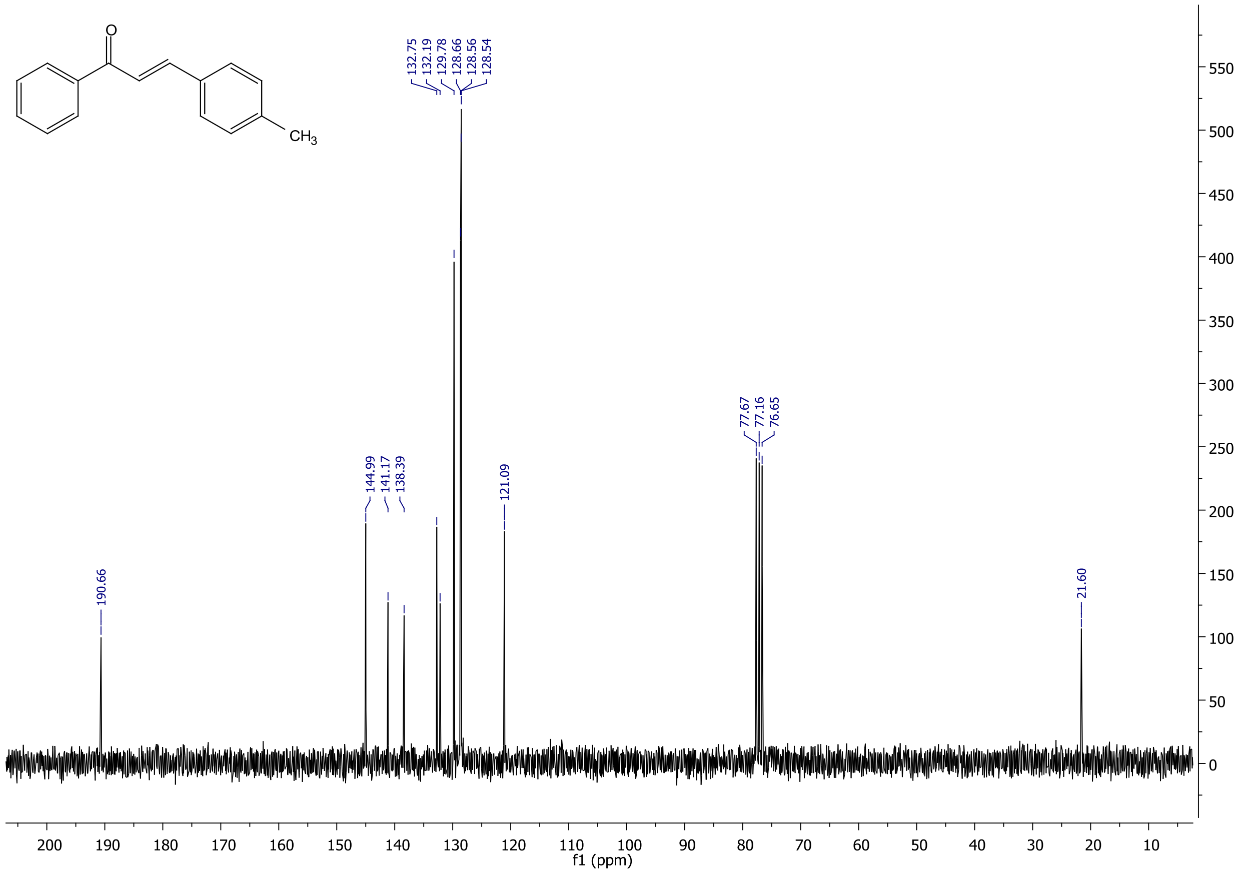

Figure S3. (*E*)-3-(4-chlorophenyl)-1-phenyl-2-propen-1-one **3d**.

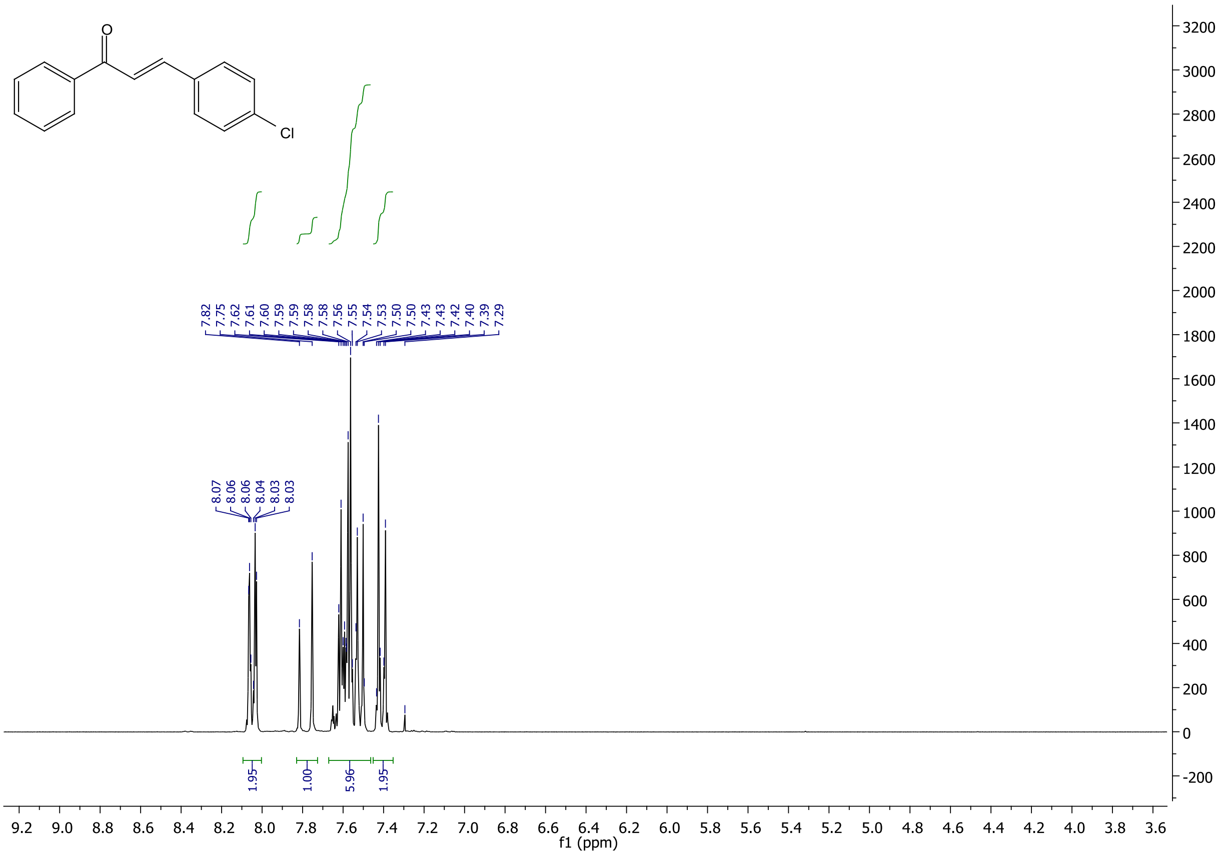

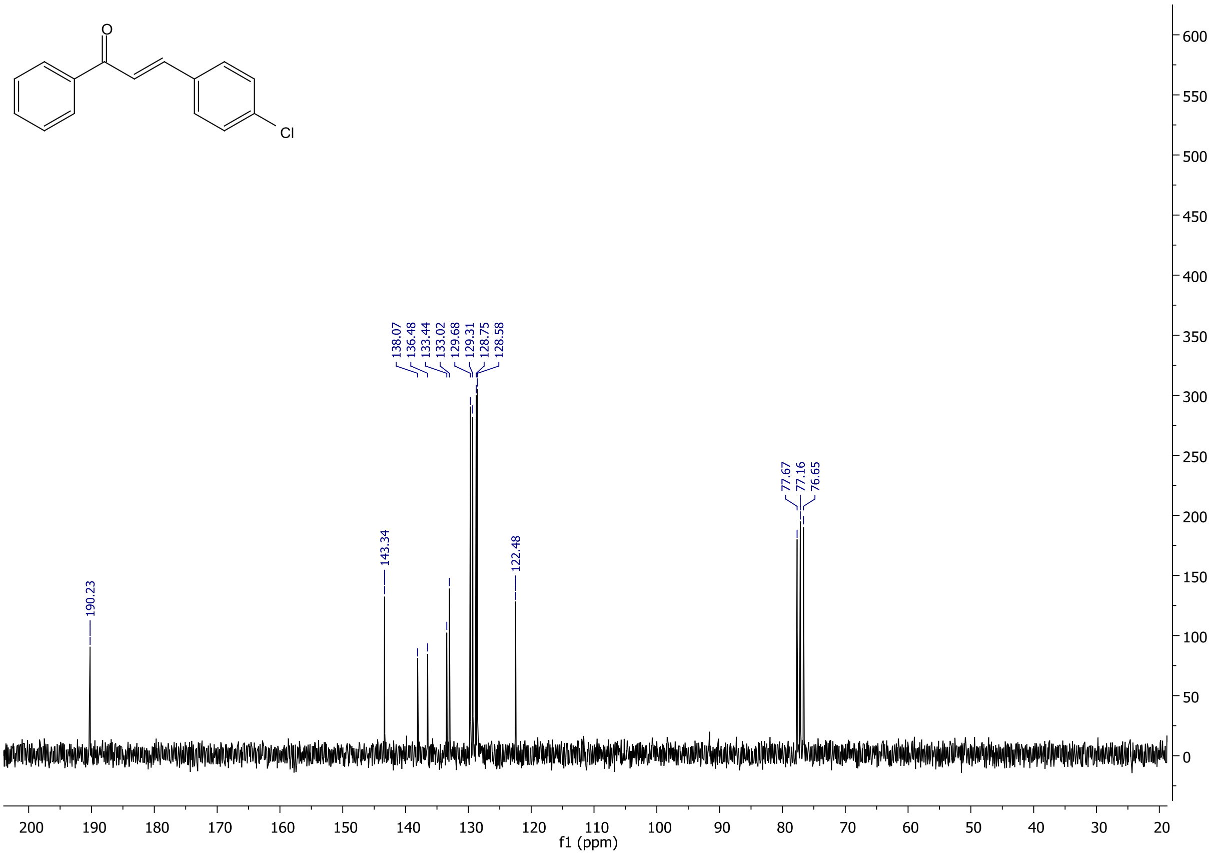

Figure S4. (*E*)-3-(2-chlorophenyl)-1-phenyl-2-propen-1-one **3e**.

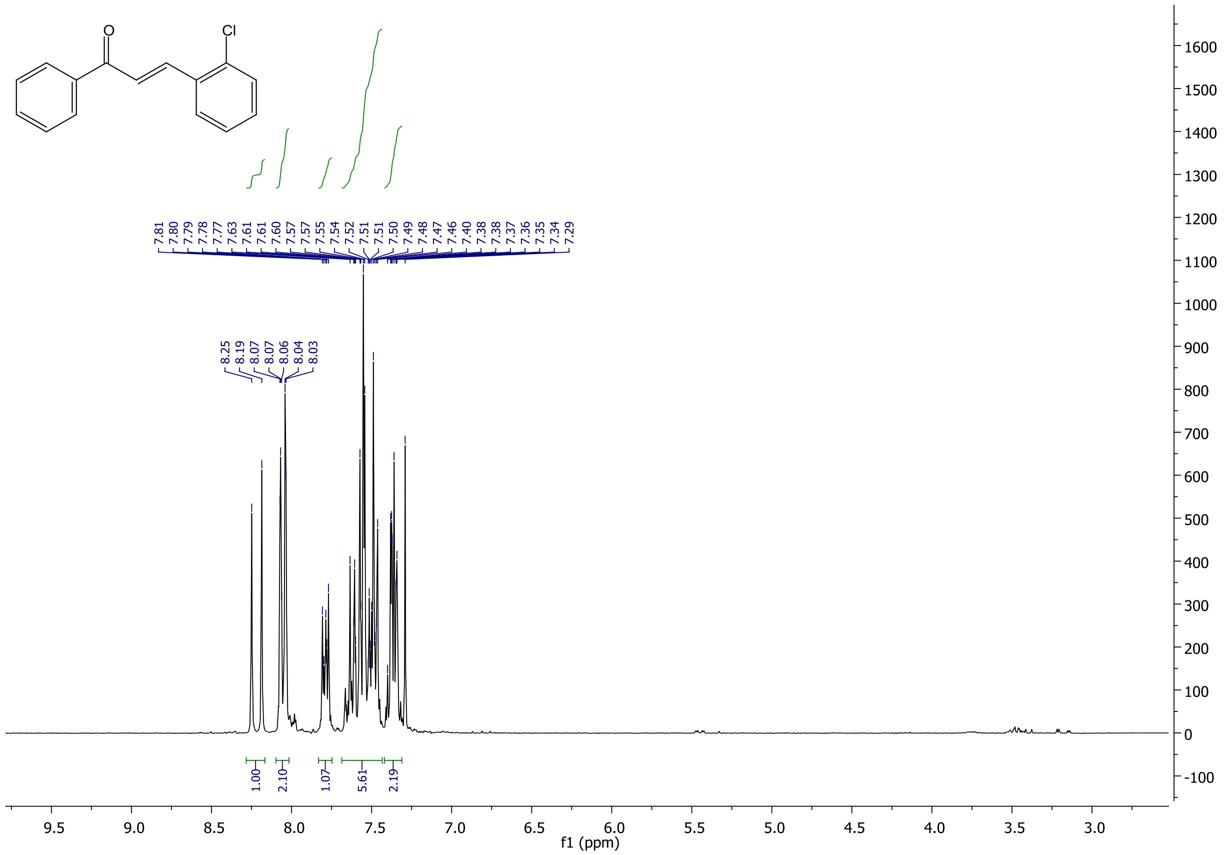

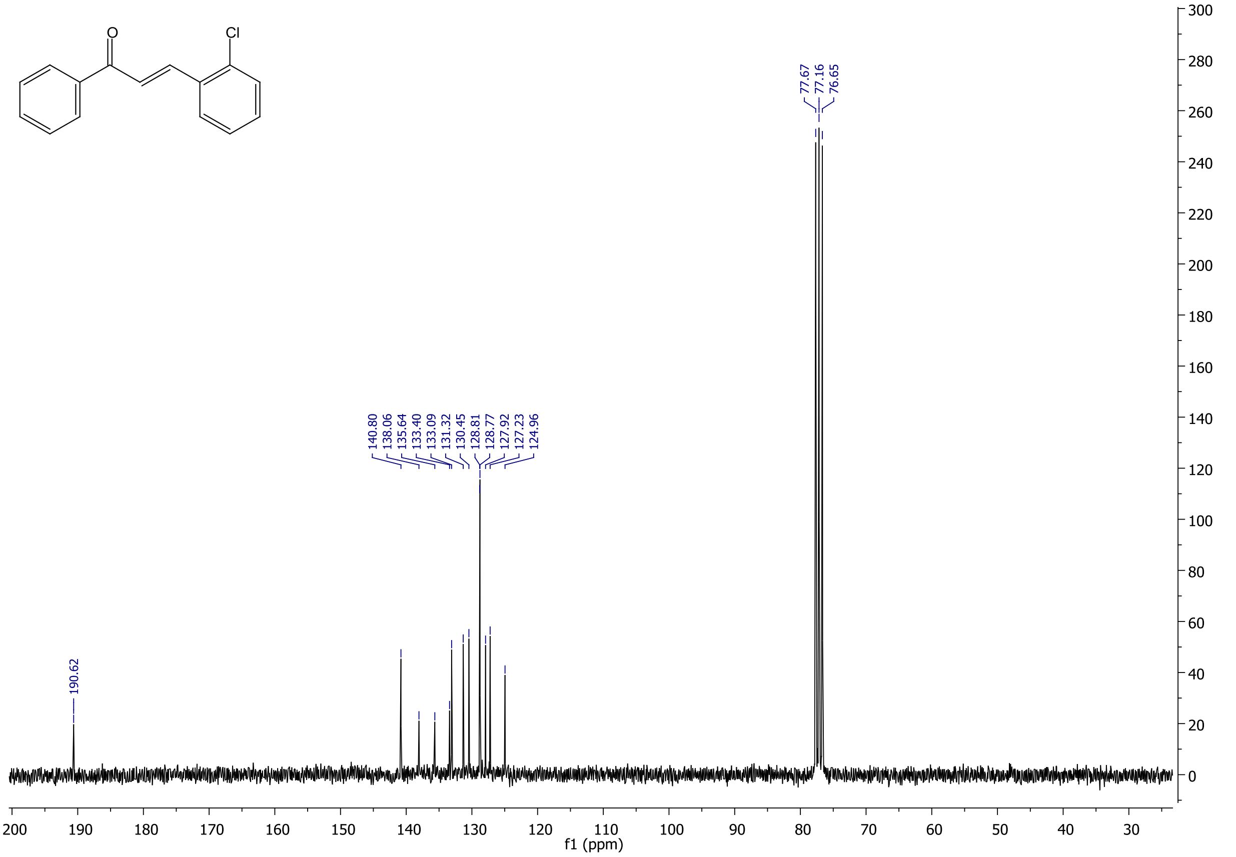

Figure S5. (*E*)-3-(4-Bromophenyl)-1-phenyl-2-propen-1-one **3f**.

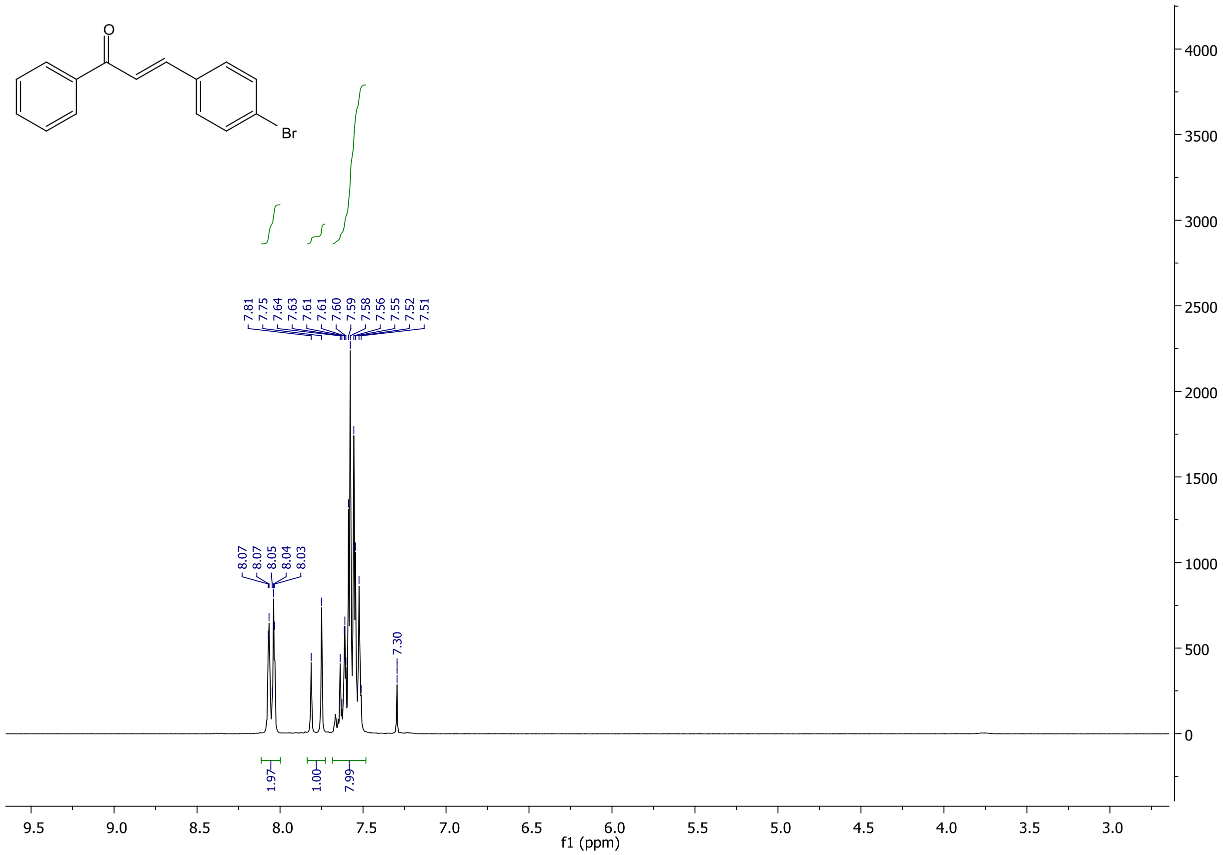

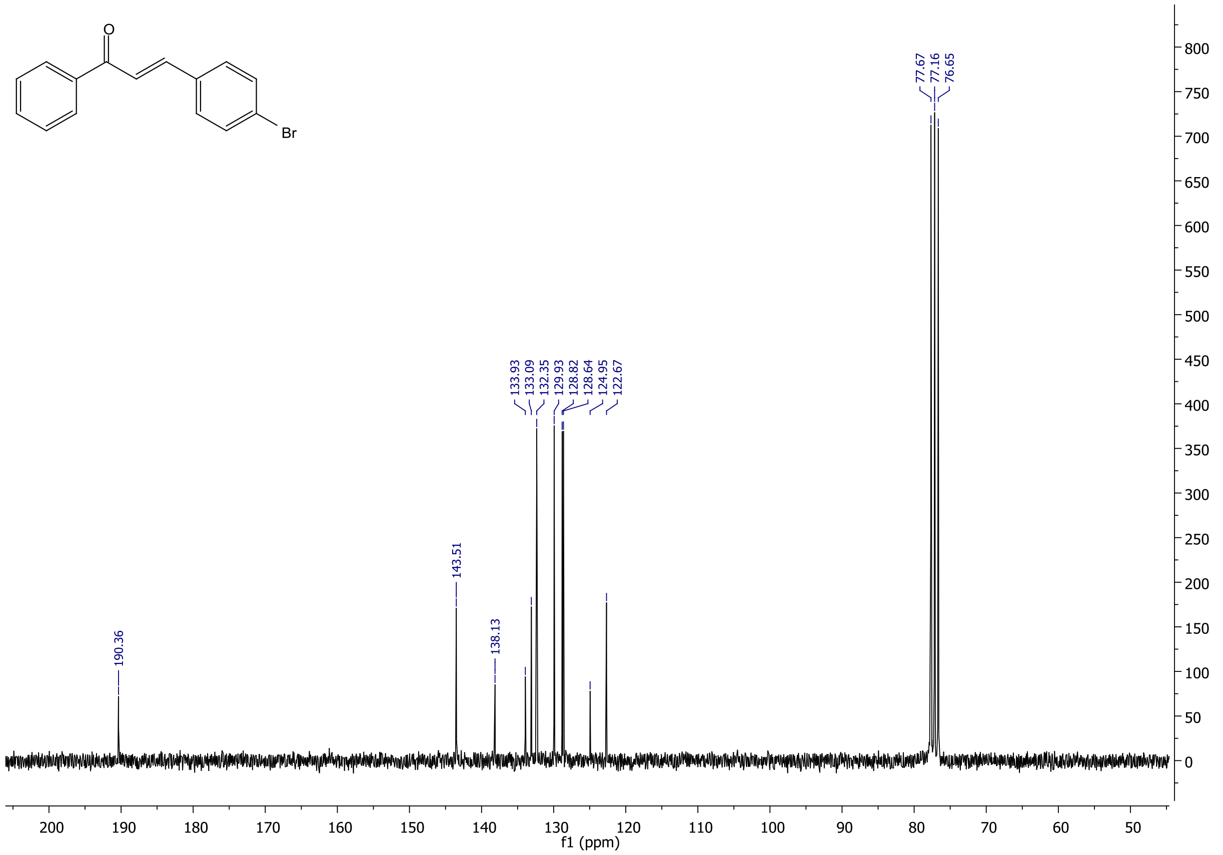

Figure S6. (*E*)-3-(2-nitrophenyl)-1-phenyl-2-propen-1-one **3g**.

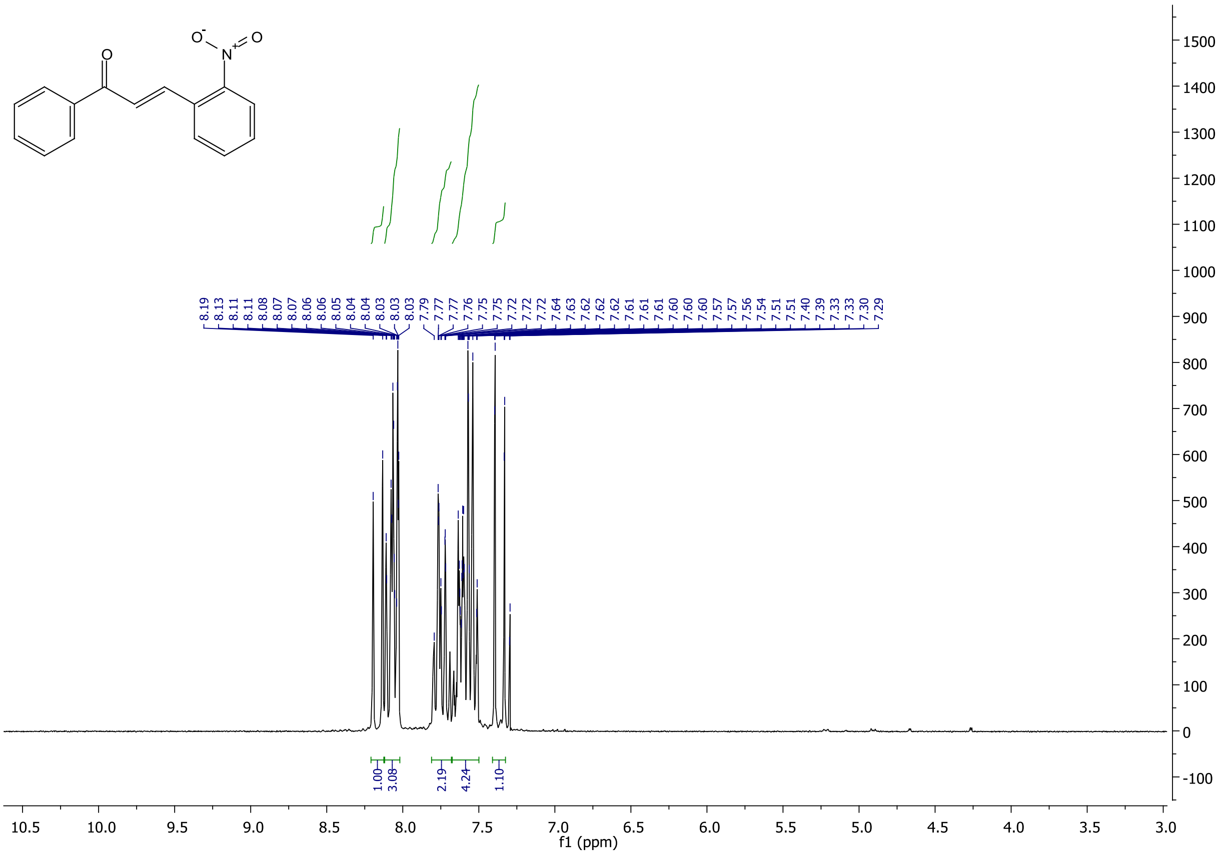

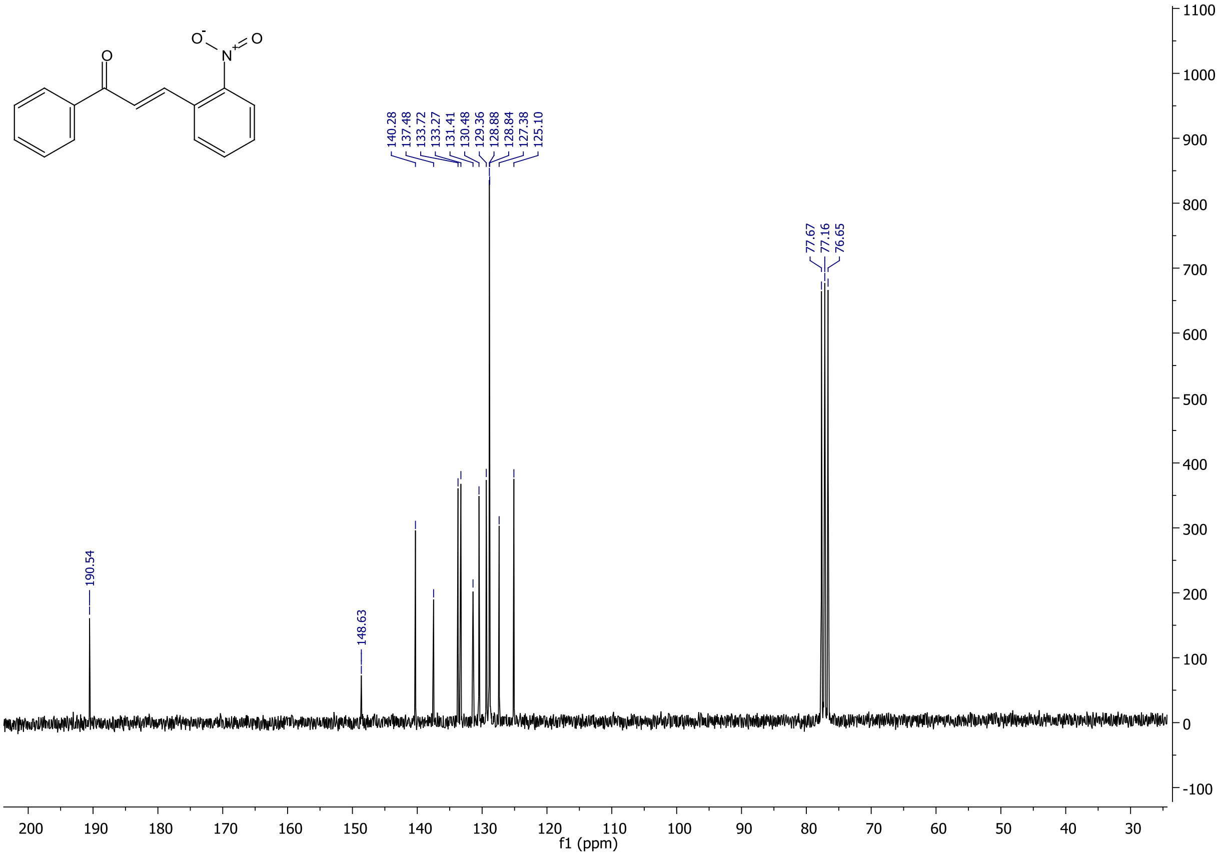

Figure S7. (*E*)-3-(3-nitrophenyl)-1-phenyl-2-propen-1-one **3h**.

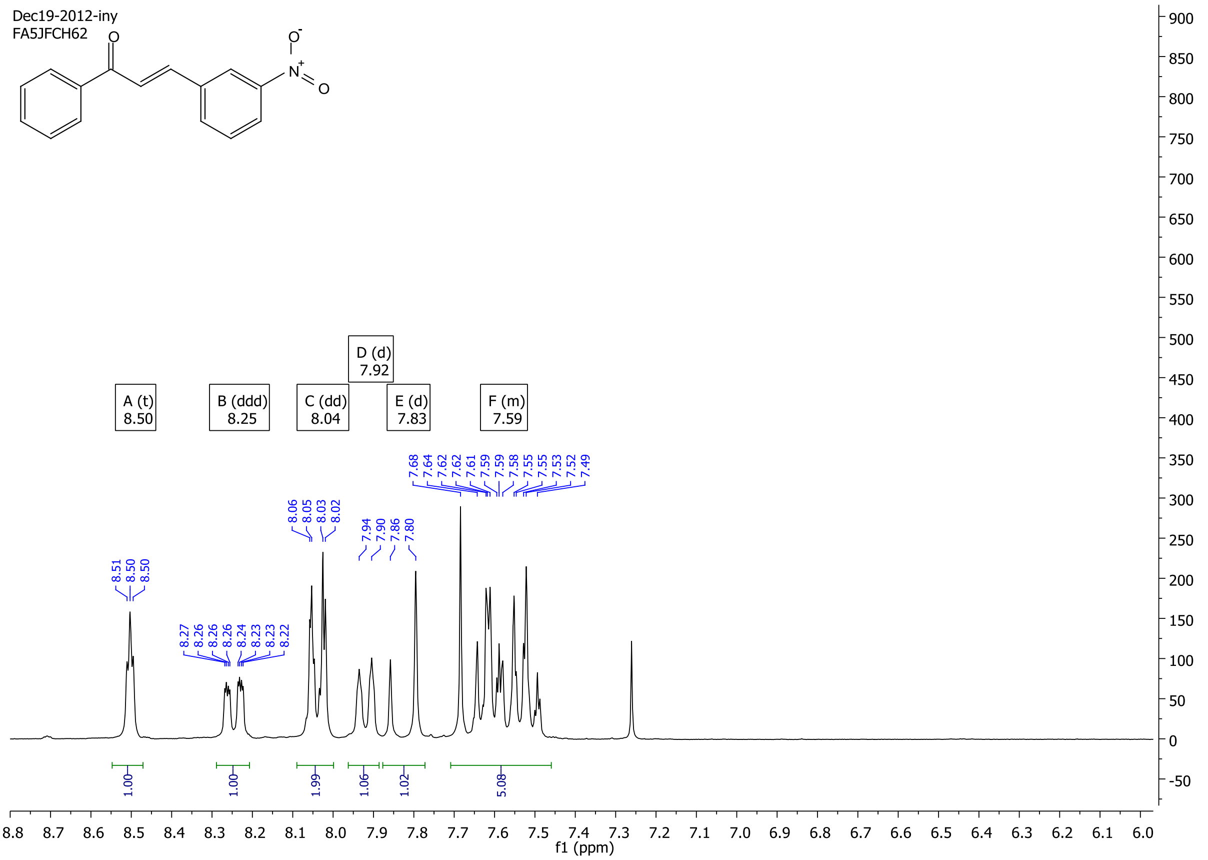

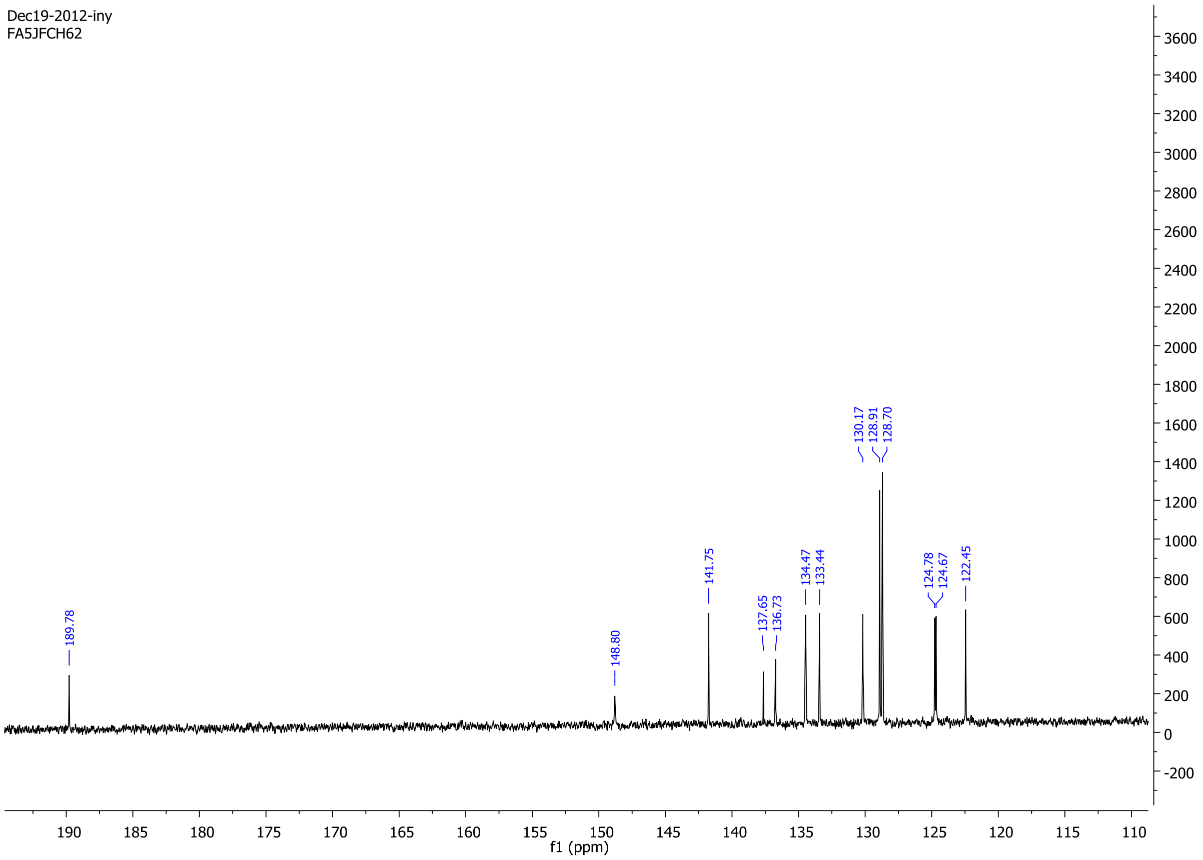

Figure S8. (*E*)-3-(4-nitrophenyl)-1-phenyl-2-propen-1-one **3i**.

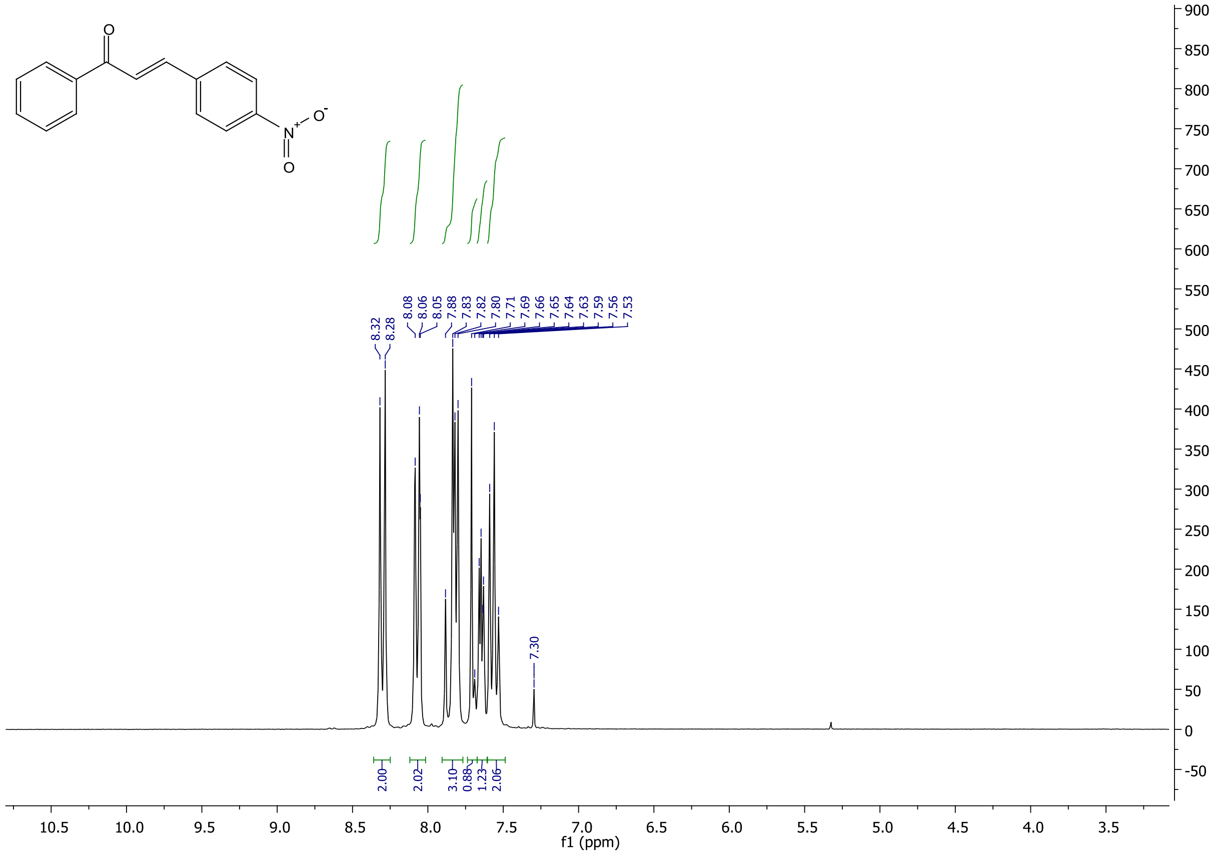

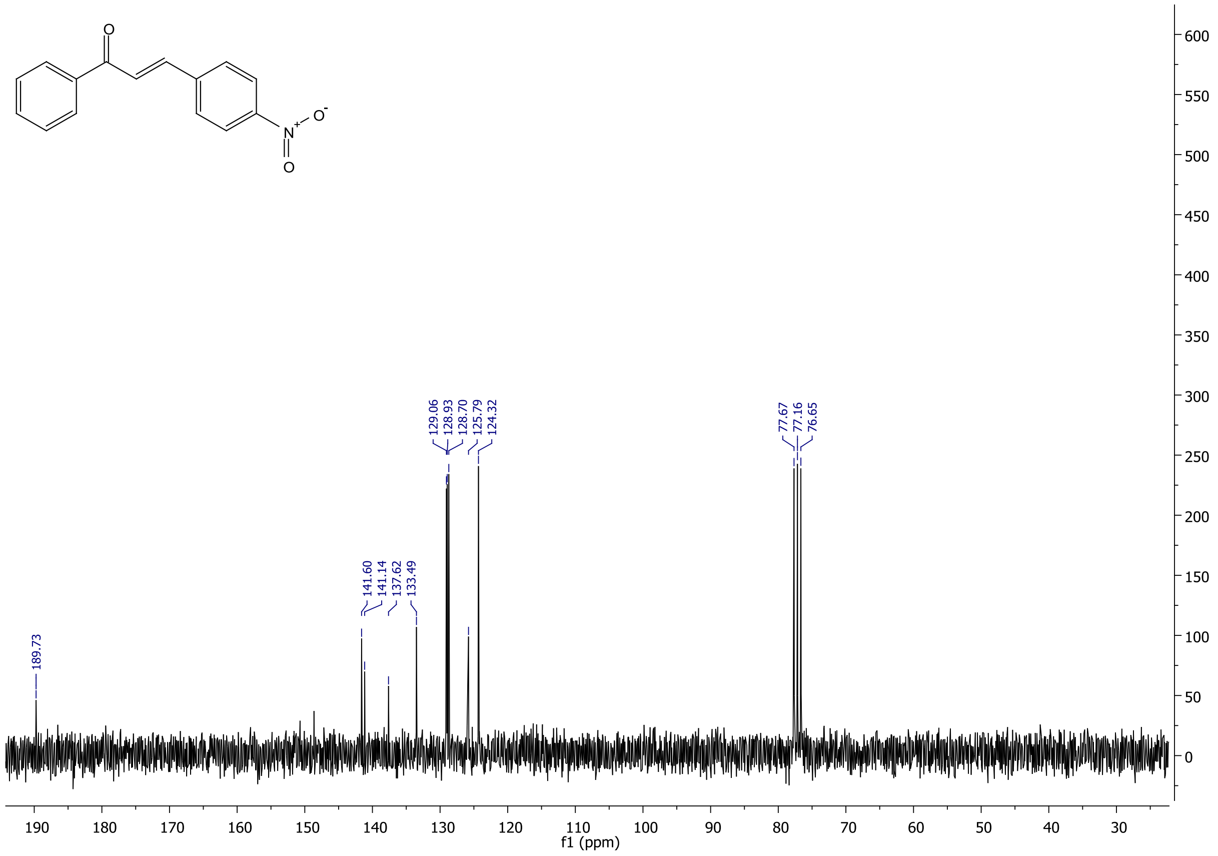

Figure S9. (*E*)-1-(4-methoxyphenyl)-3-phenyl-2-propen-1-one **3j**.

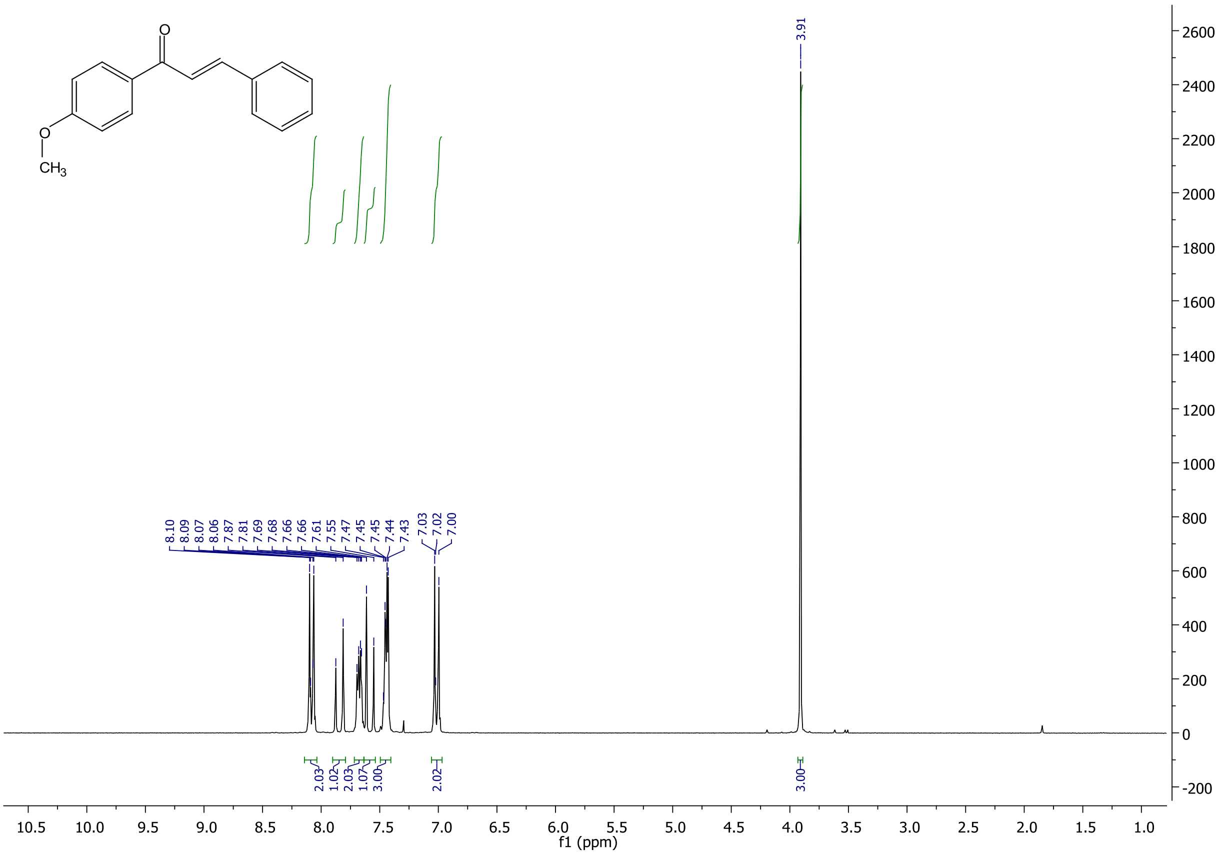

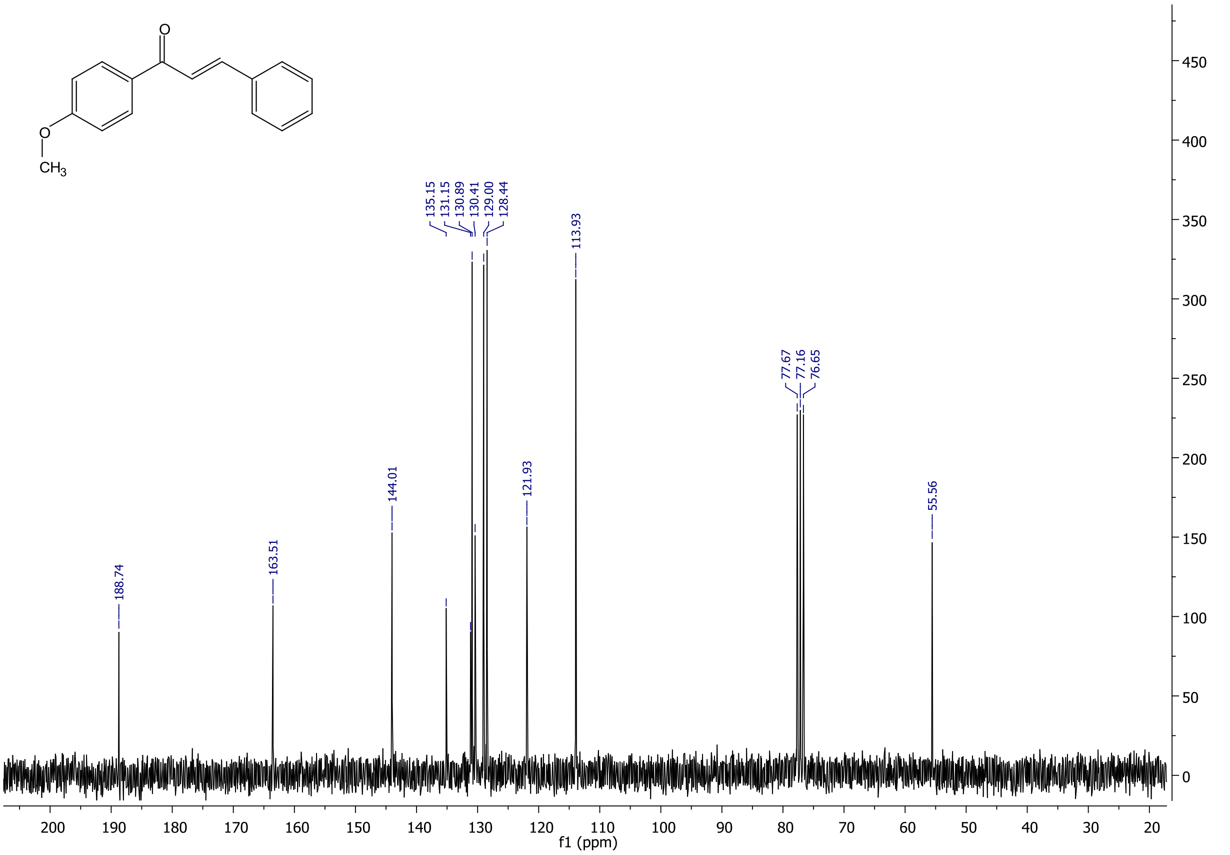

Figure S10. (*E*)-1,3-bis(4-methoxyphenyl)-2-propen-1-one **3k**.

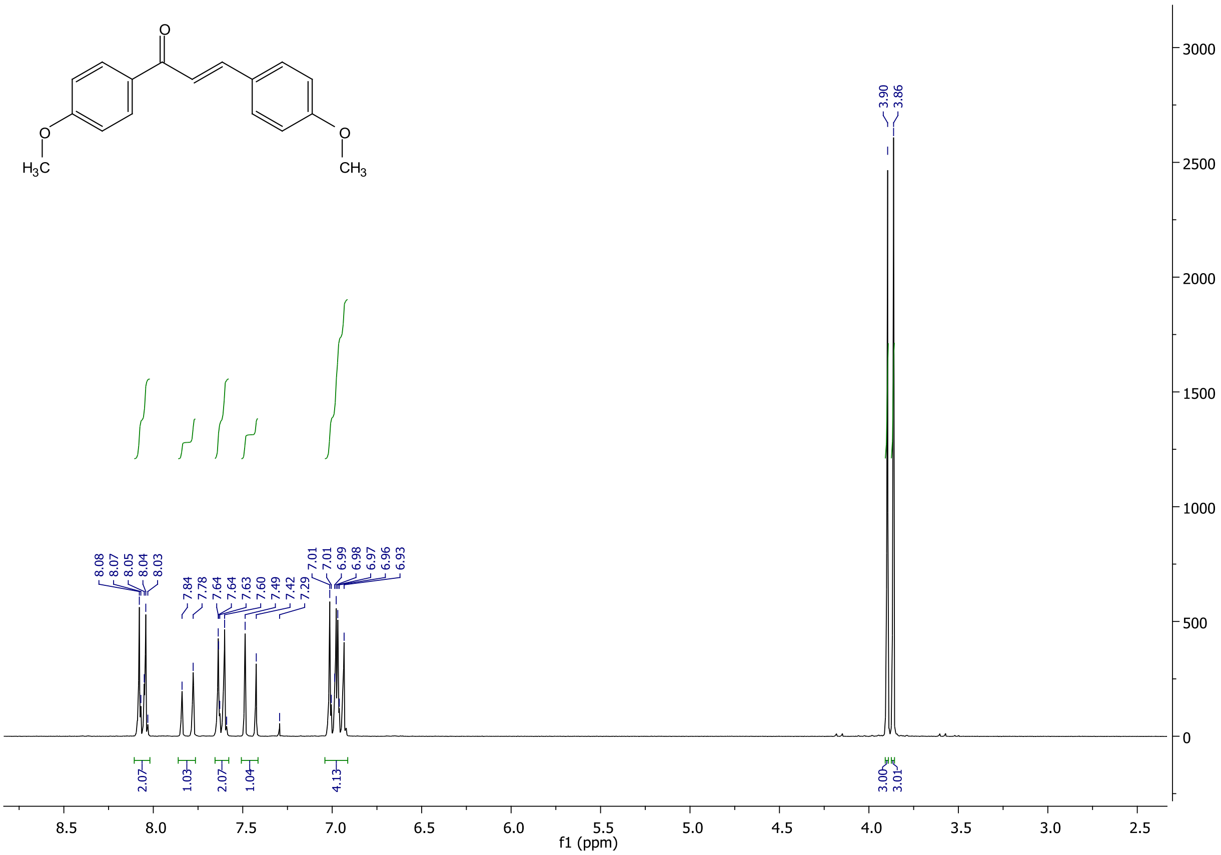

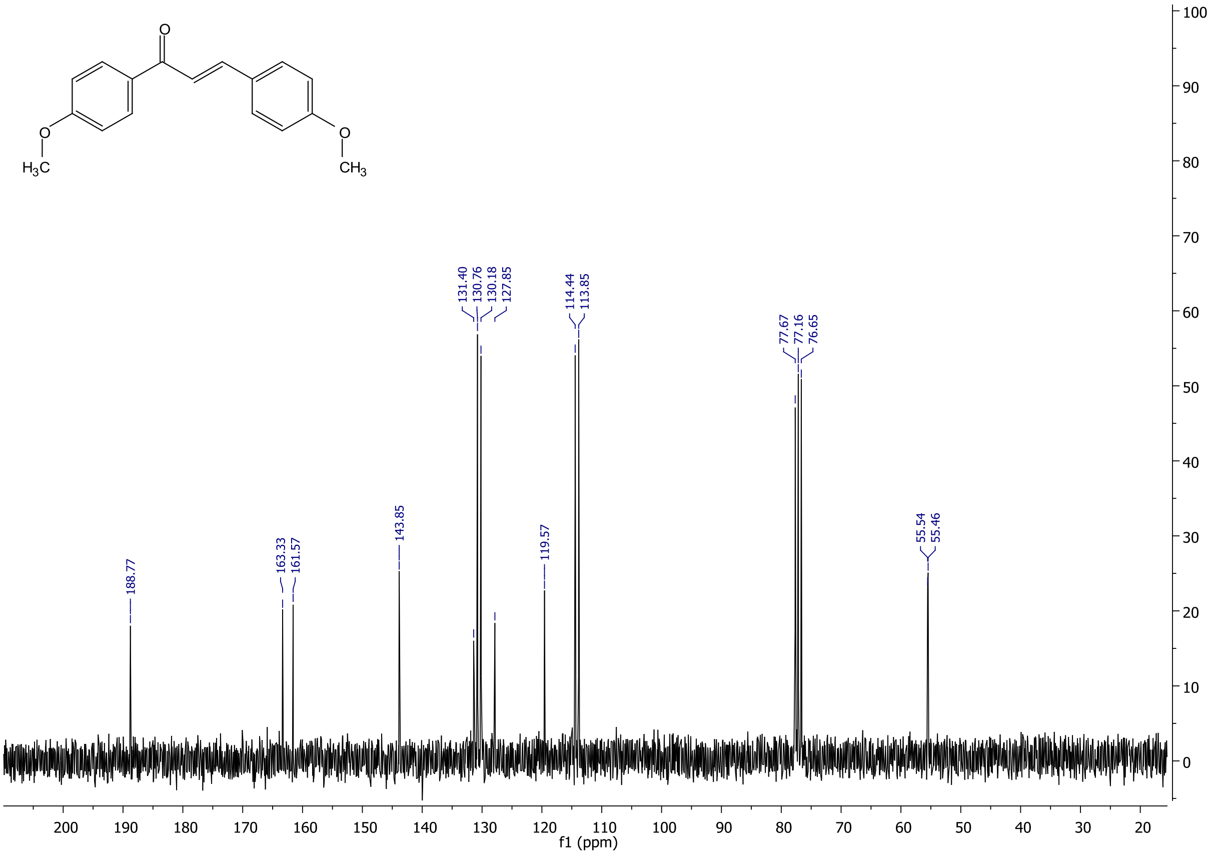

Figure S11. (*E*)-3-(4-bromophenyl)-1-(4-methoxyphenyl)-2-propen-1-one **3l**.

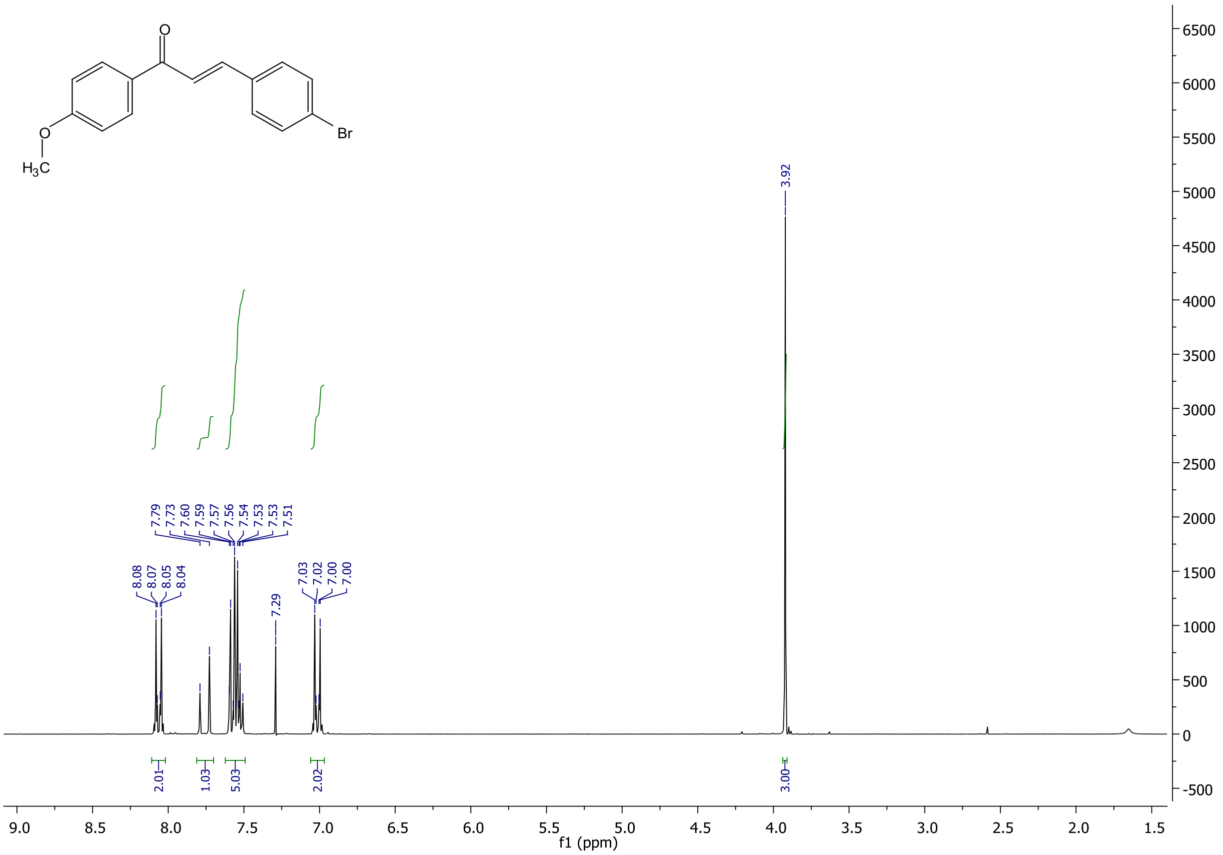

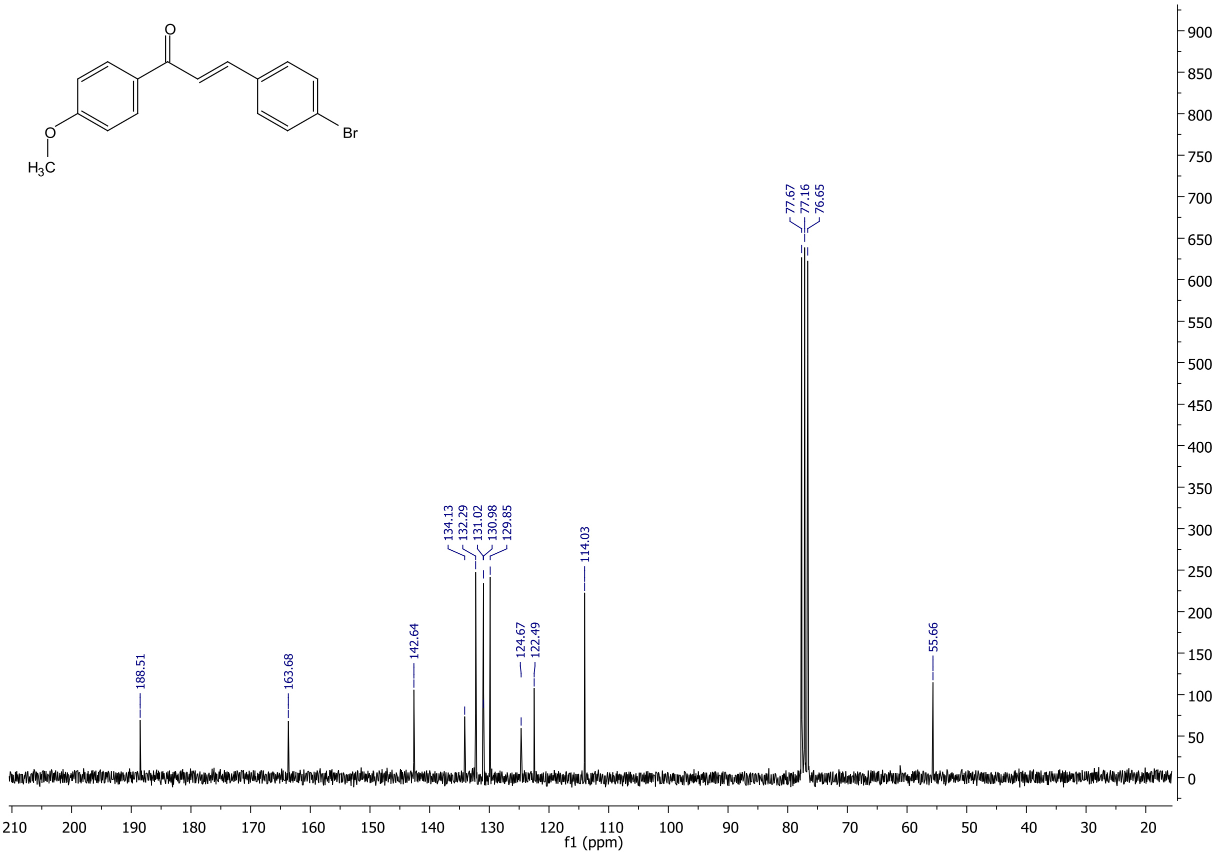

Figure S12. (*E*)-3-phenyl-1-(4-methilphenyl)-2-propen-1-one **3m**.

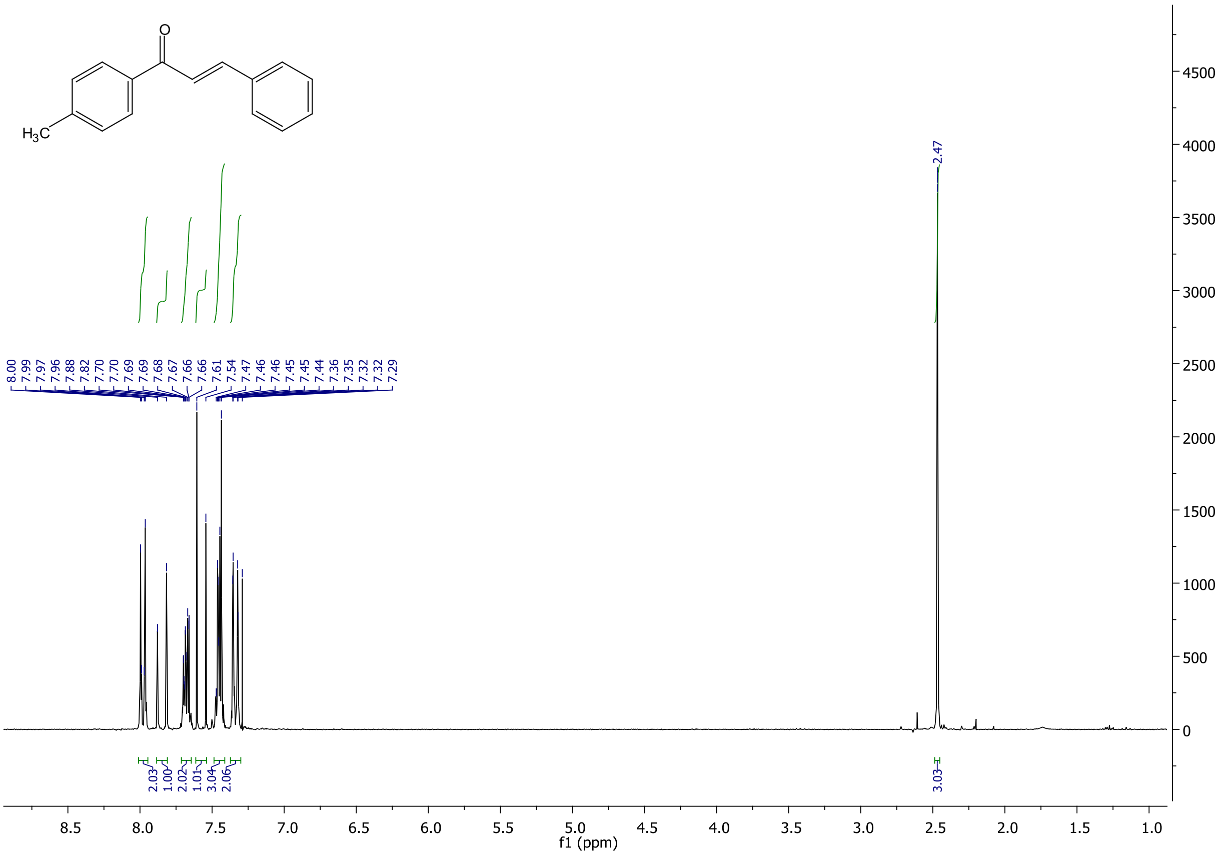

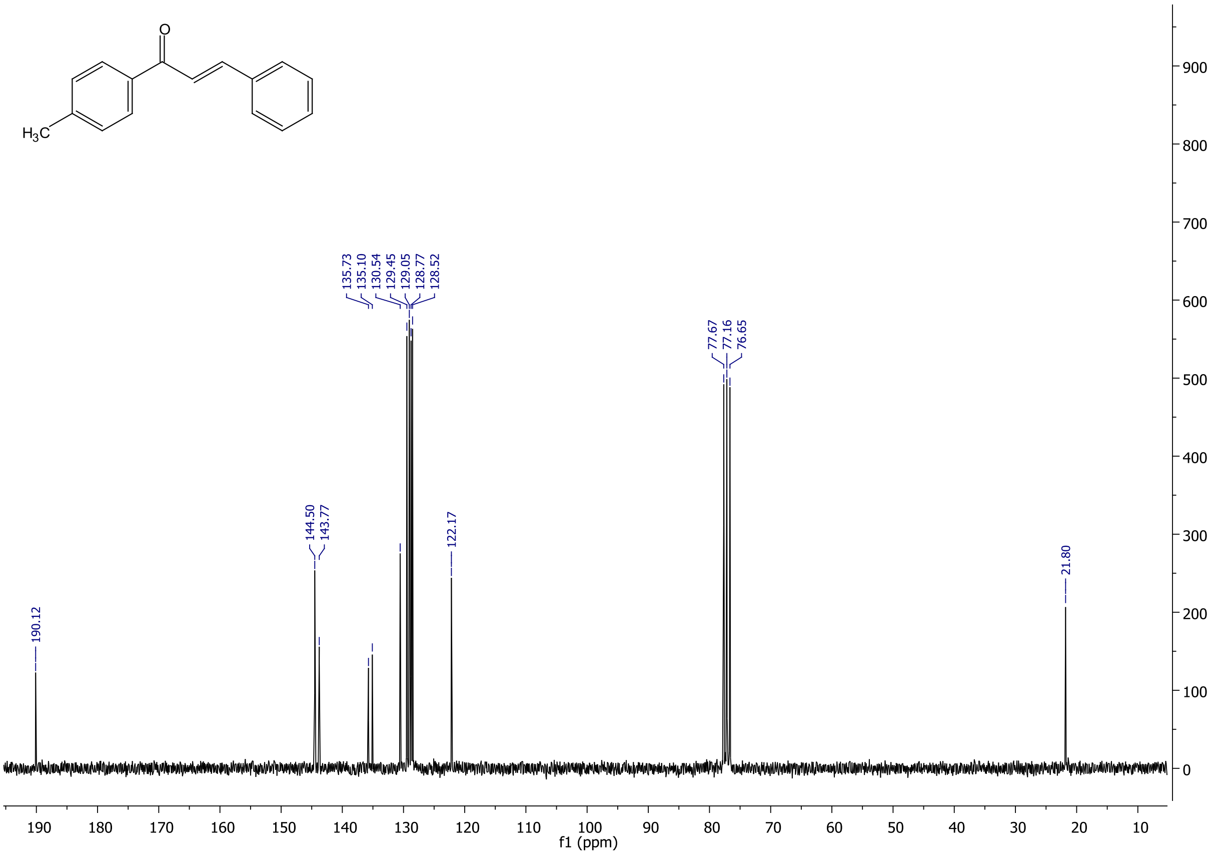

Figure S13. (*E*)-3-(4-Nitrophenyl)-1-(4-methoxyphenyl)-2-propen-1-one **3n**.

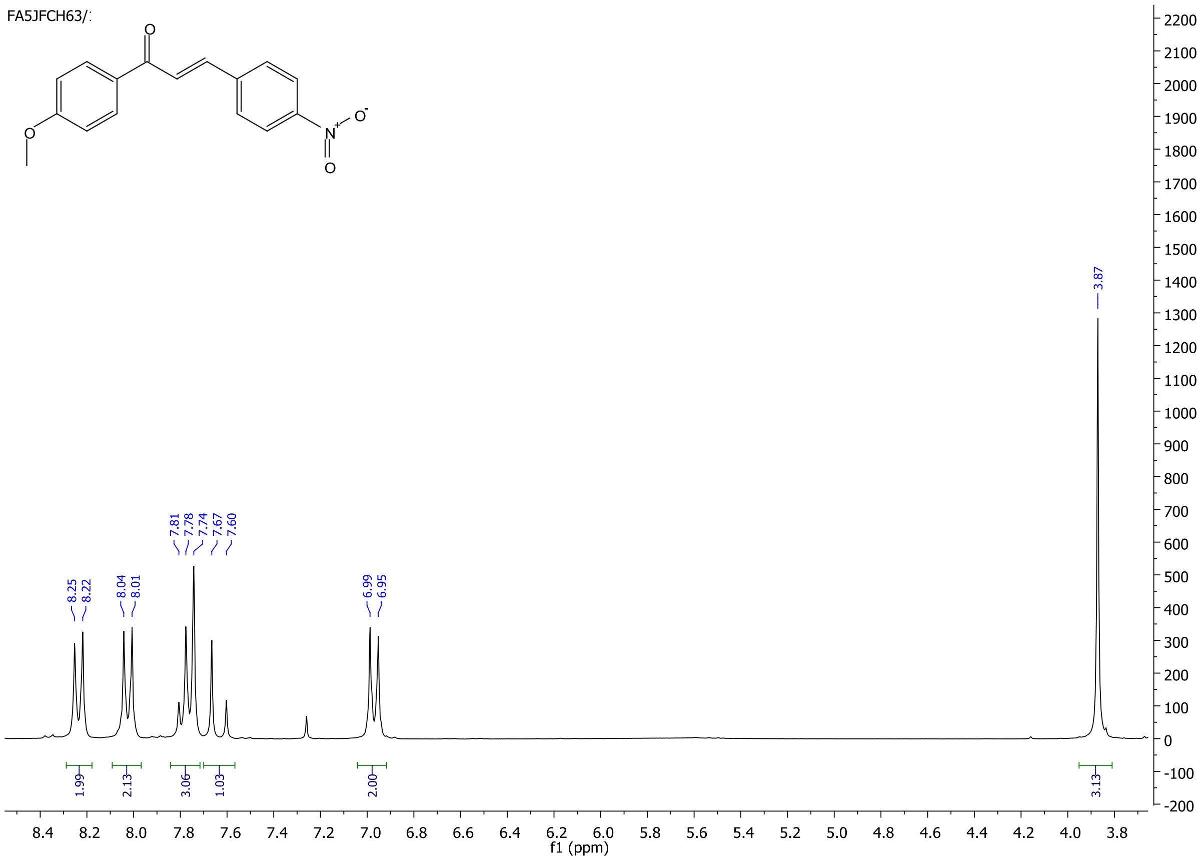

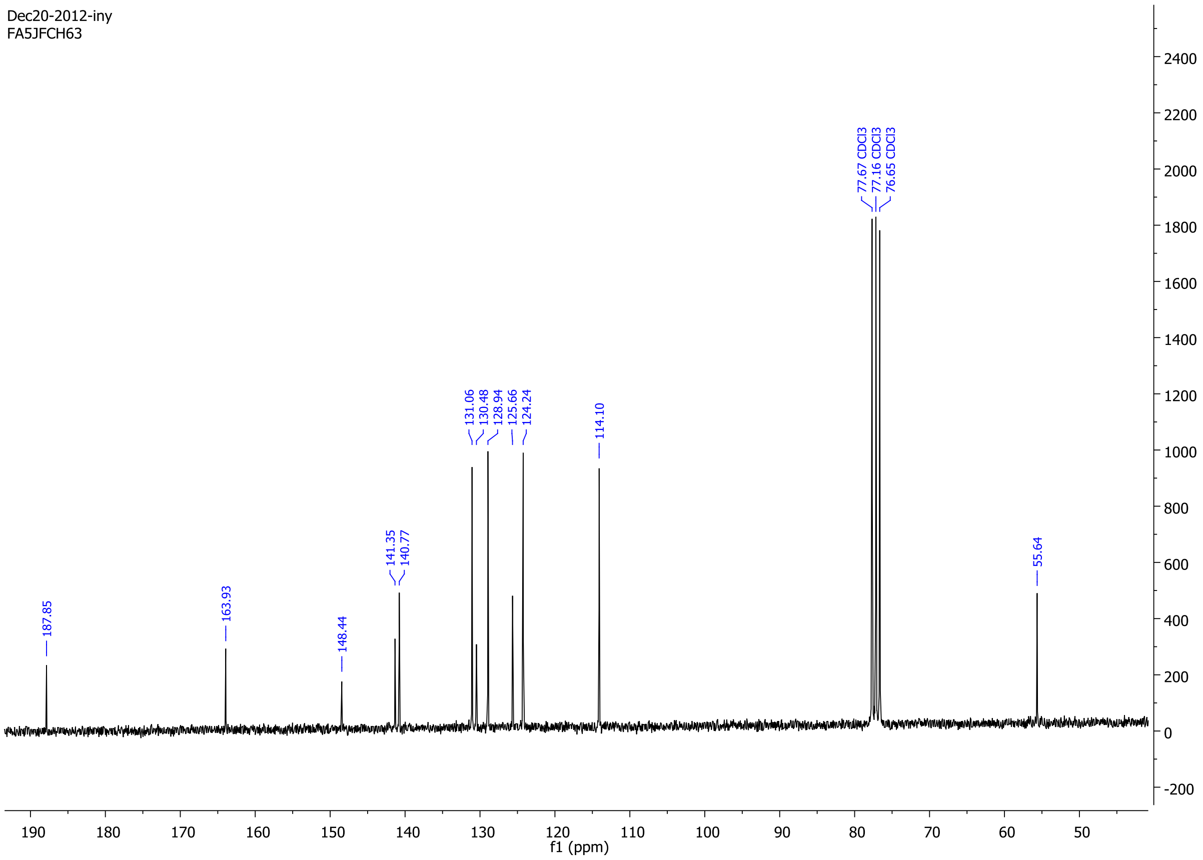

Figure S14. (*E*)-3-(2-methoxyphenyl)-1-(4-methilphenyl)-2-propen-1-one **3o**.

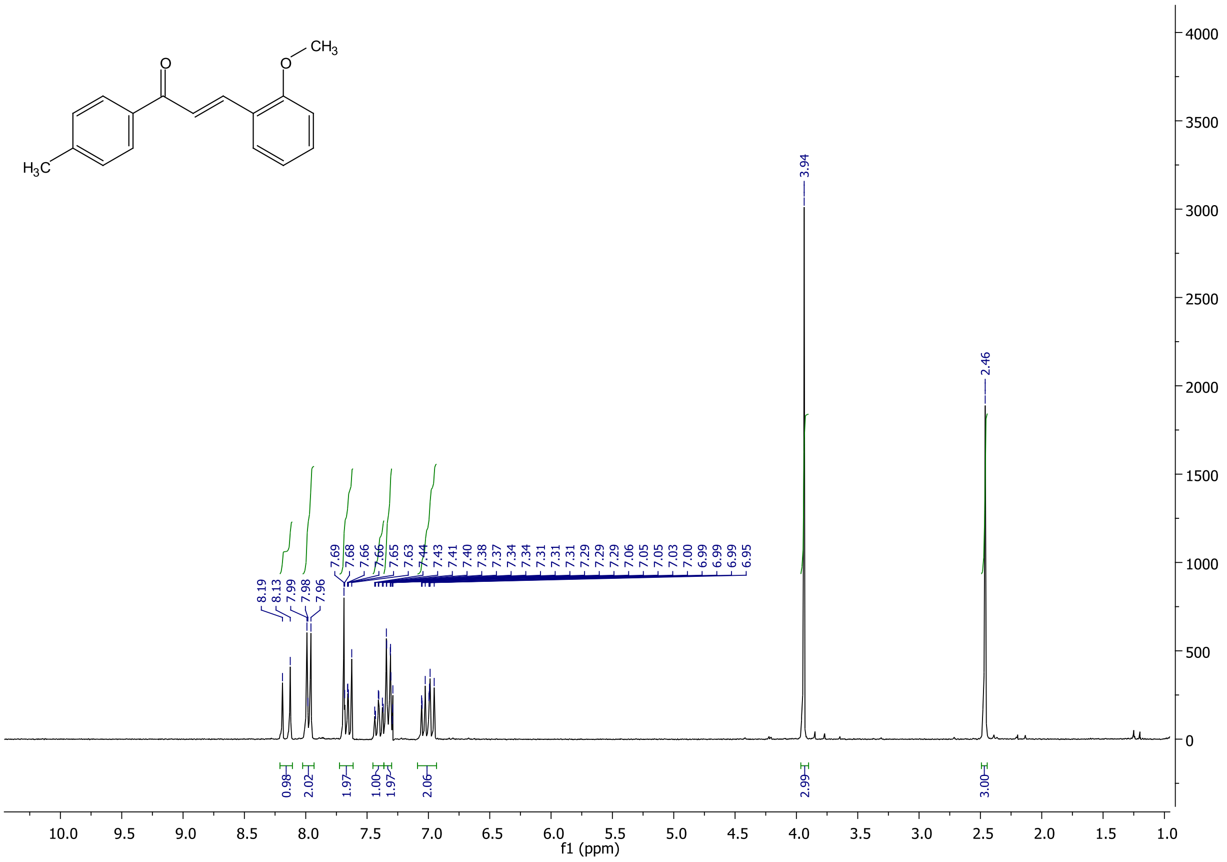

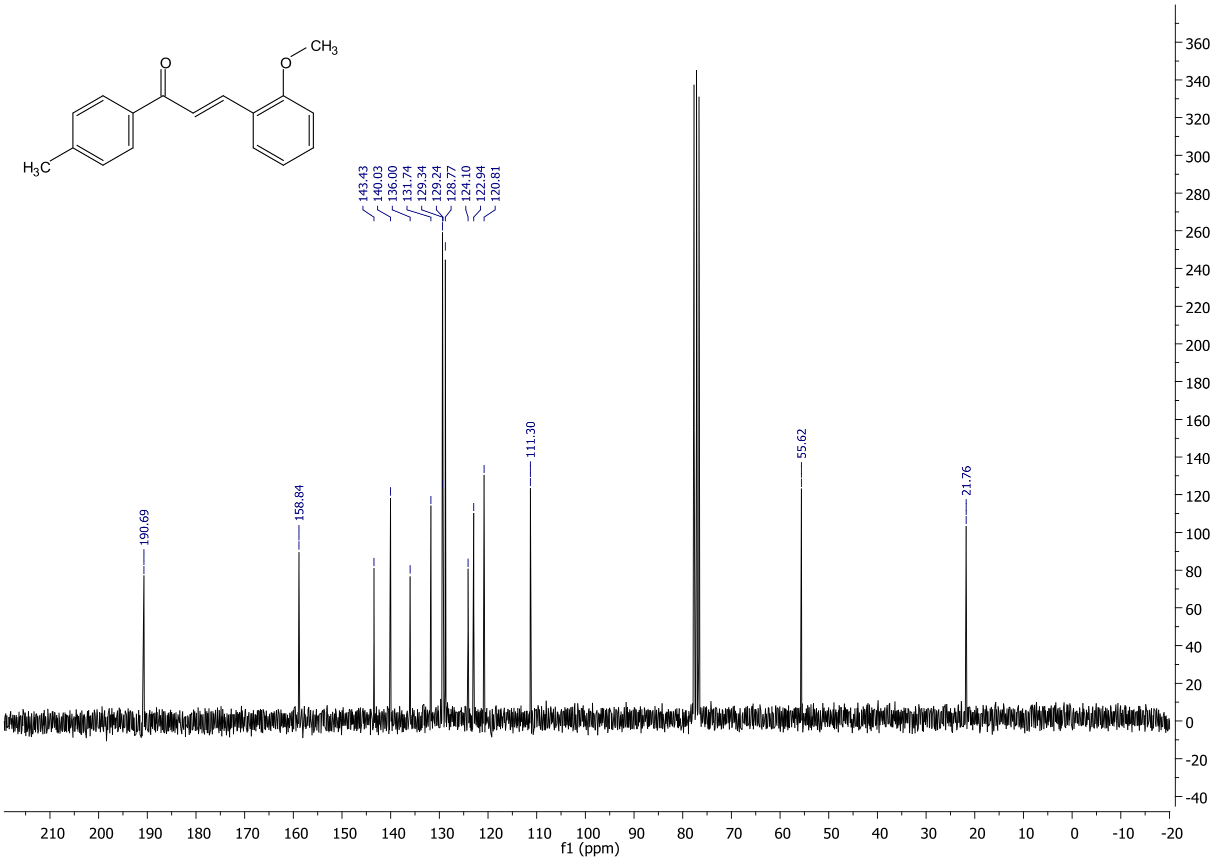

Figure S15. (*E*)-3-(4-methilphenyl)-1-(4-methilphenyl)-2-propen-1-one **3p**.

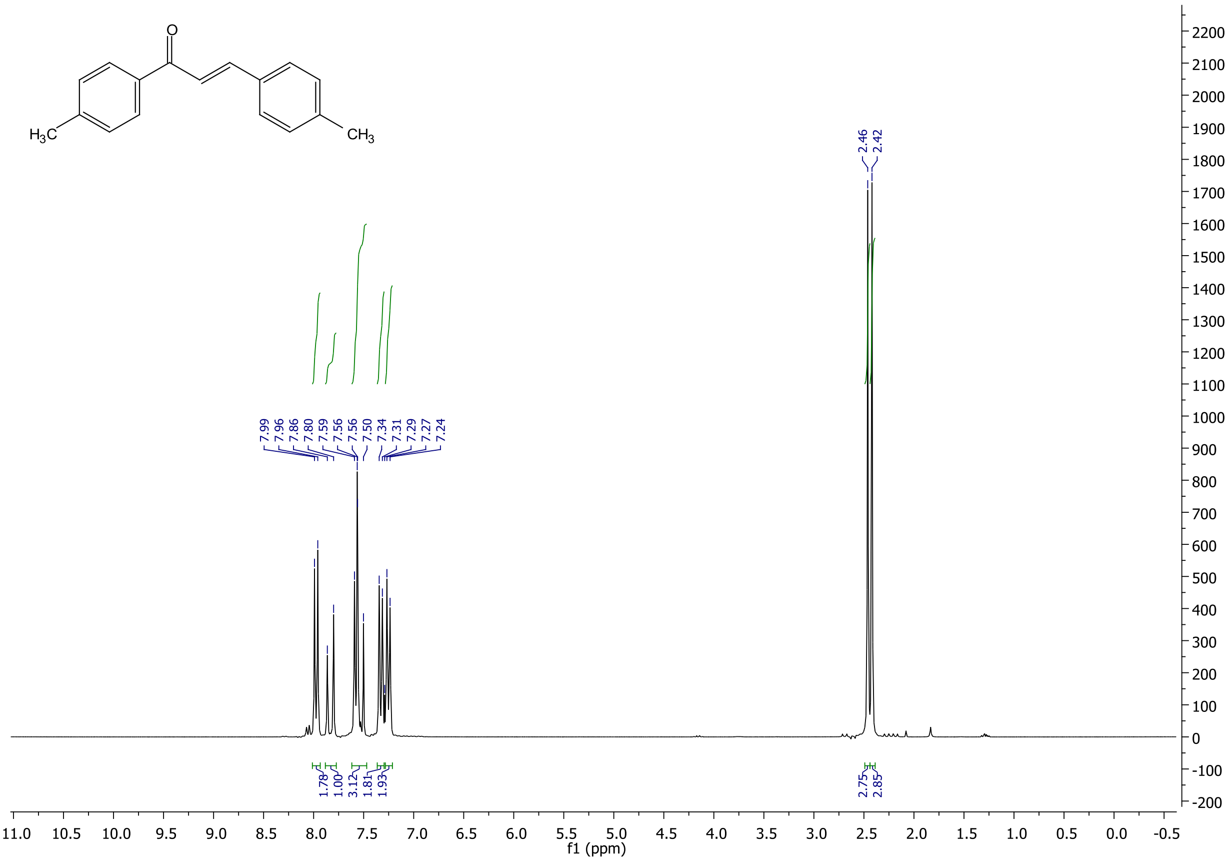

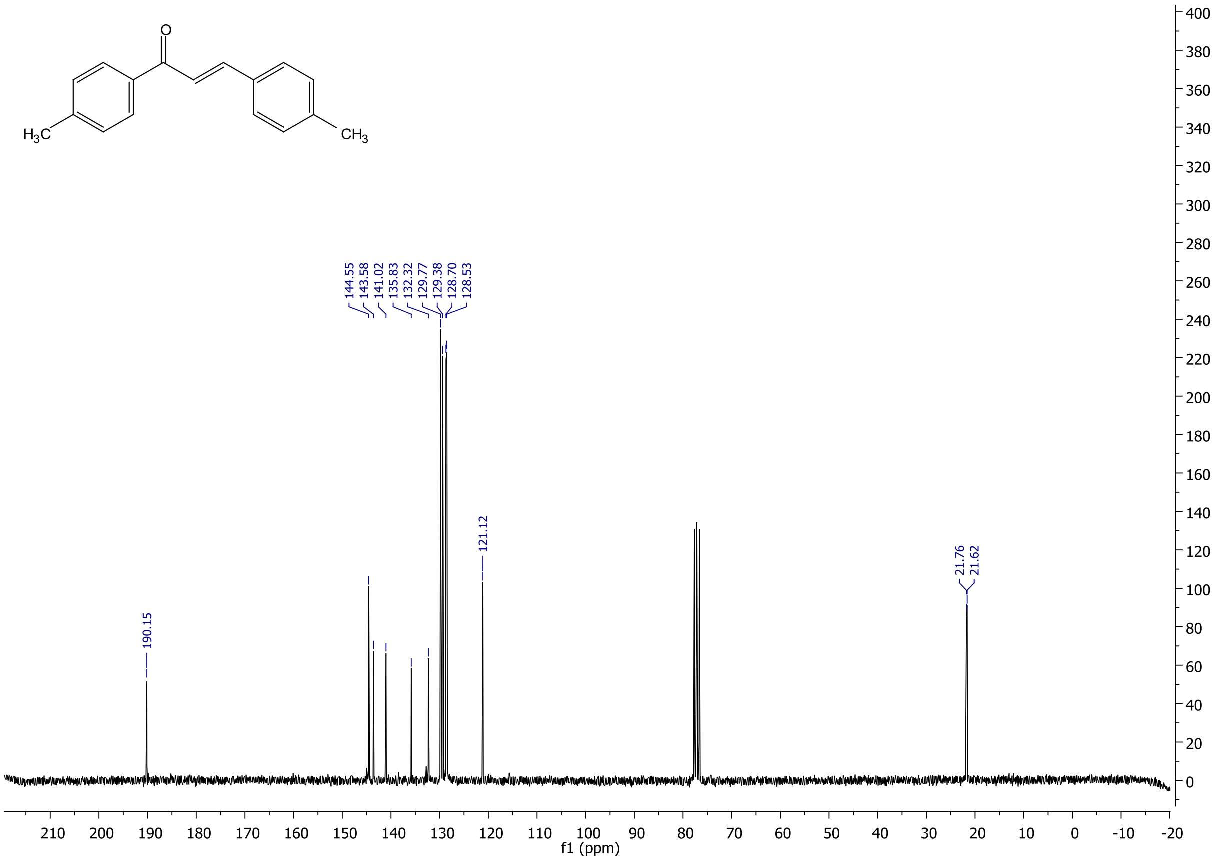

Figure S16. (*E*)-3-(4-bromophenyl)-1-(4-methilphenyl)-2-propen-1-one **3q**.

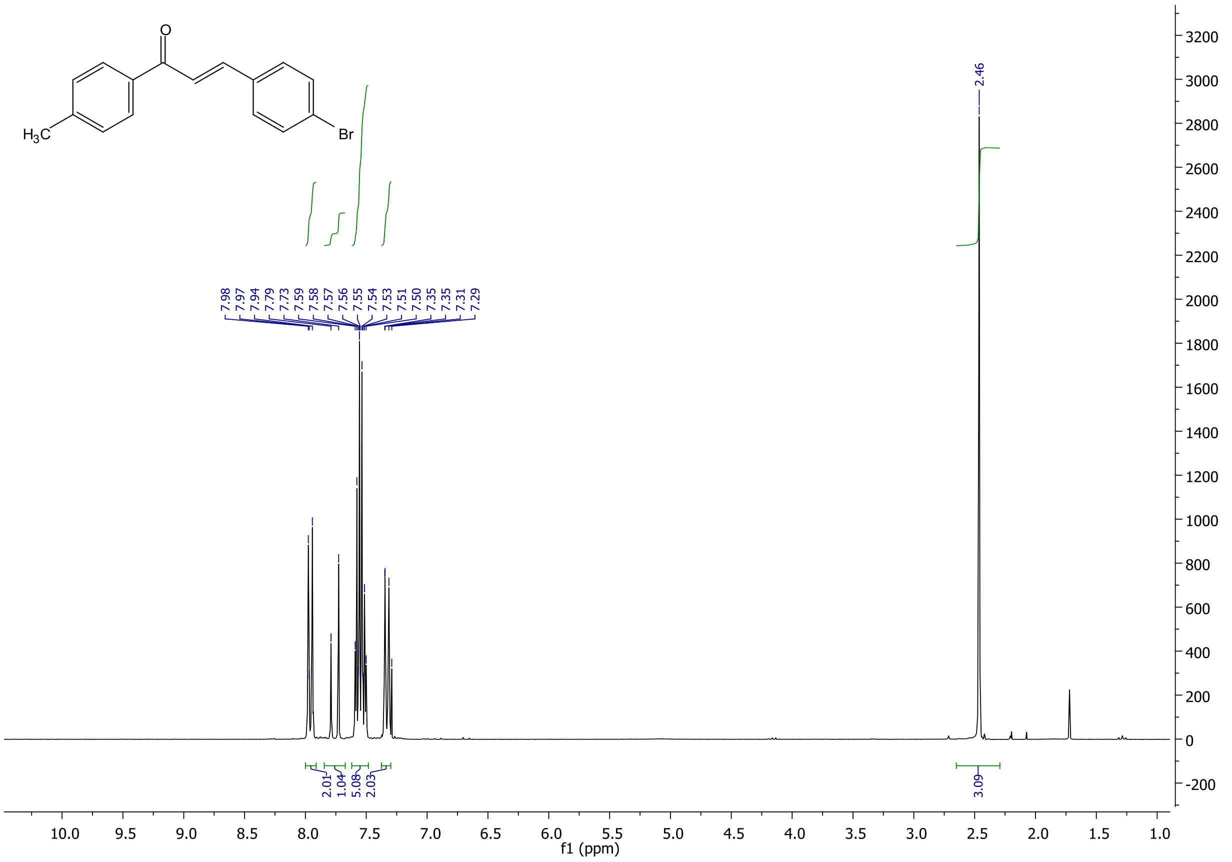

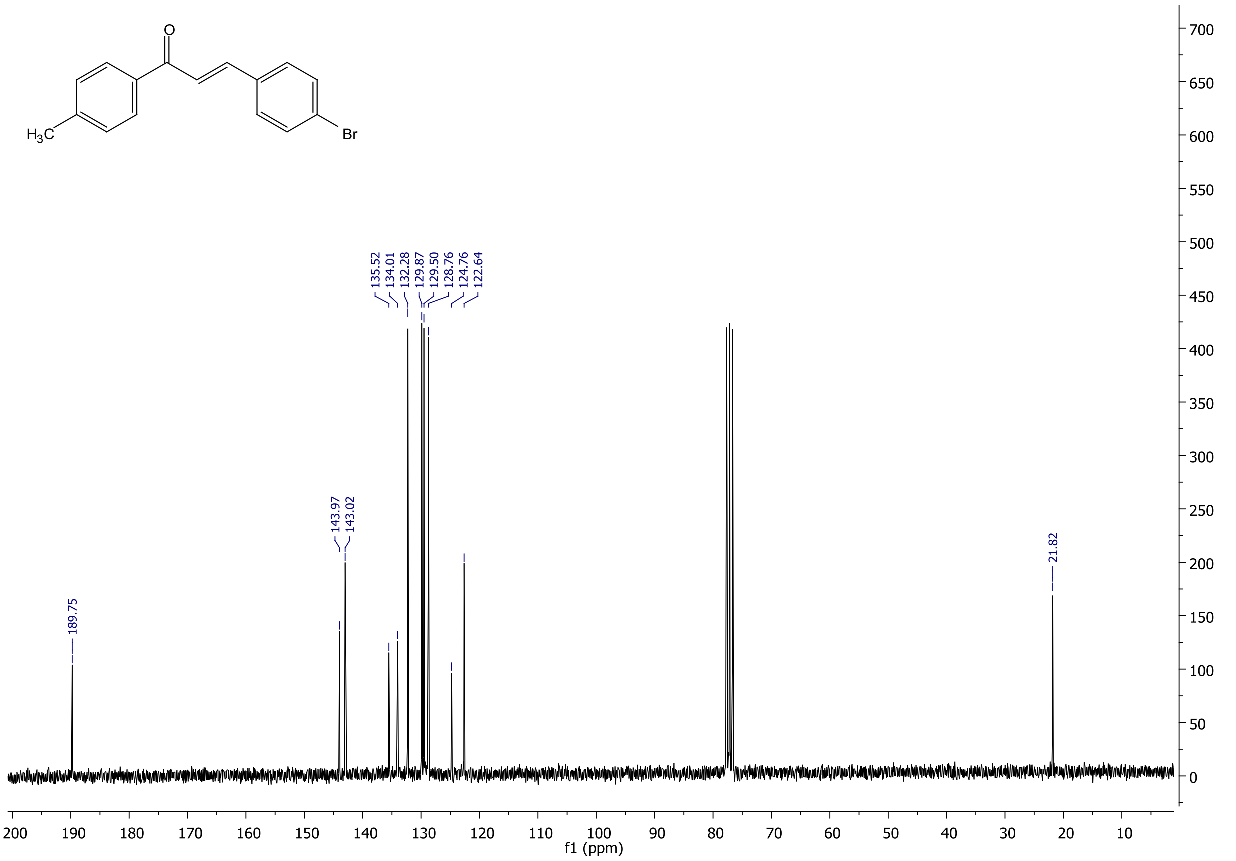

Figure S17. (*E*)-3-(2-nitrophenyl)-1-(4-methilphenyl)-2-propen-1-one **3r**.

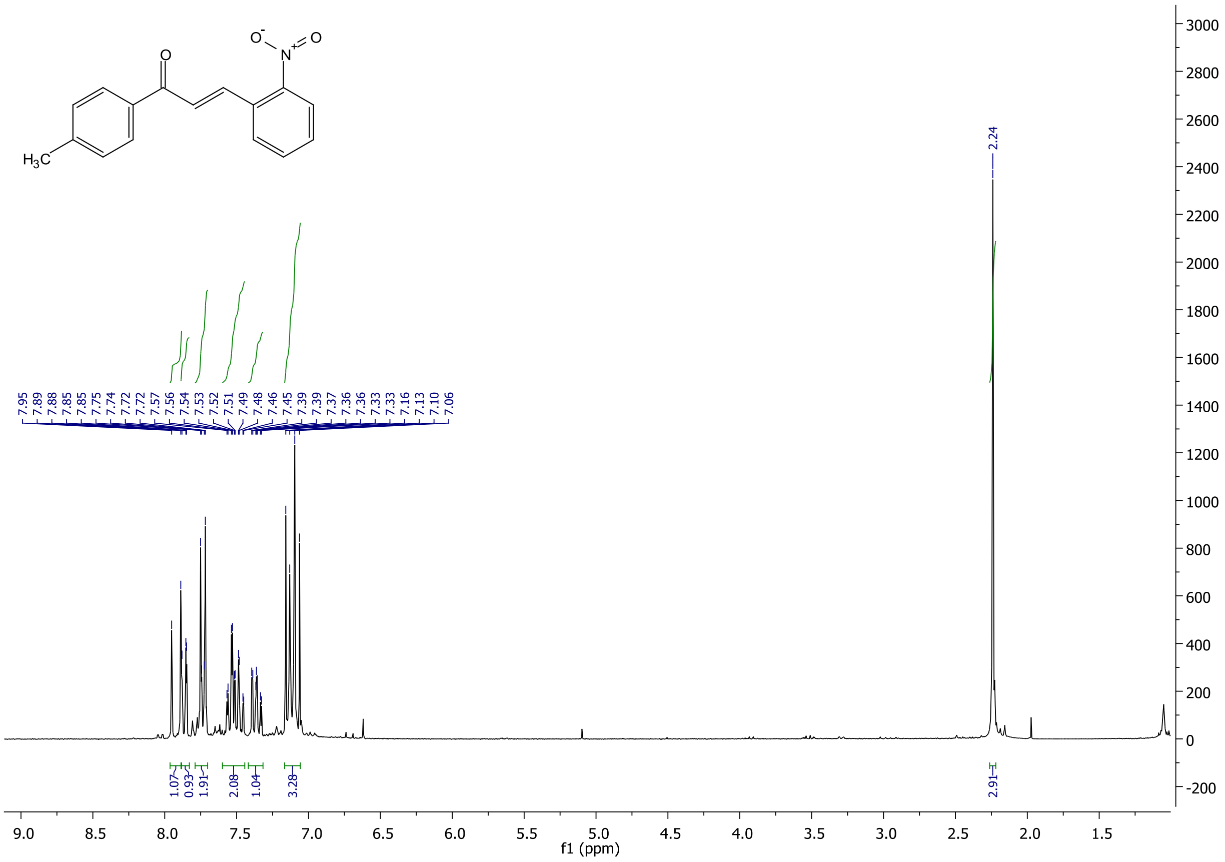

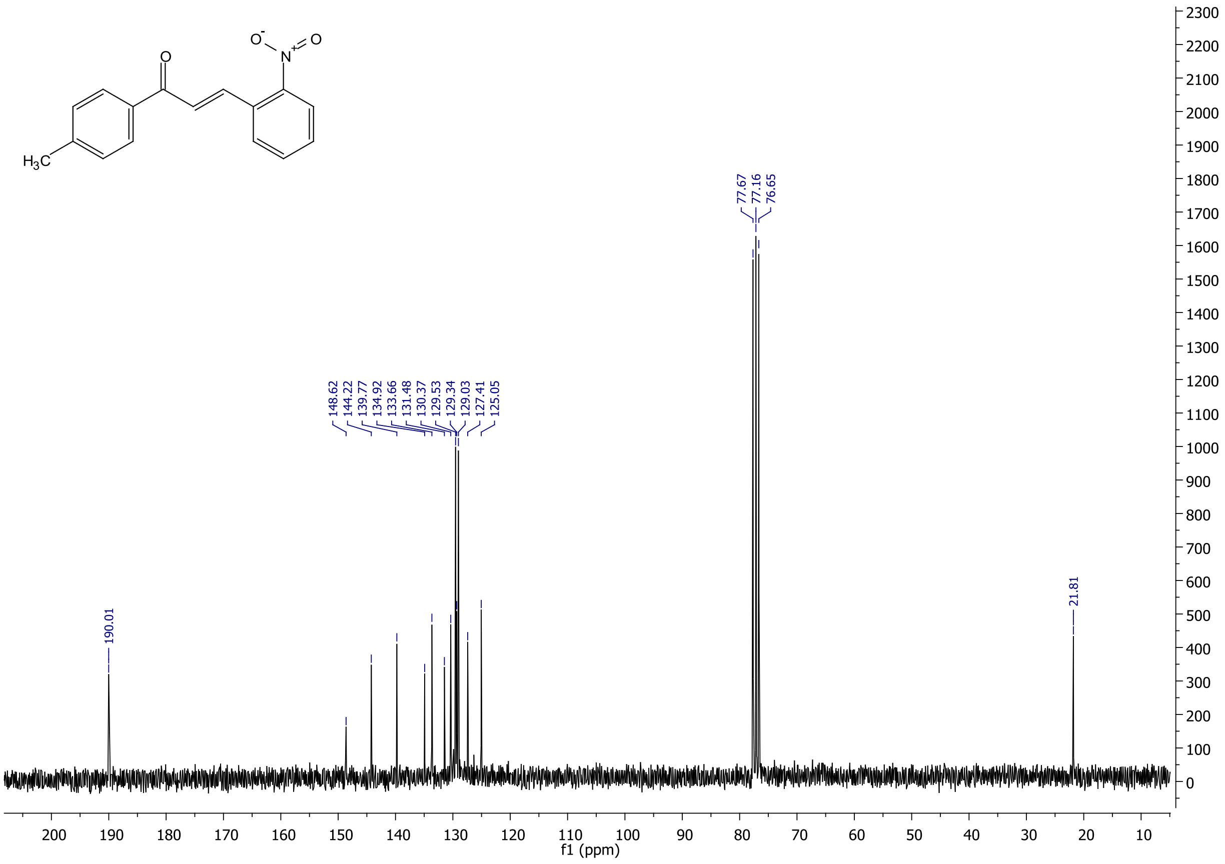

Figure S18. (*E*)-1-(4-chlorophenyl)-3-phenyl-2-propen-1-one **3s**.

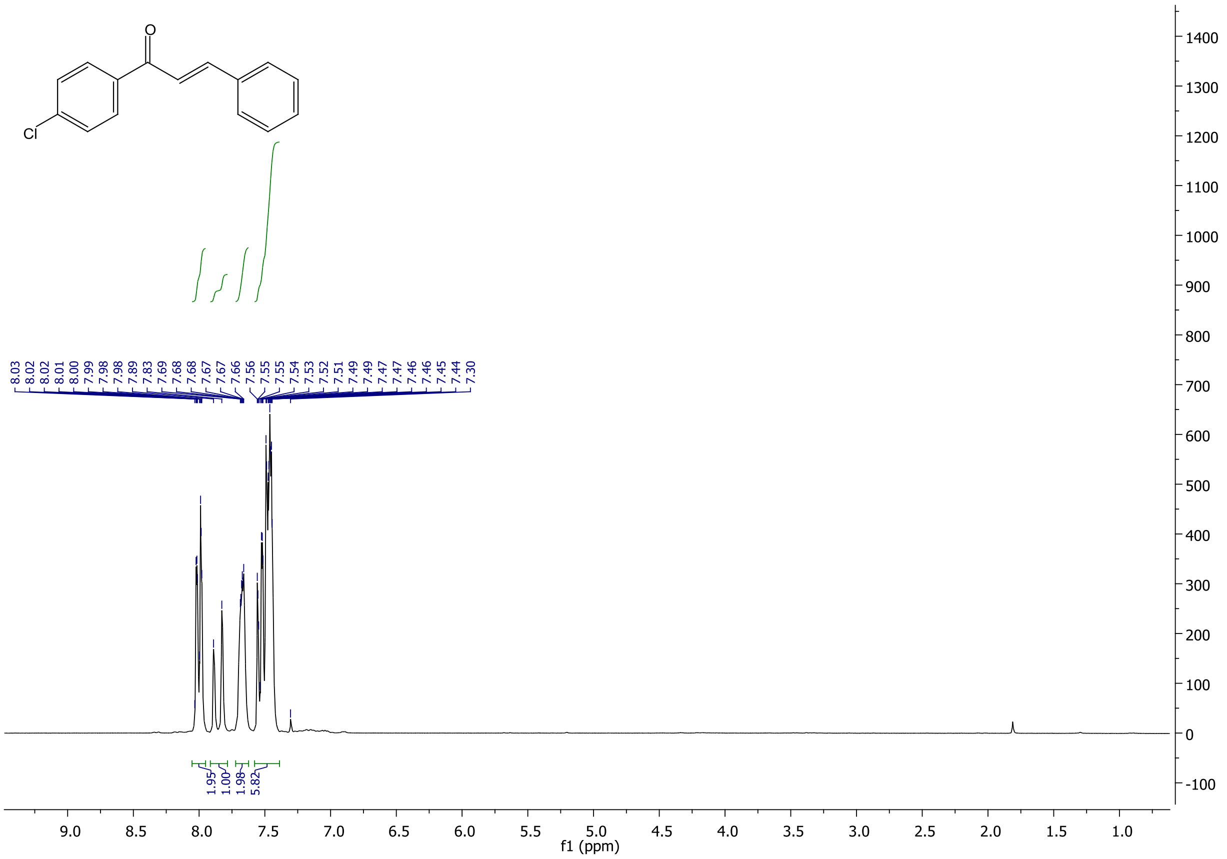

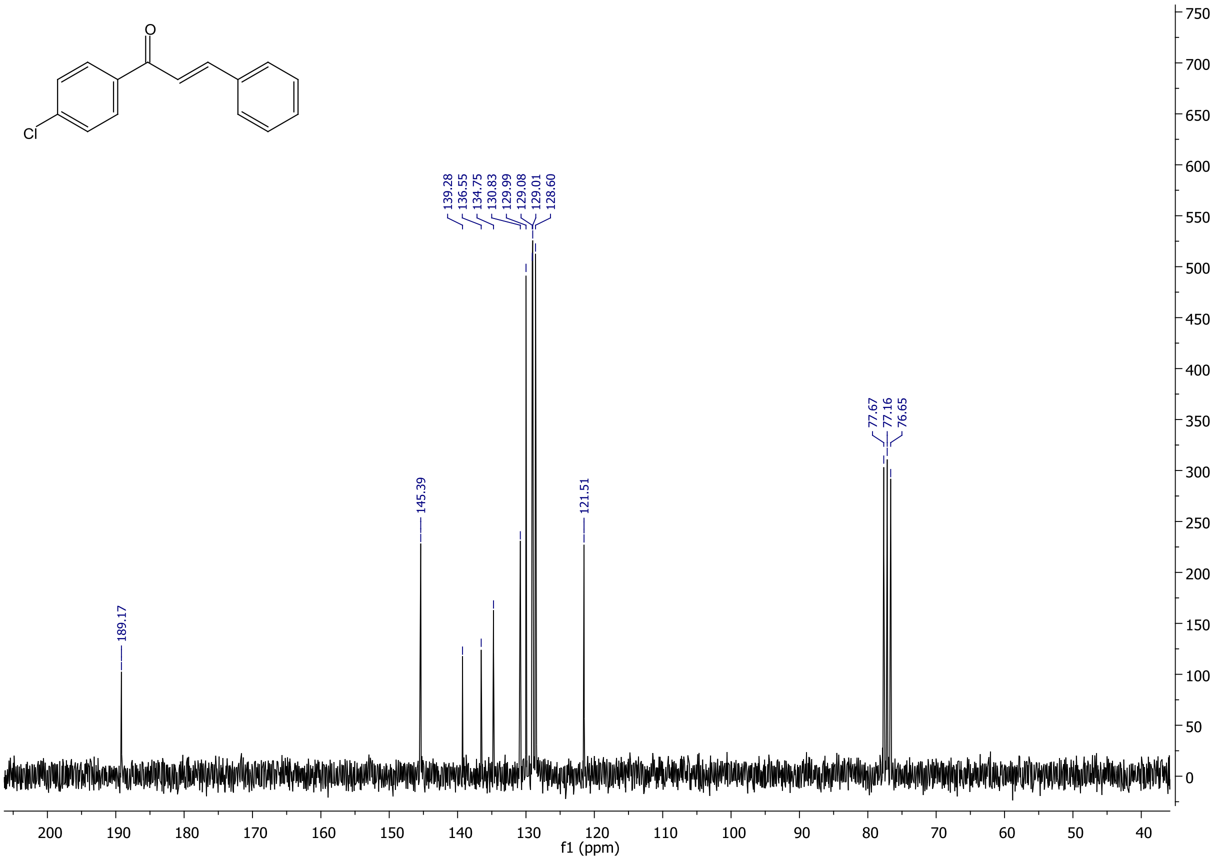

Figure S19. (*E*)-1-(4-chlorophenyl)-3-(3-methoxyphenyl)-2-propen-1-one **3t**.

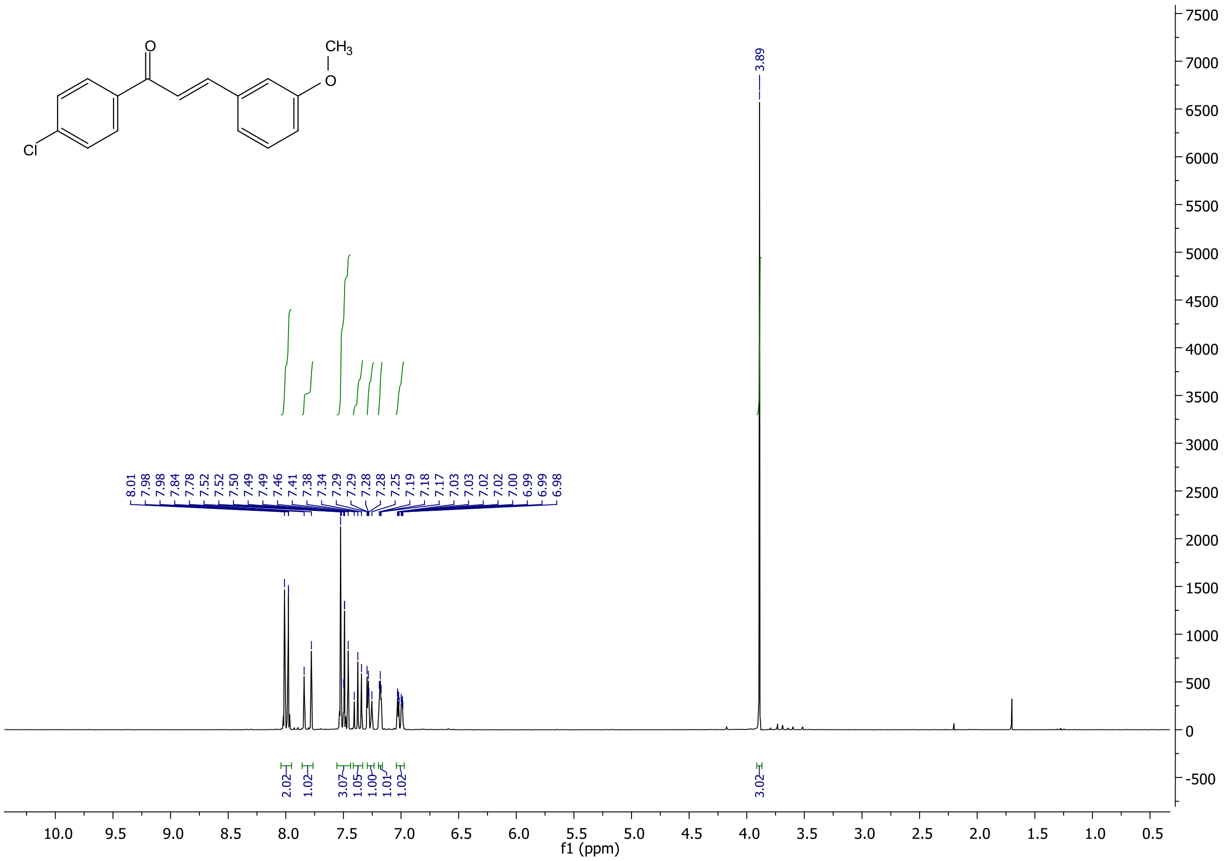

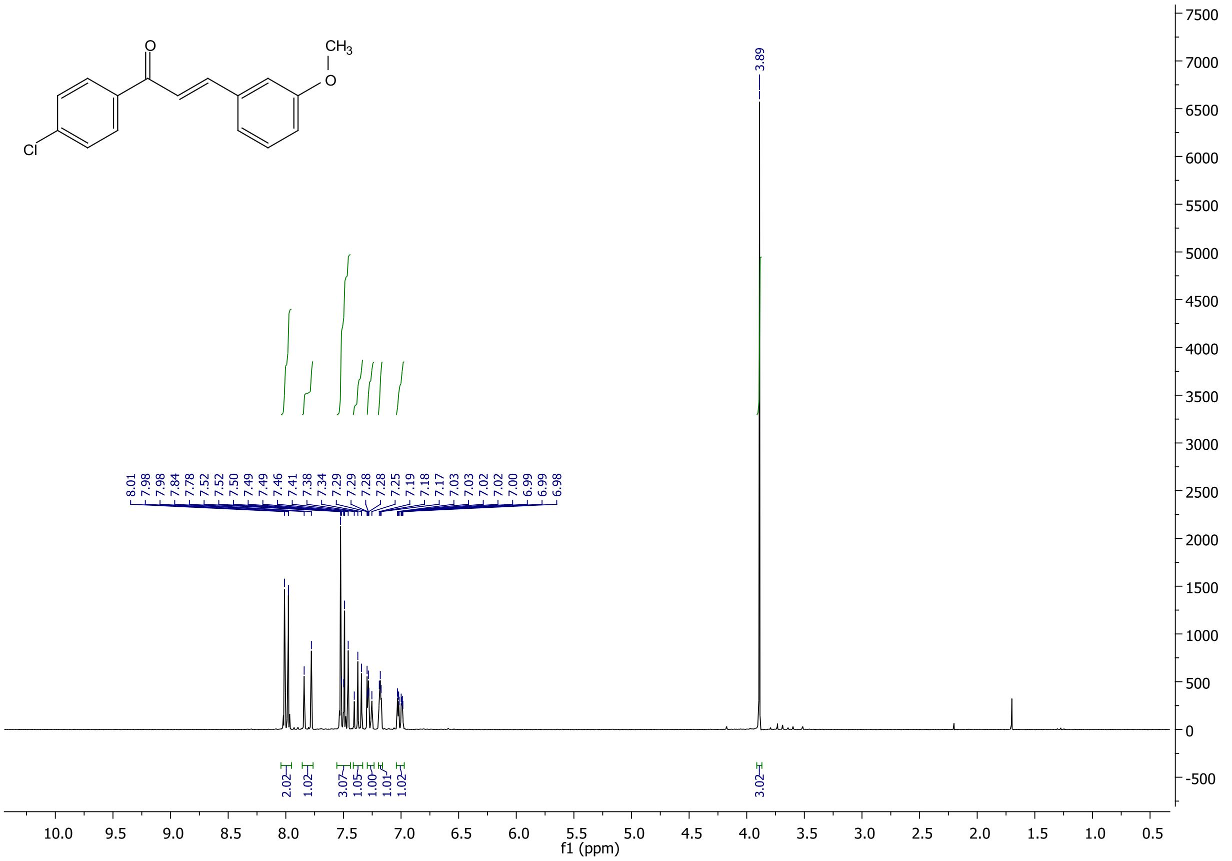

Figure S20. (*E*)-1-(4-chlorophenyl)-3-(4-bromophenyl)-2-propen-1-one **3u**.

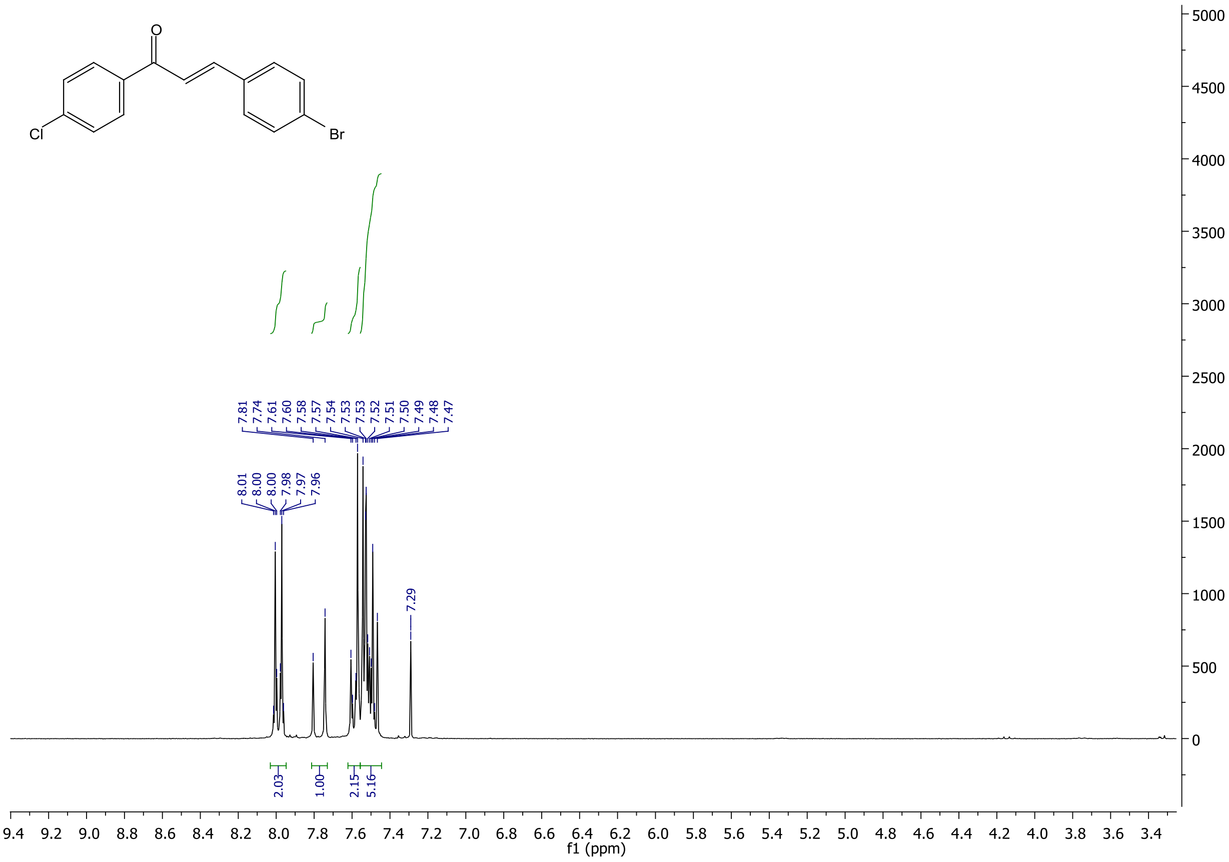

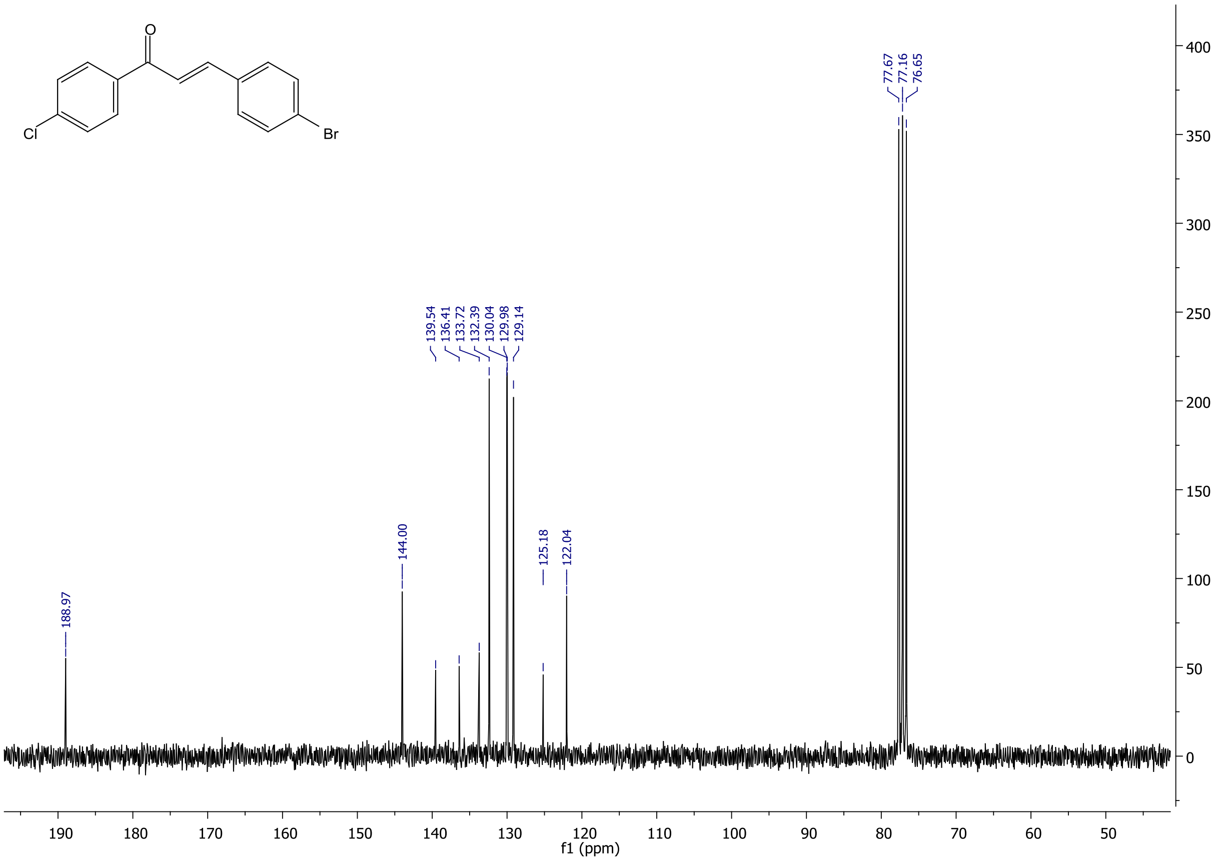

Figure S21. (*E*)-1-(4-chlorophenyl)-3-(2-nitrophenyl)-2-propen-1-one **3v**.

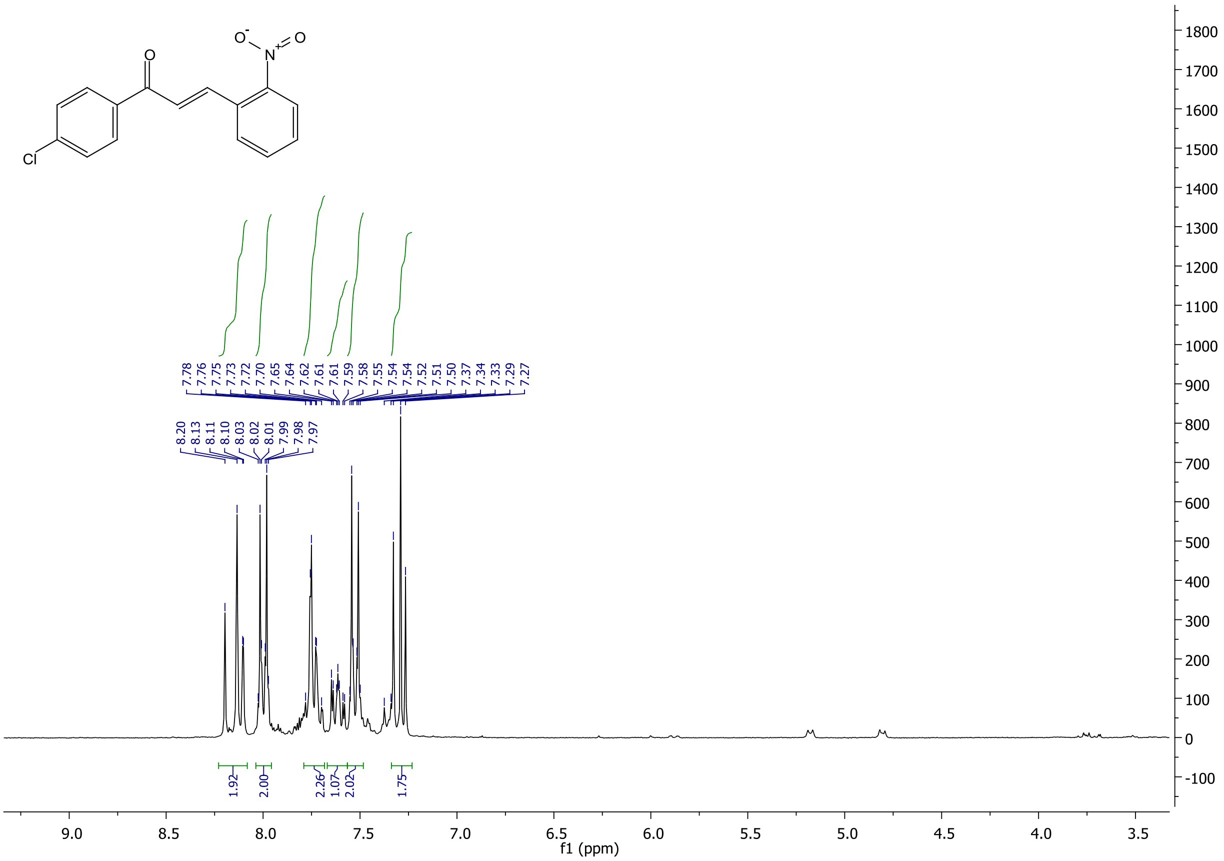

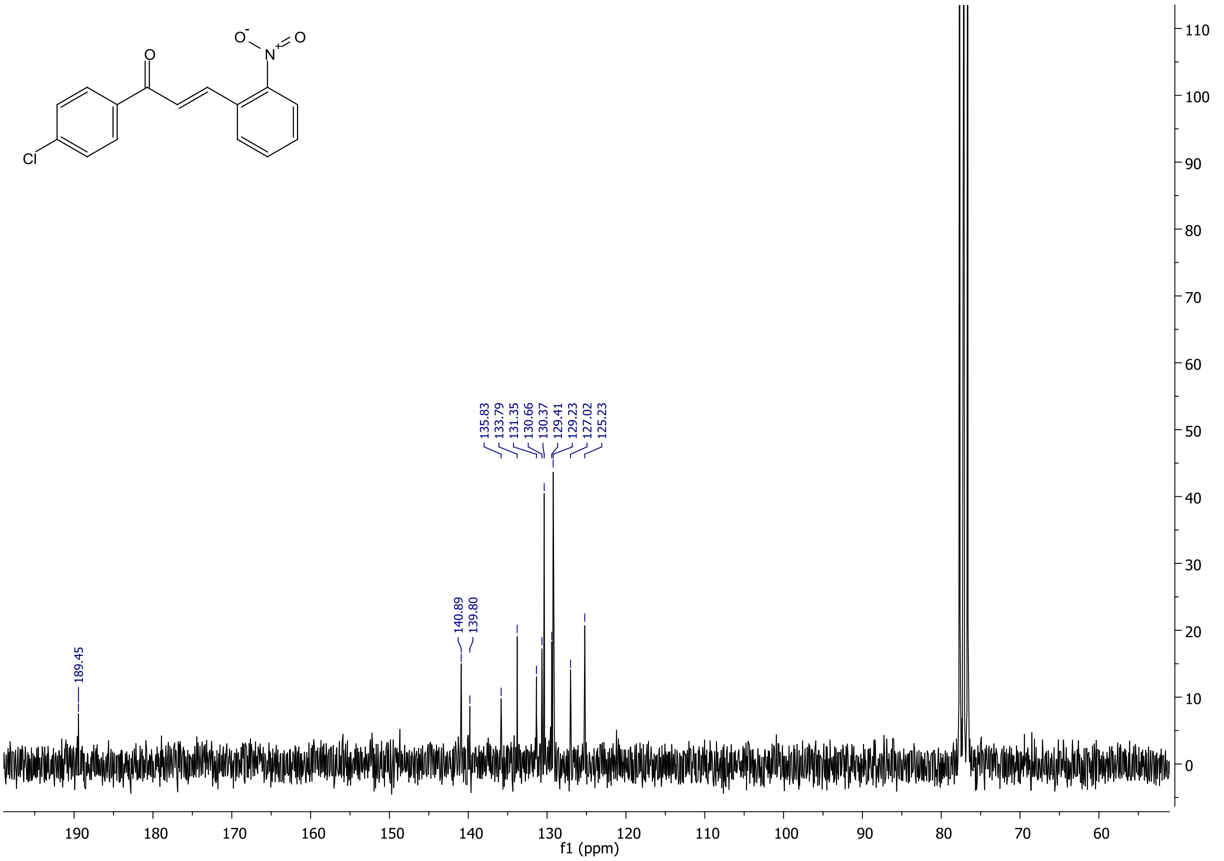

Figure S22. (*E*)-1-(4-bromophenyl)-3-phenyl-2-propen-1-one **3w**.

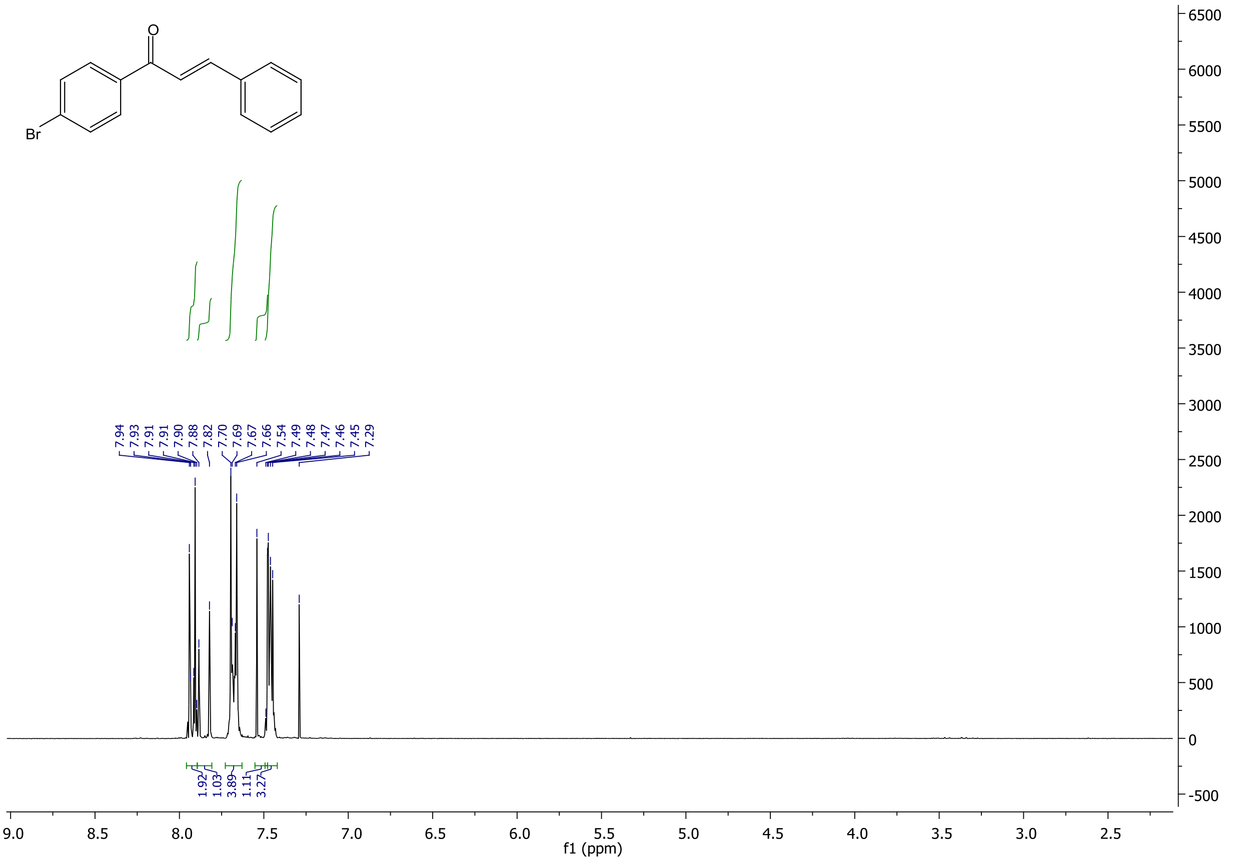

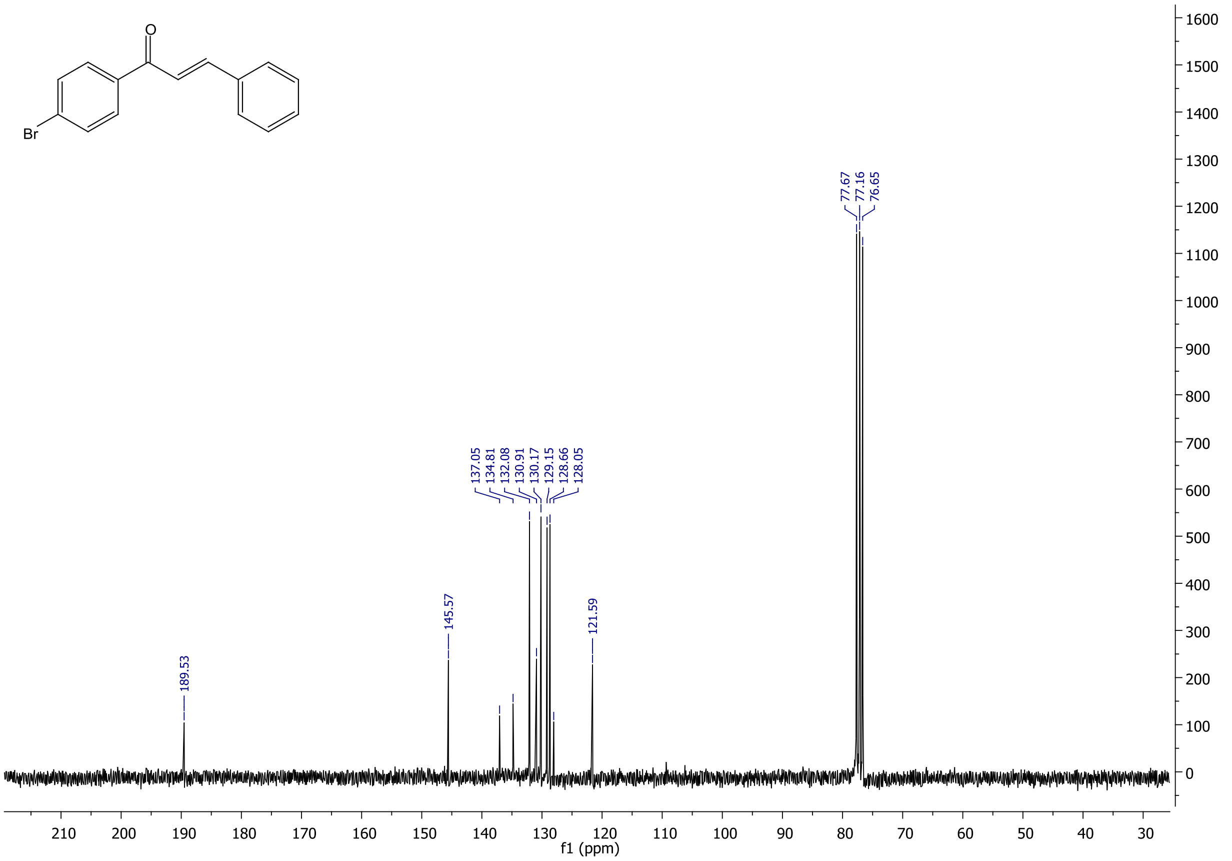

Figure S23. (*E*)-1-(4-bromophenyl)-3-(4-bromophenyl)-2-propen-1-one **3x**.

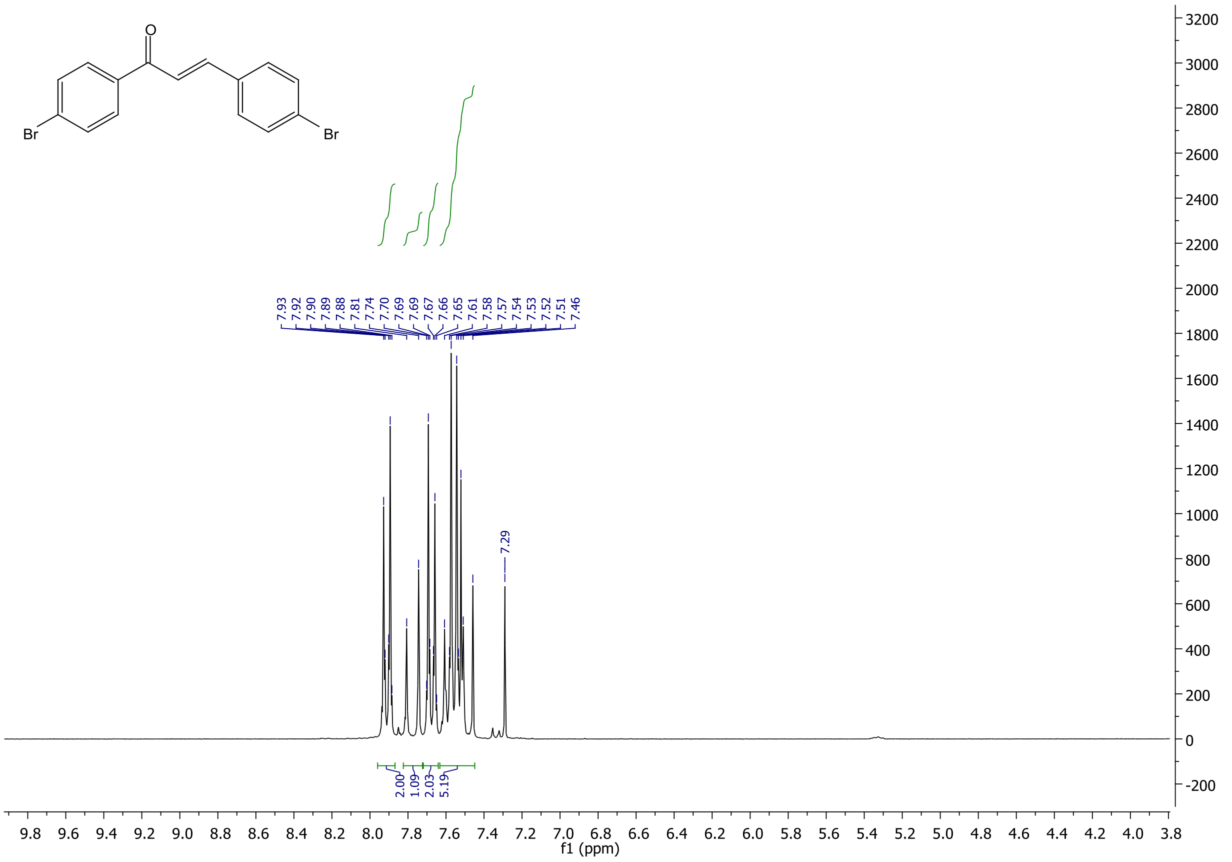

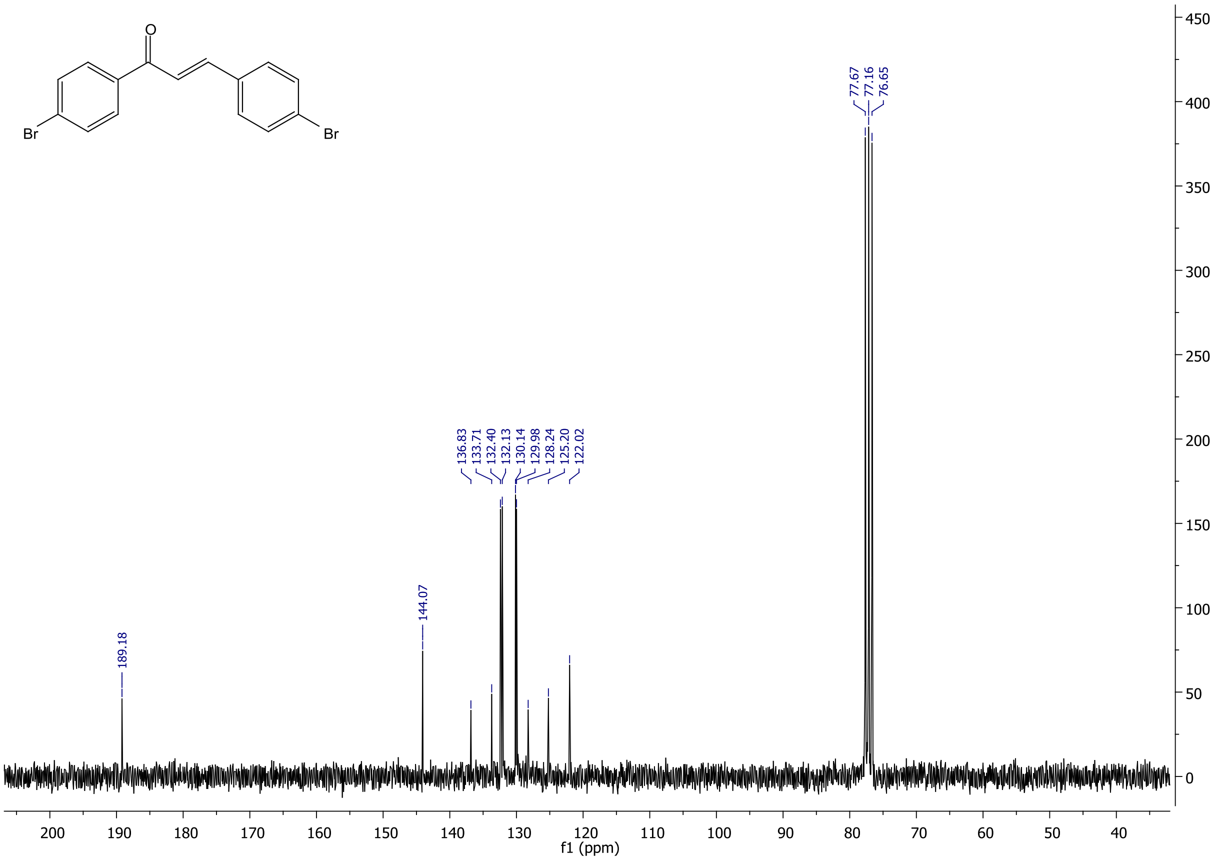

Figure S24. (*E*)-1-(4-bromophenyl)-3-(4-nitrophenyl)-2-propen-1-one **3y**.

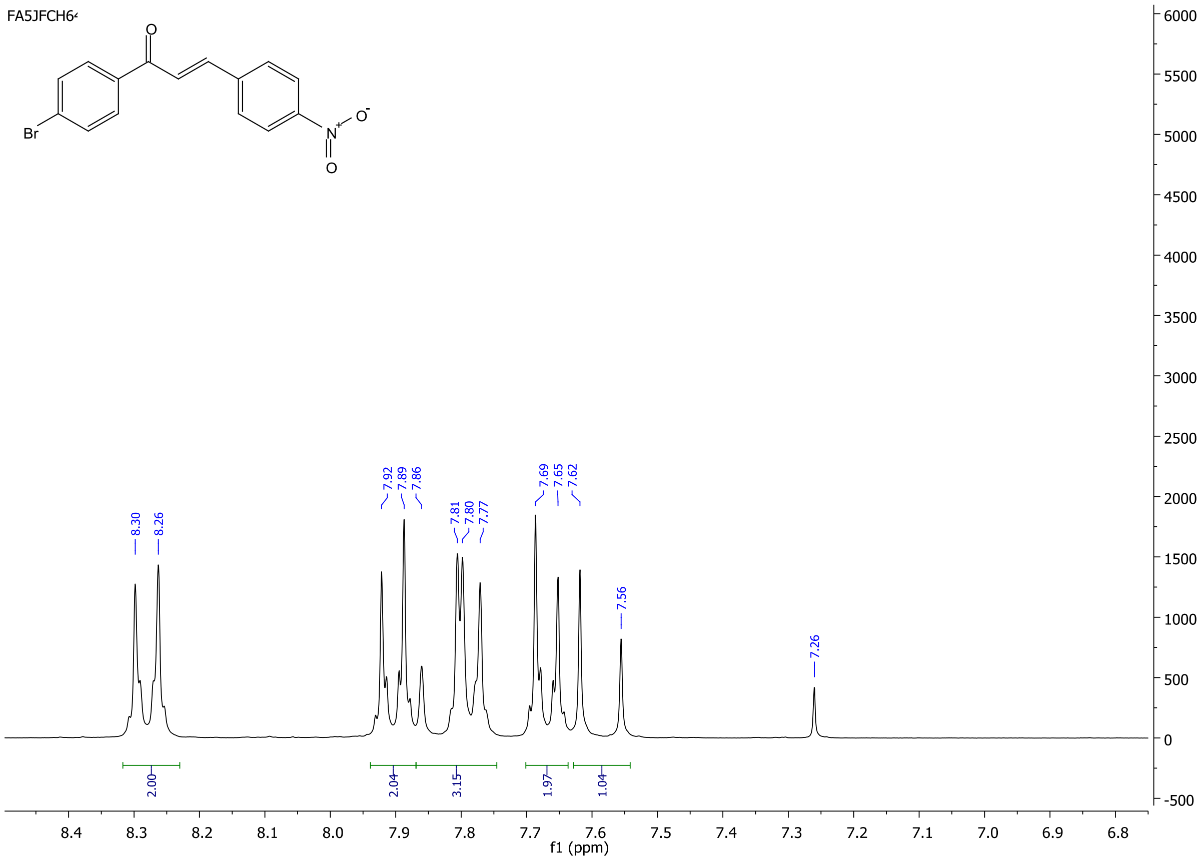

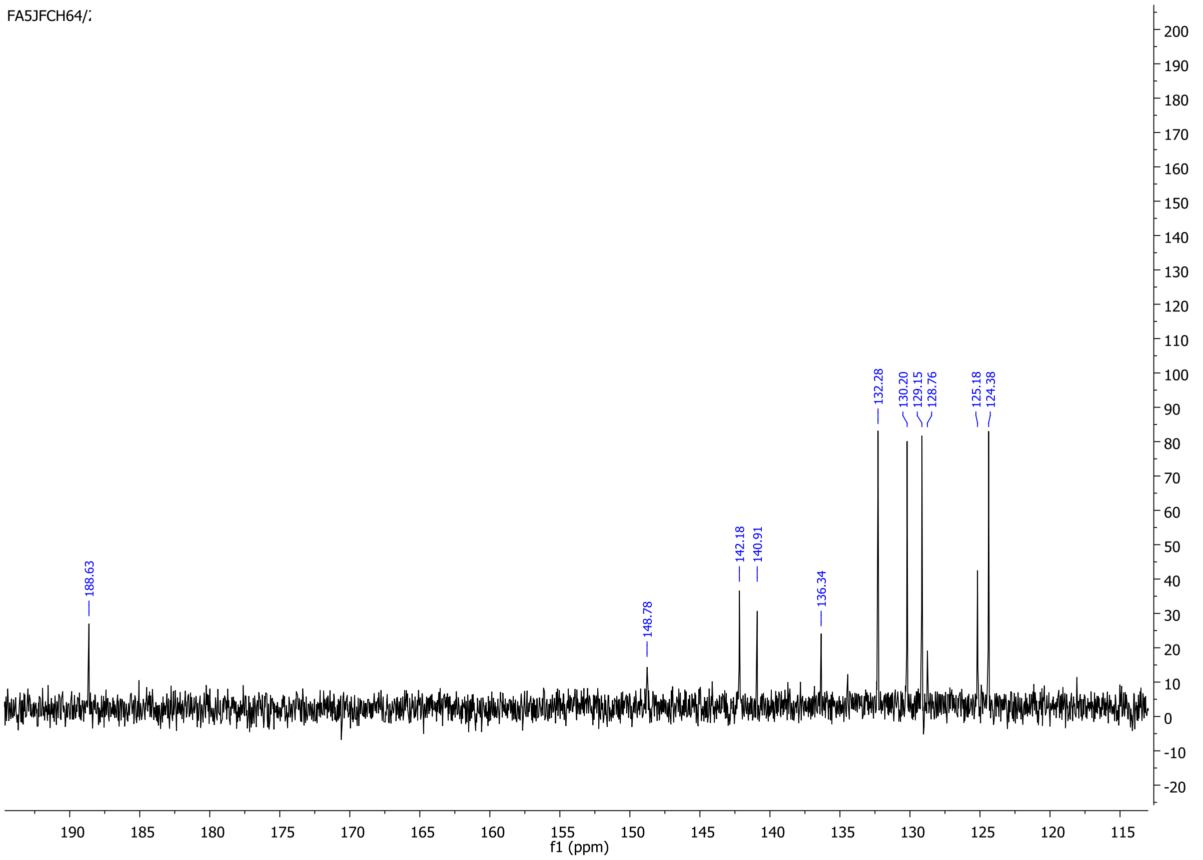

Figure S25. (*E*)-1-(4-nitrophenyl)-3-phenyl-2-propen-1-one **3z.**

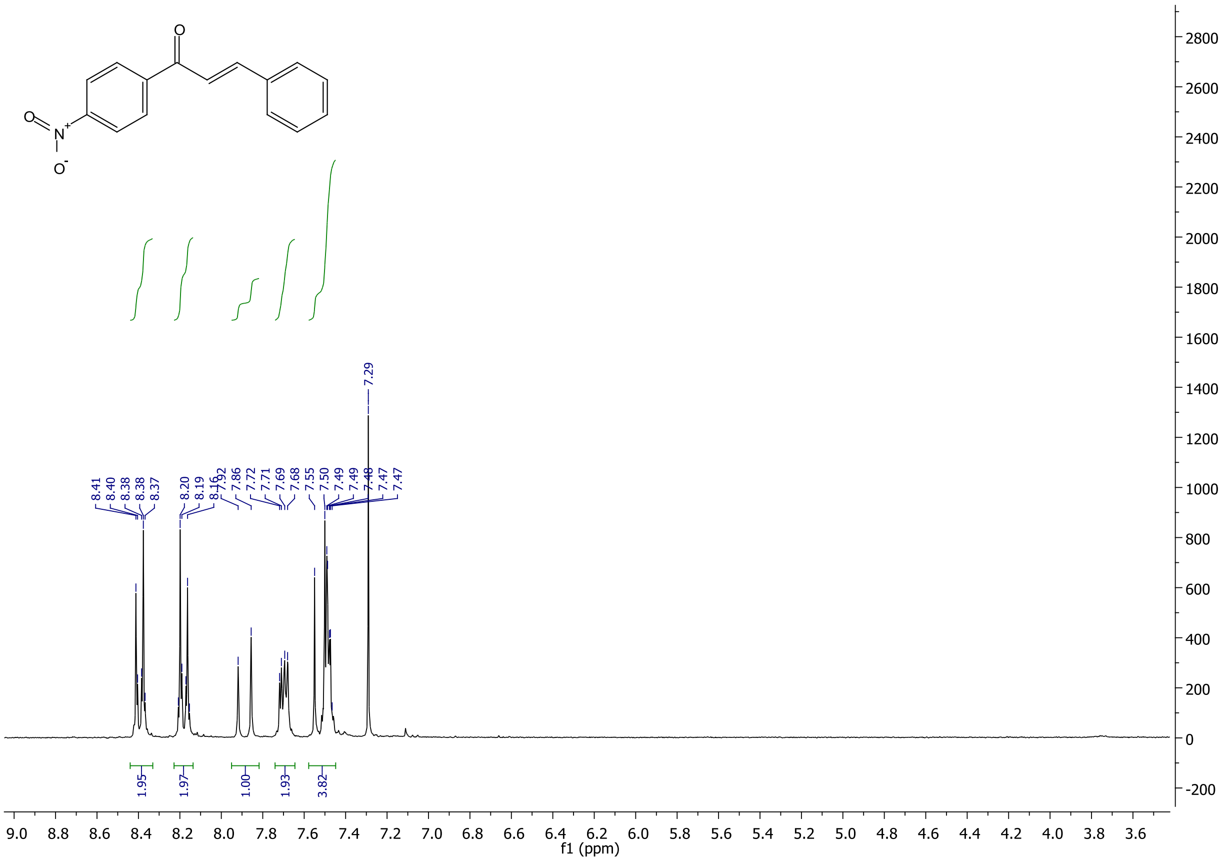

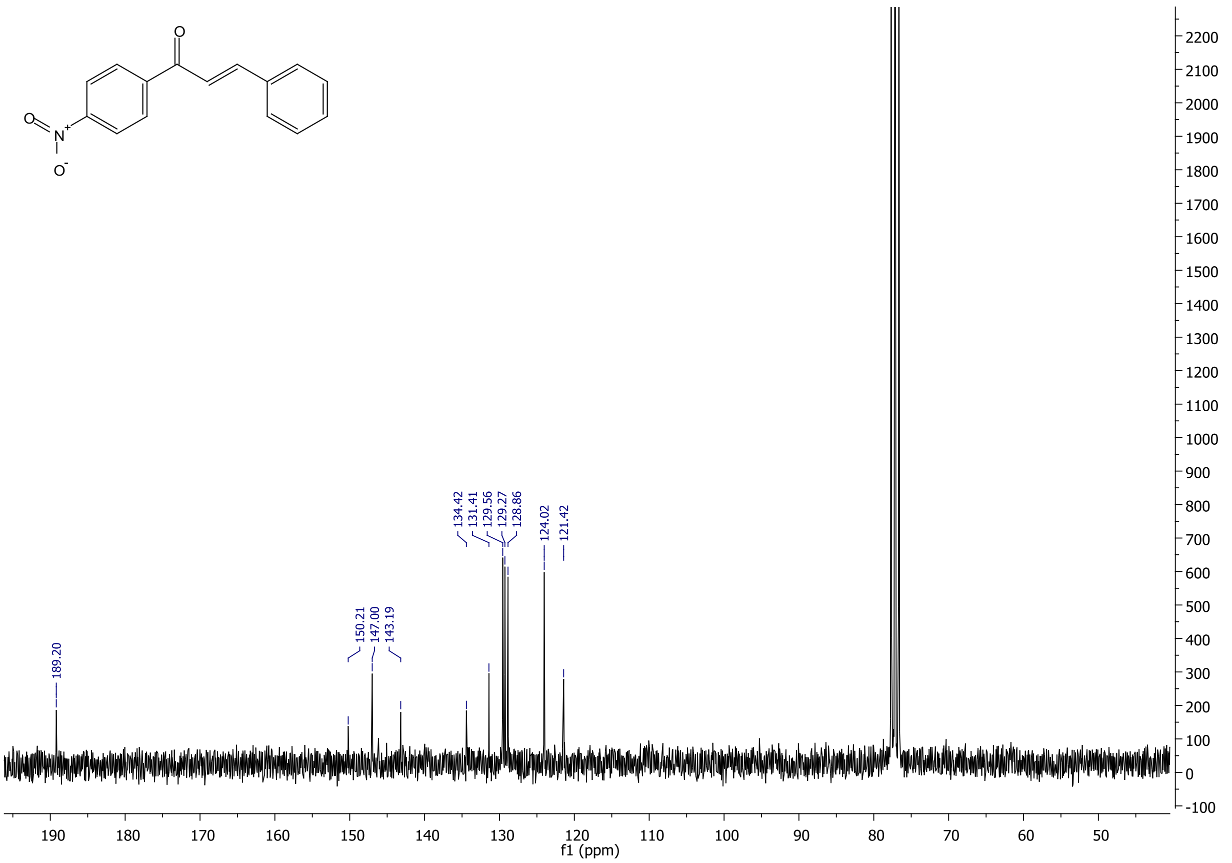

Figure S26. (*E*)-3-(4-methoxyphenyl)-1-(4-nitrophenyl)-2-propen-1-one **3aa**.

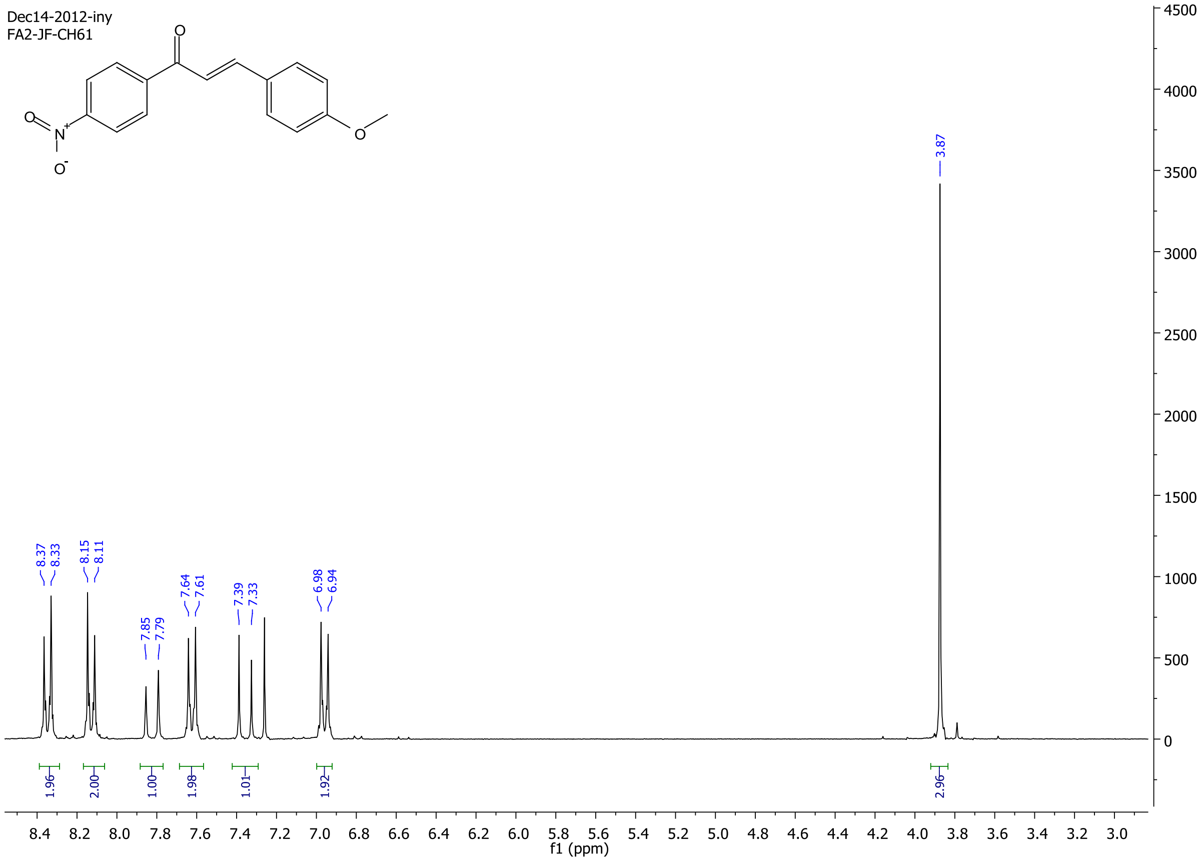

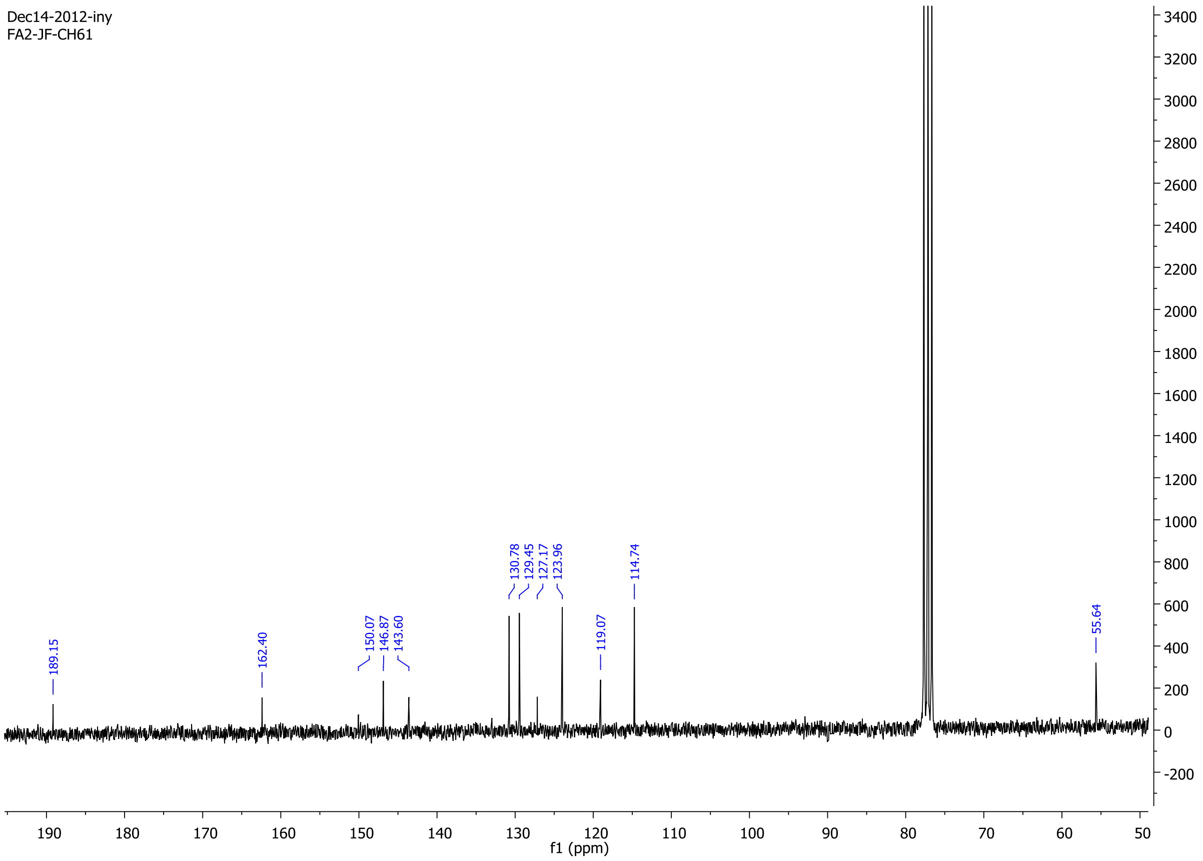

Figure S27. (*E*)-3-(4-methilphenyl)-1-(4-nitrophenyl)-2-propen-1-one **3ab**.

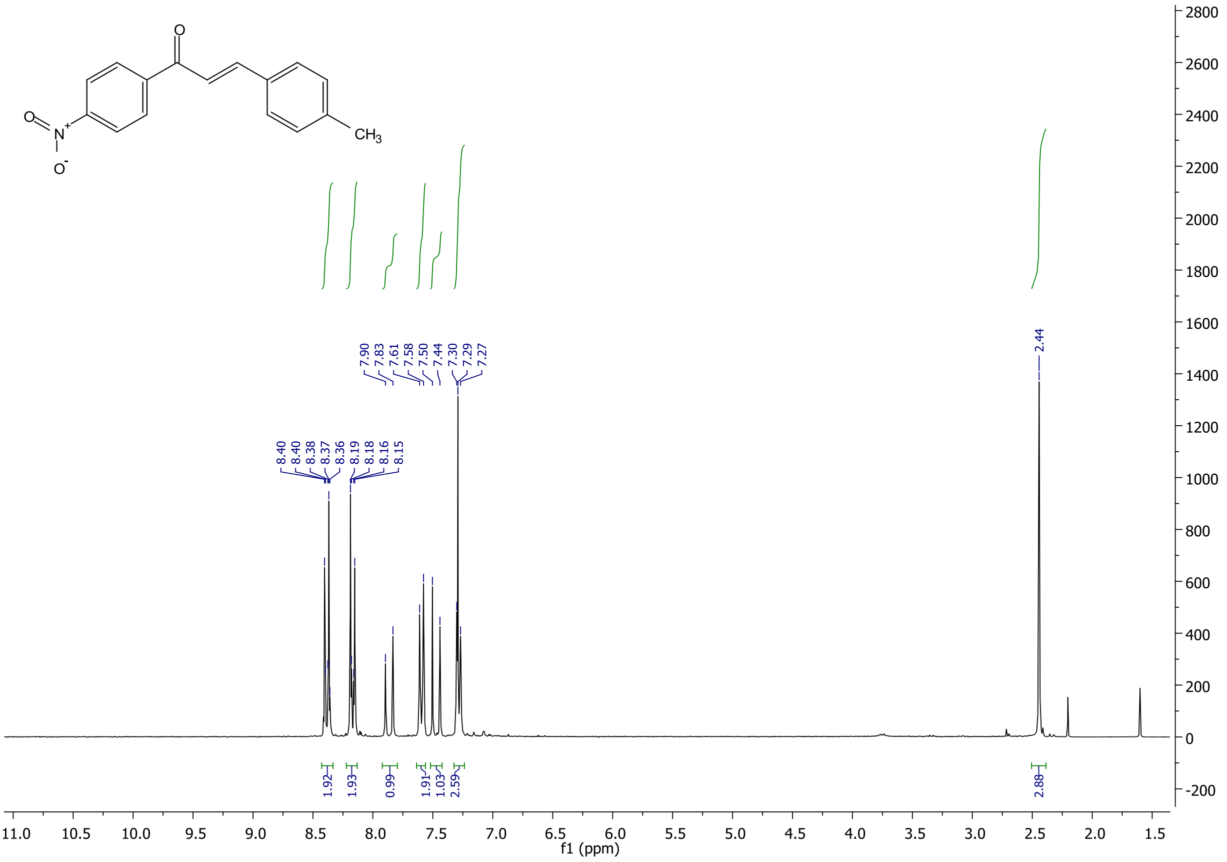

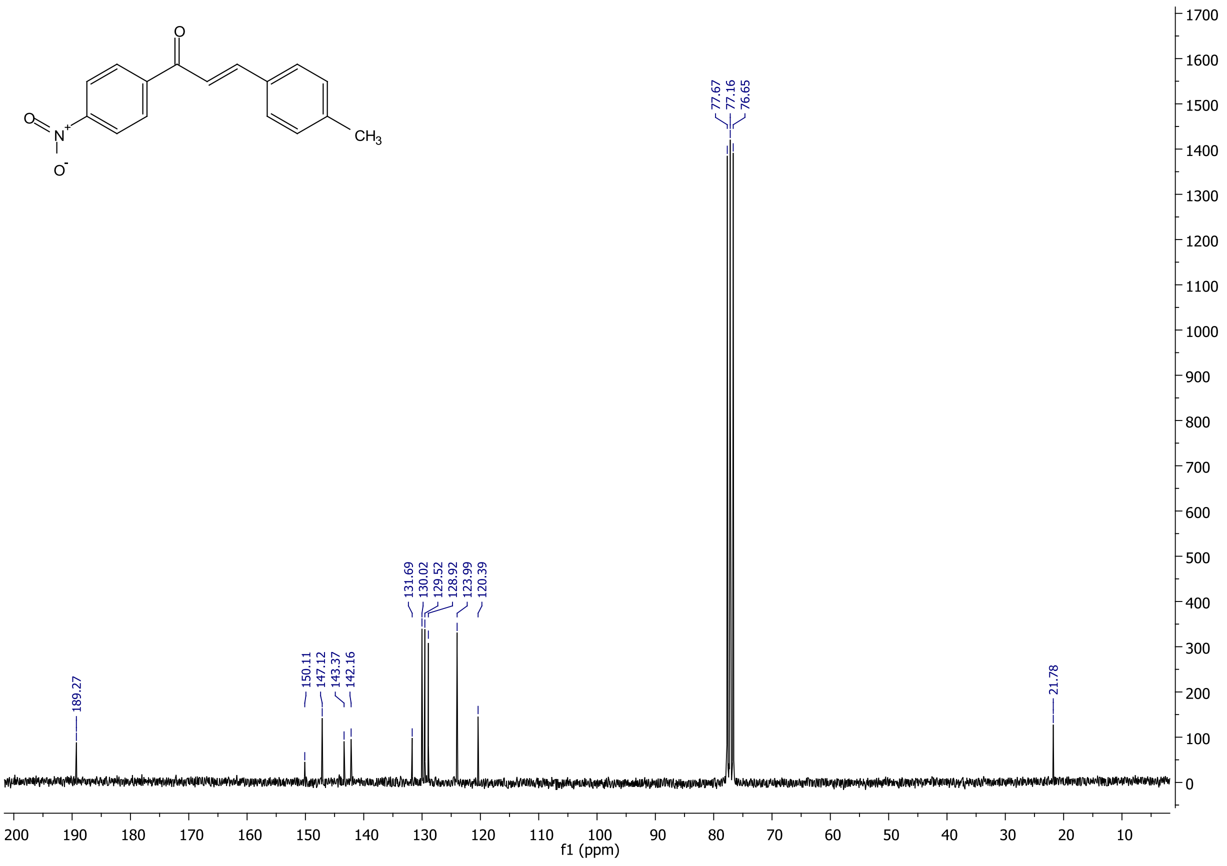

Figure S28. (*E*)-3-(4-bromophenyl)-1-(4-nitrophenyl)-2-propen-1-one **3ac**.

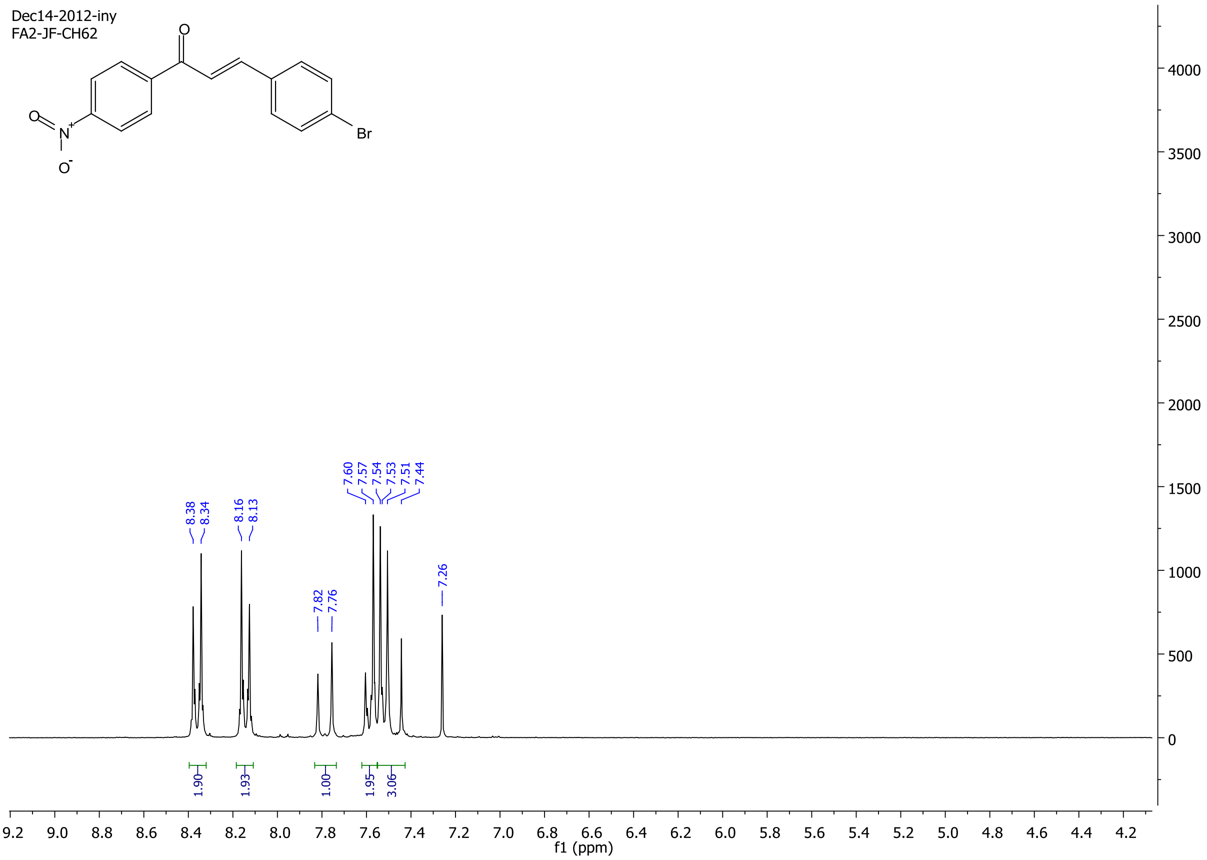

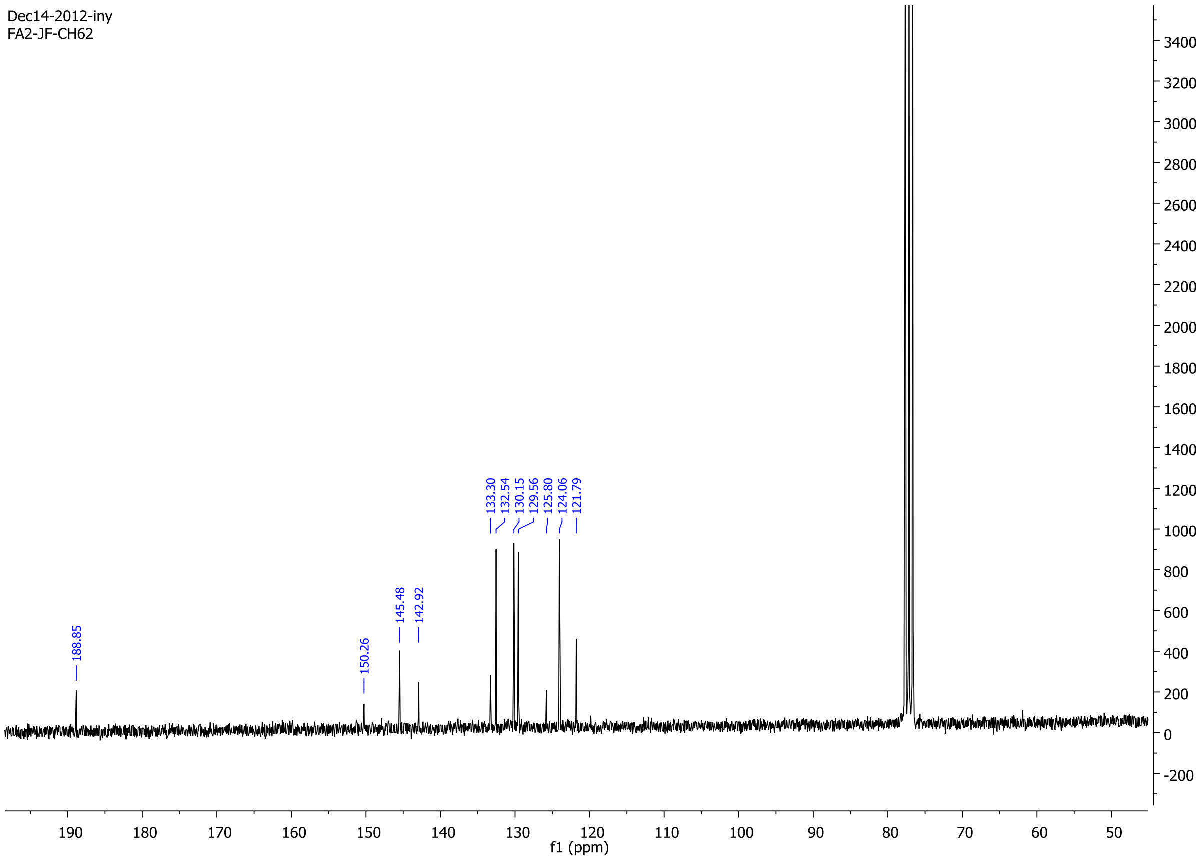

Figure S29. (*E*)-3-(4-nitrophenyl)-1-(4-nitrophenyl)-2-propen-1-one **3ad**.

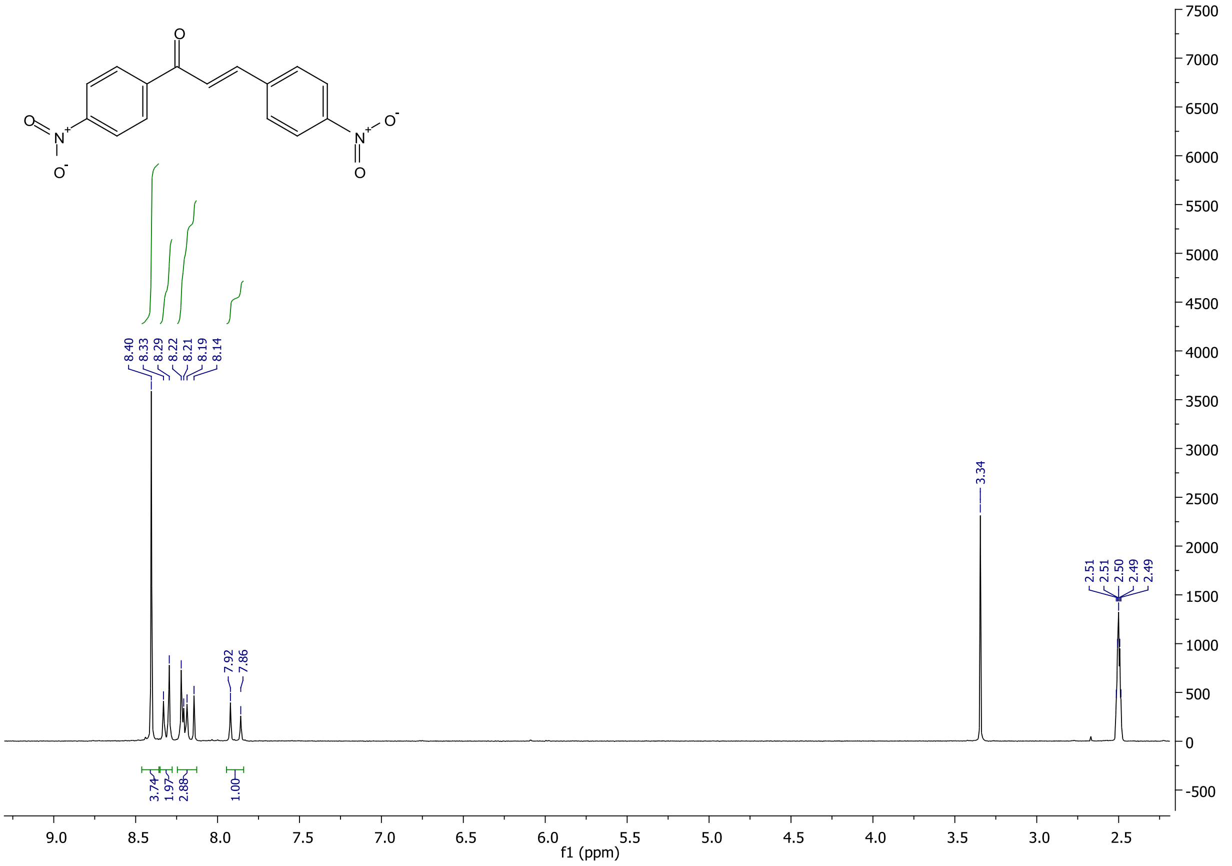

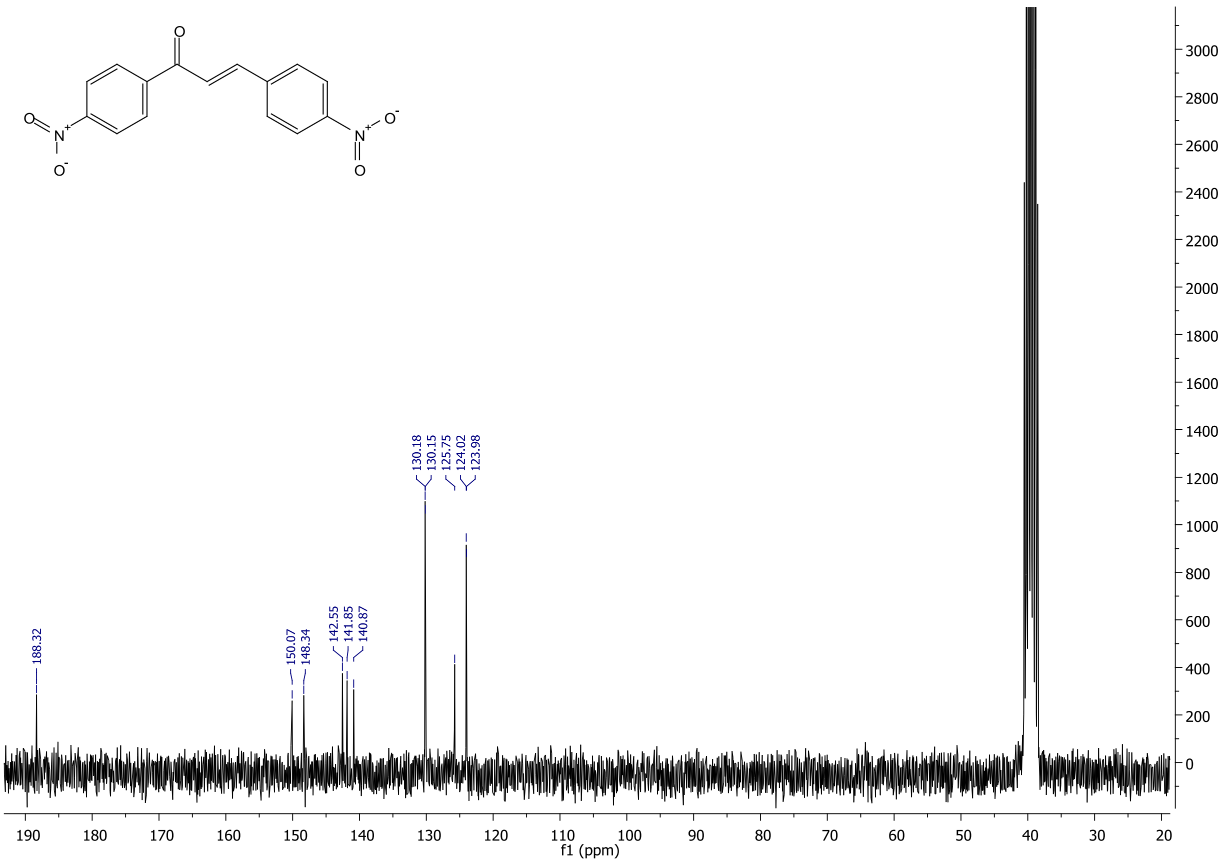

Figure S30. (*E*)-3-(5-chloro-2-nitrophenyl)-1-phenyl-2-propen-1-one **3ae**.

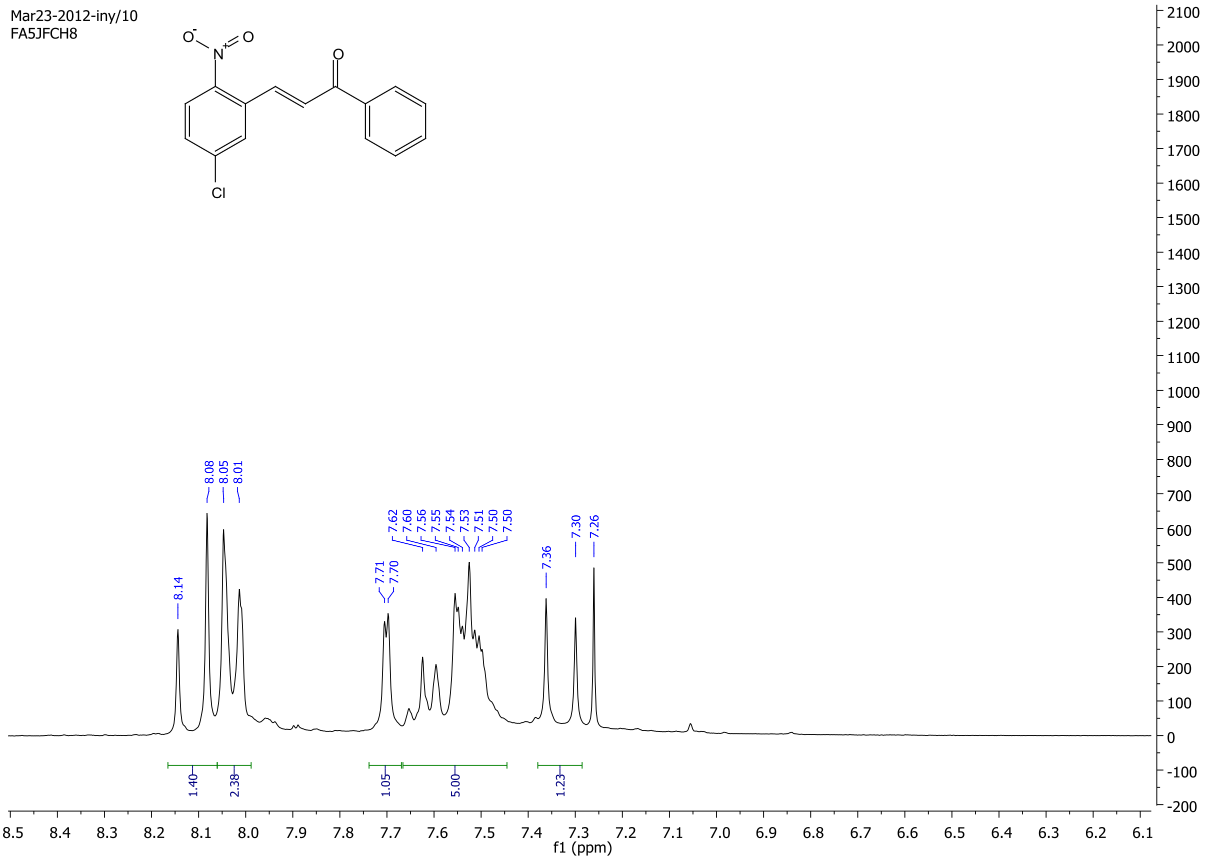

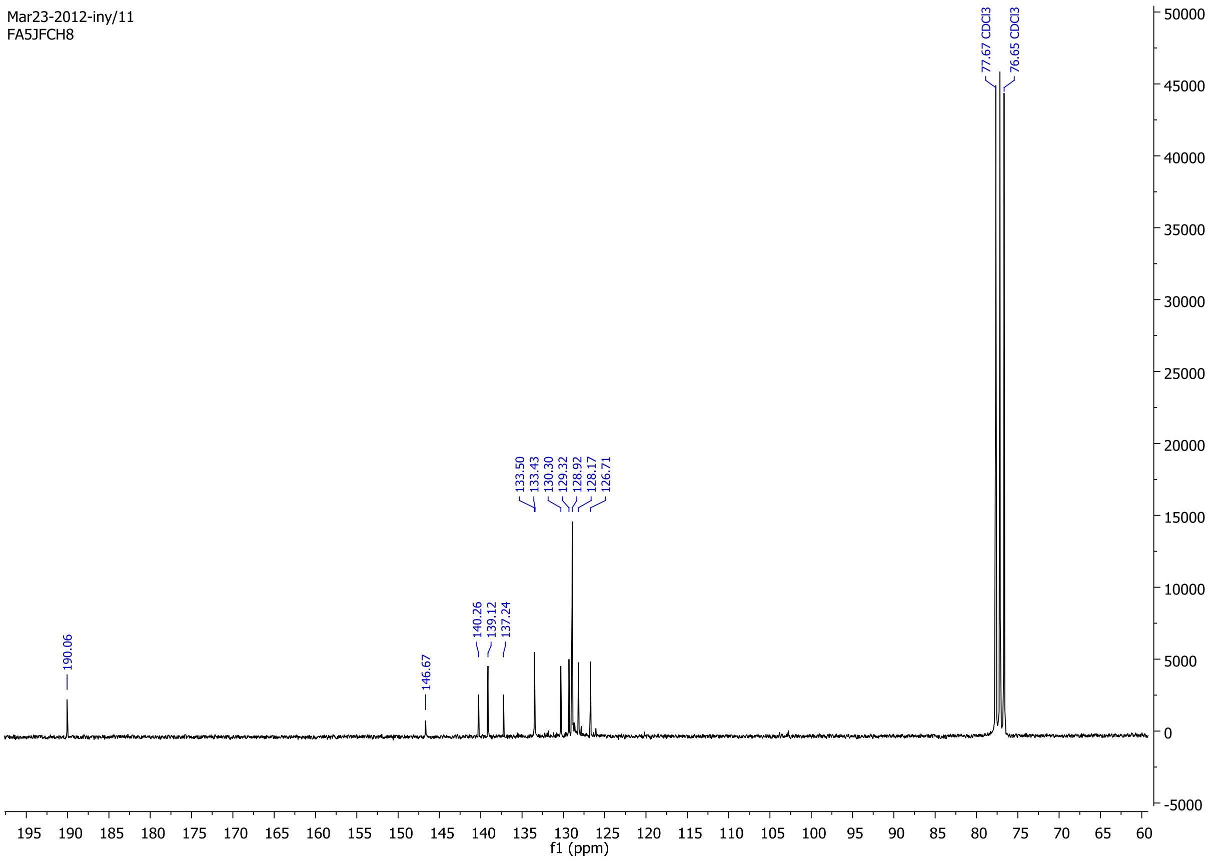

Figure S31. (*E*)-3-(4,5-Dimethoxy-2-nitrophenyl)-1-phenyl-2-propen-1-one **3af**.

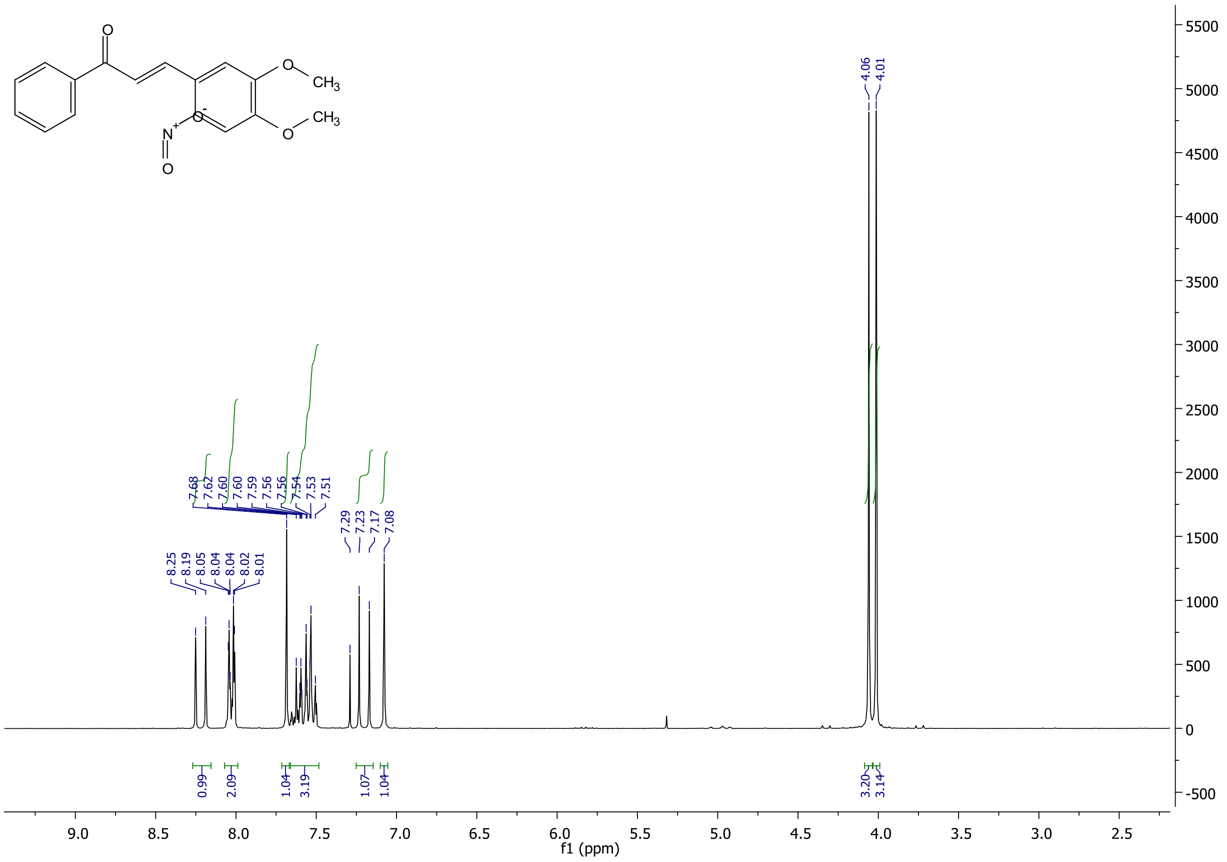

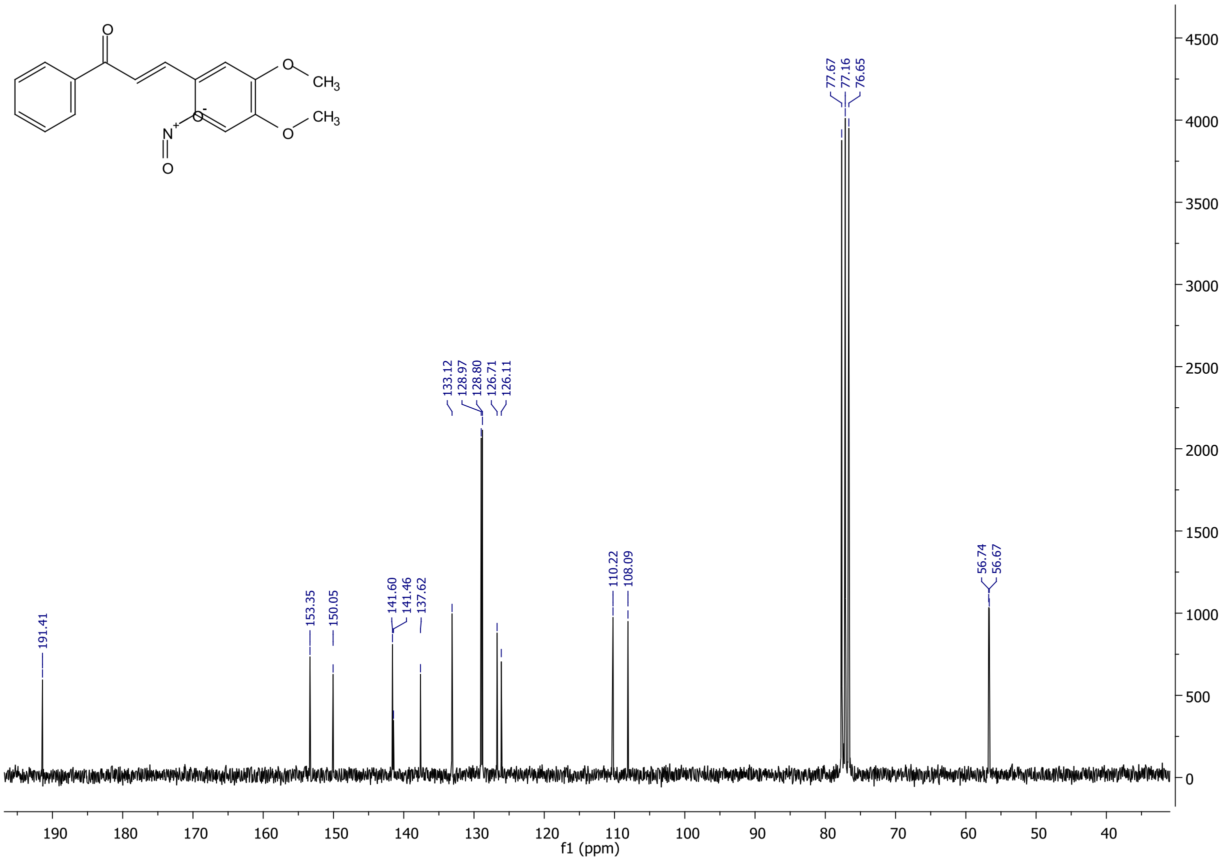

Figure S32. (*E*)-3-(5-Bromo-2-nitrophenyl)-1-(*p*-tolyl)-2-propen-1-one **3ag**.

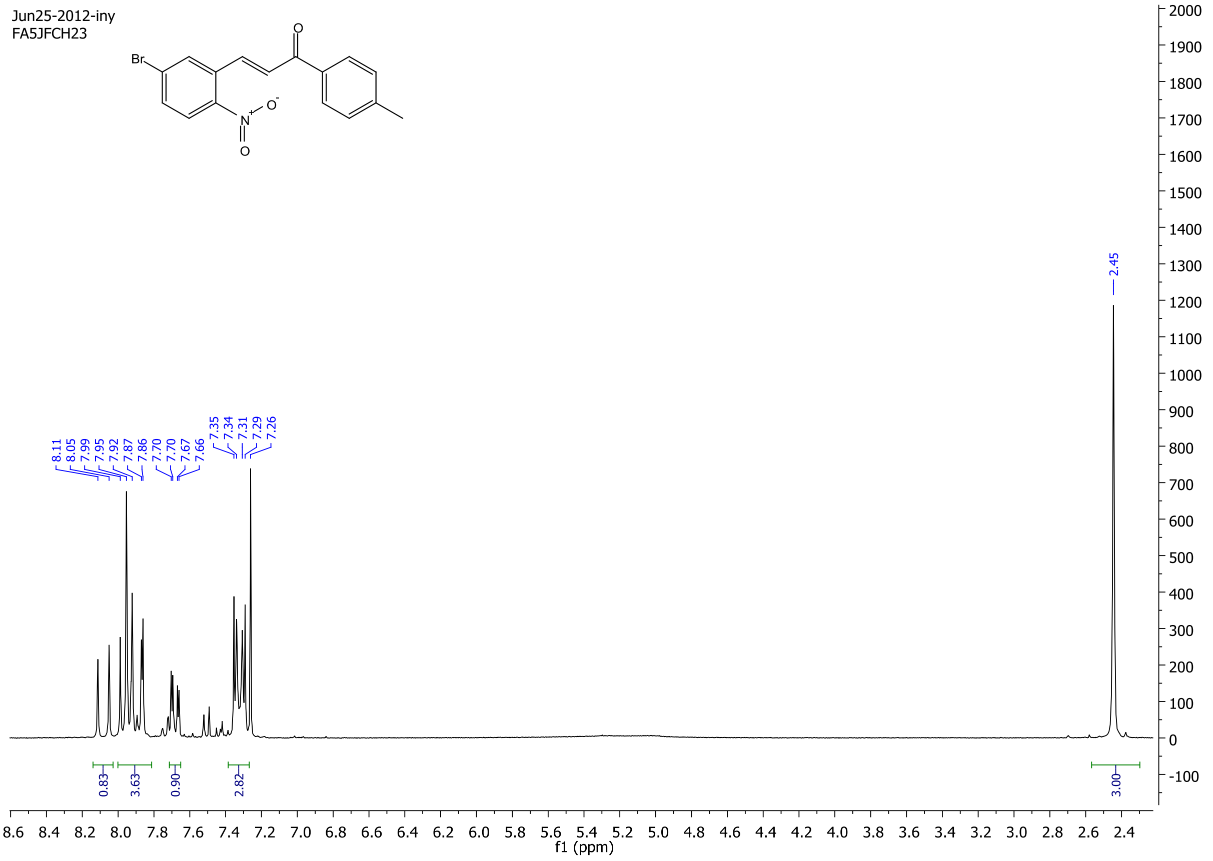

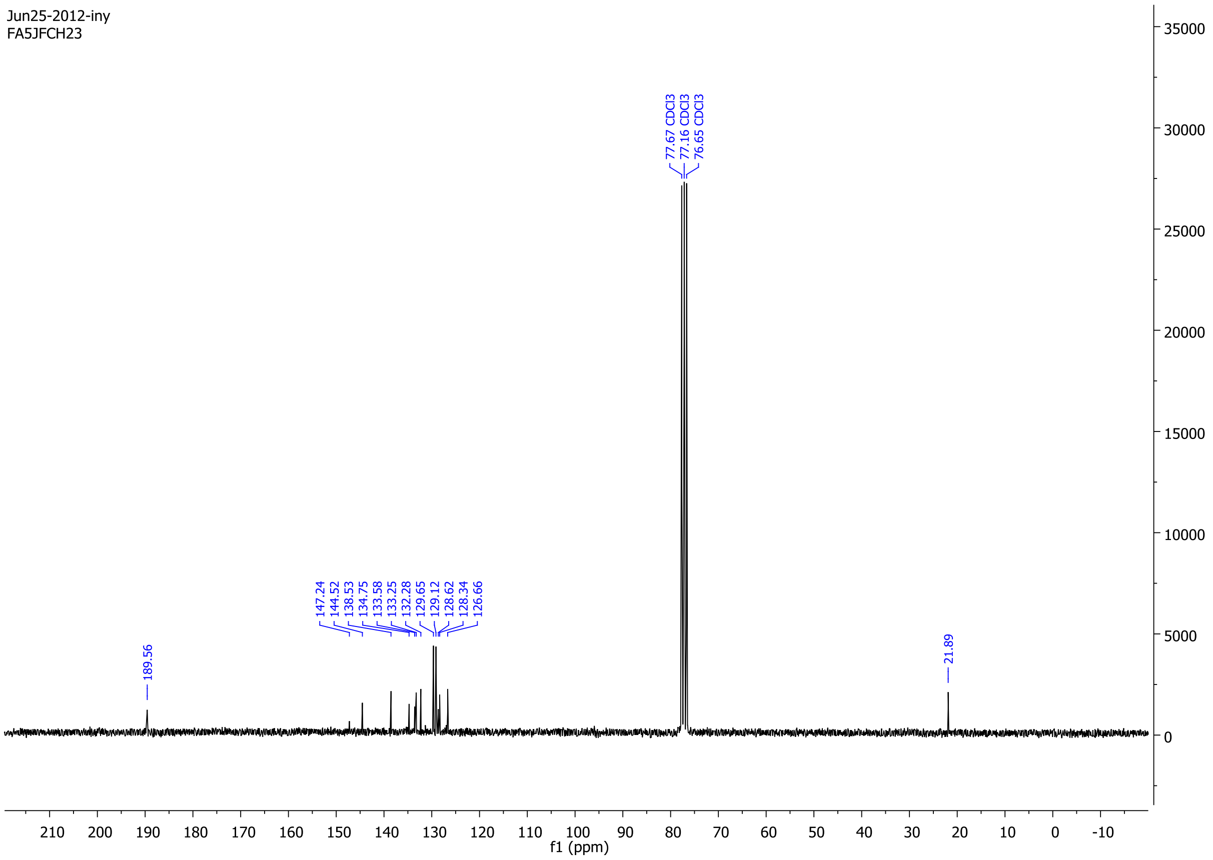

Figure S33. (*E*)-3-(4,5-Dimethoxy-2-nitrophenyl)-1-(*p*-tolyl)-2-propen-1-one **3ah**.

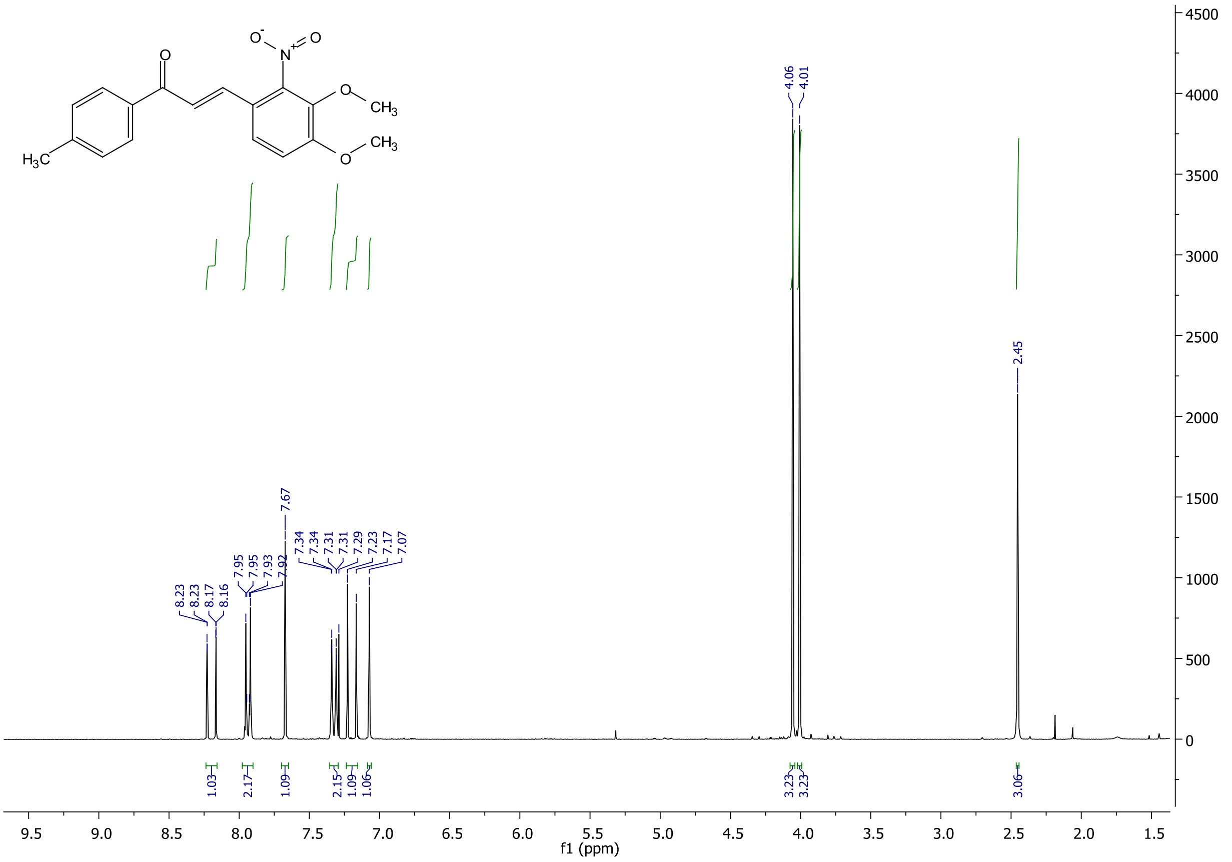

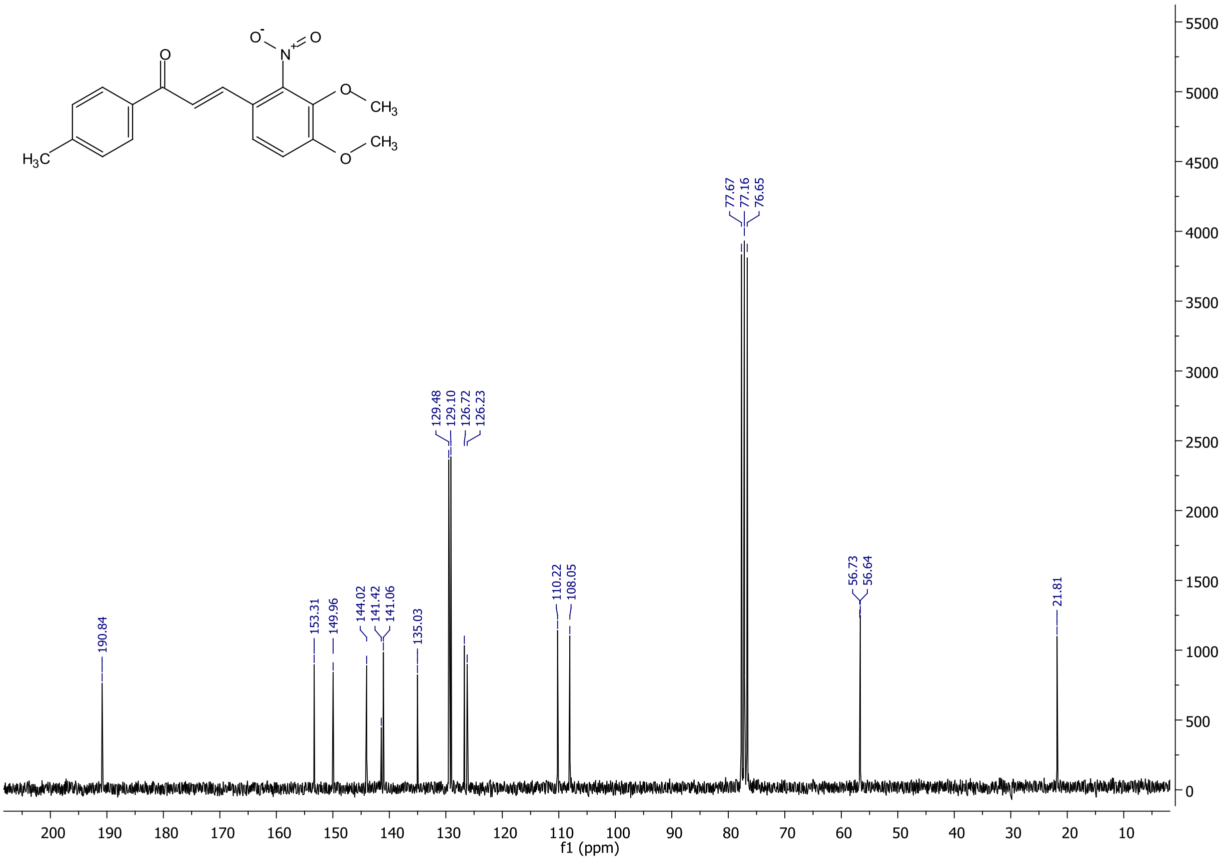

Figure S34. (*E*)-3-(5-Bromo-2-nitrophenyl)-1-(4-bromophenyl)-2-propen-1-one **3ai**.

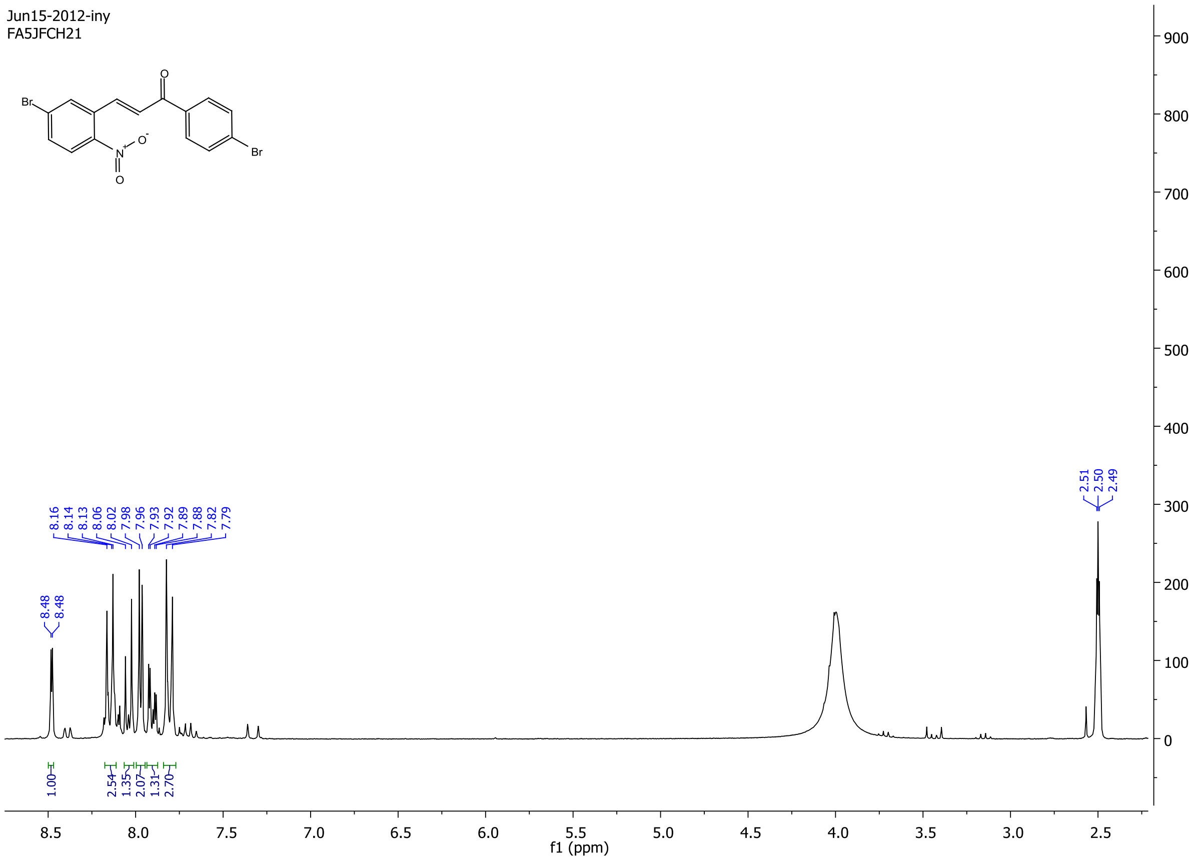

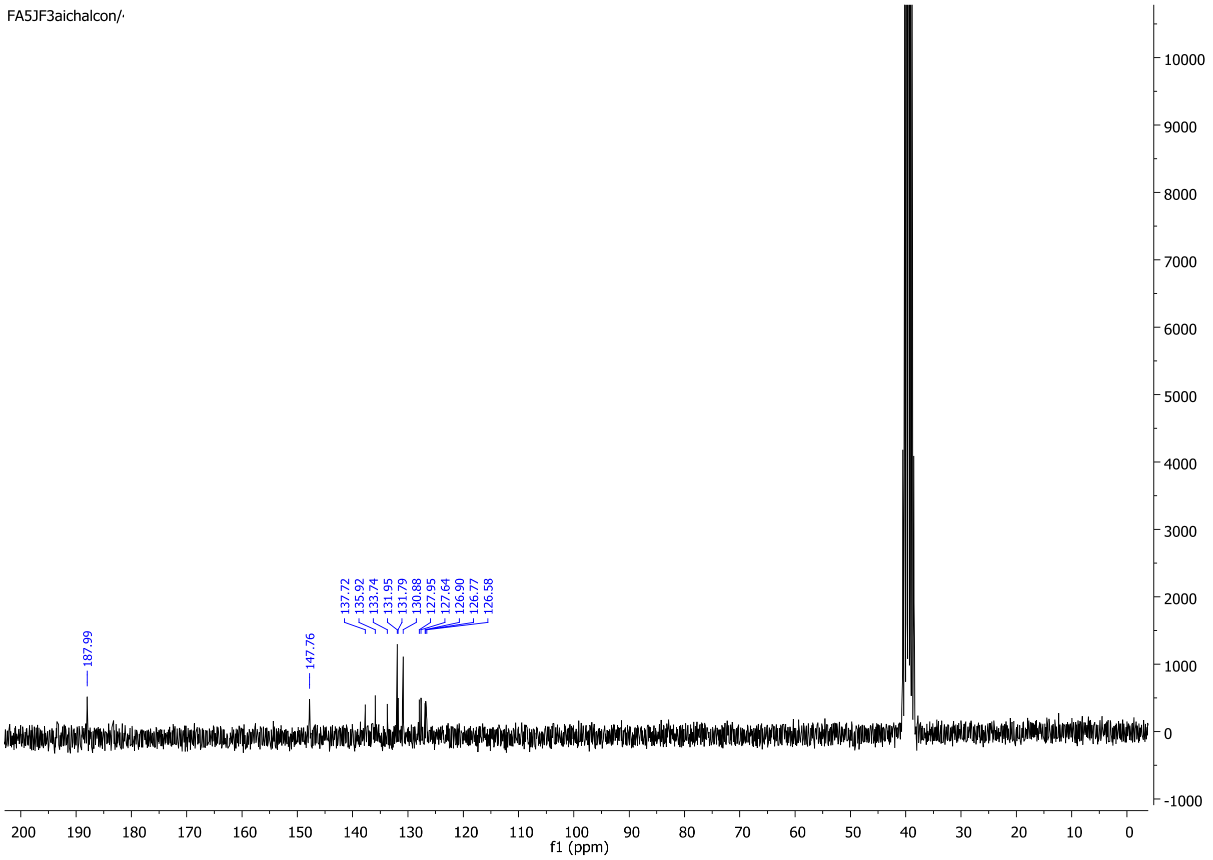

Figure S35. (*E*)-1-Phenyl-3-(furan-2-yl)-2-propen-1-one **3aj**.

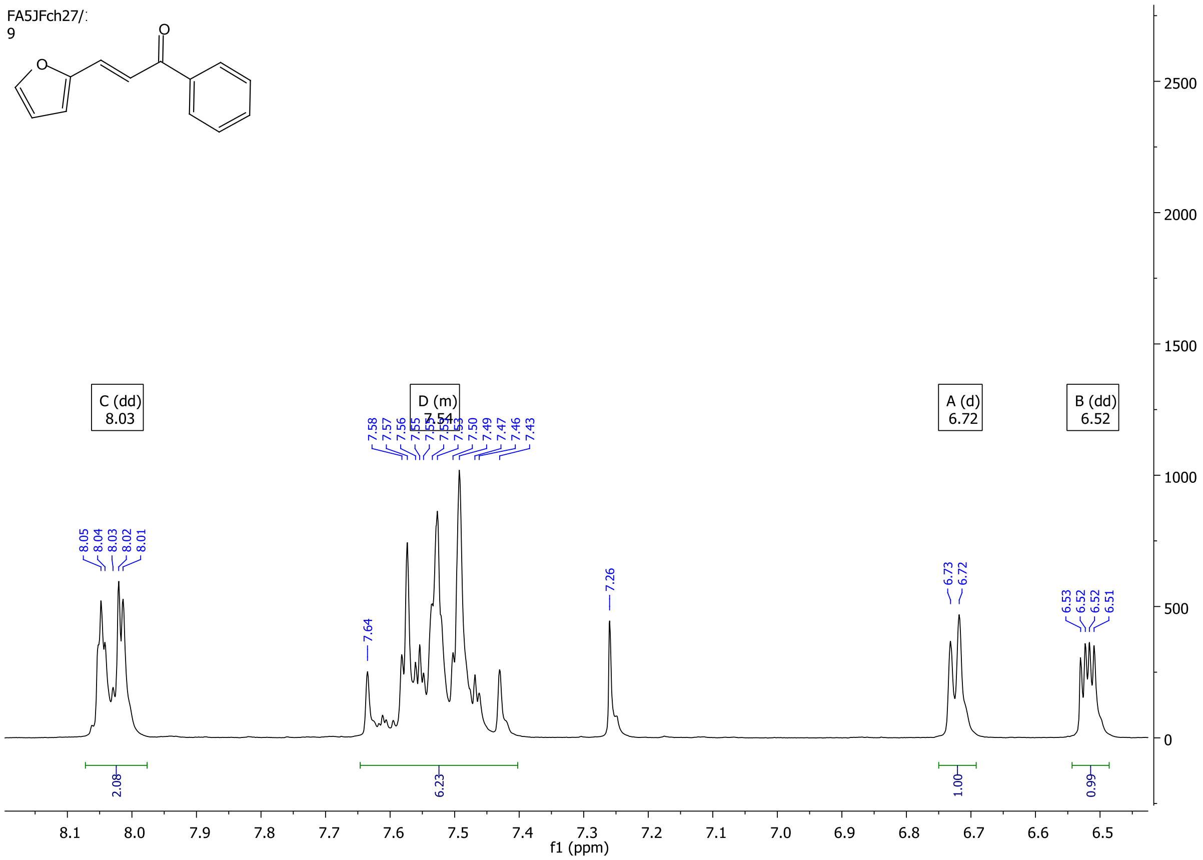

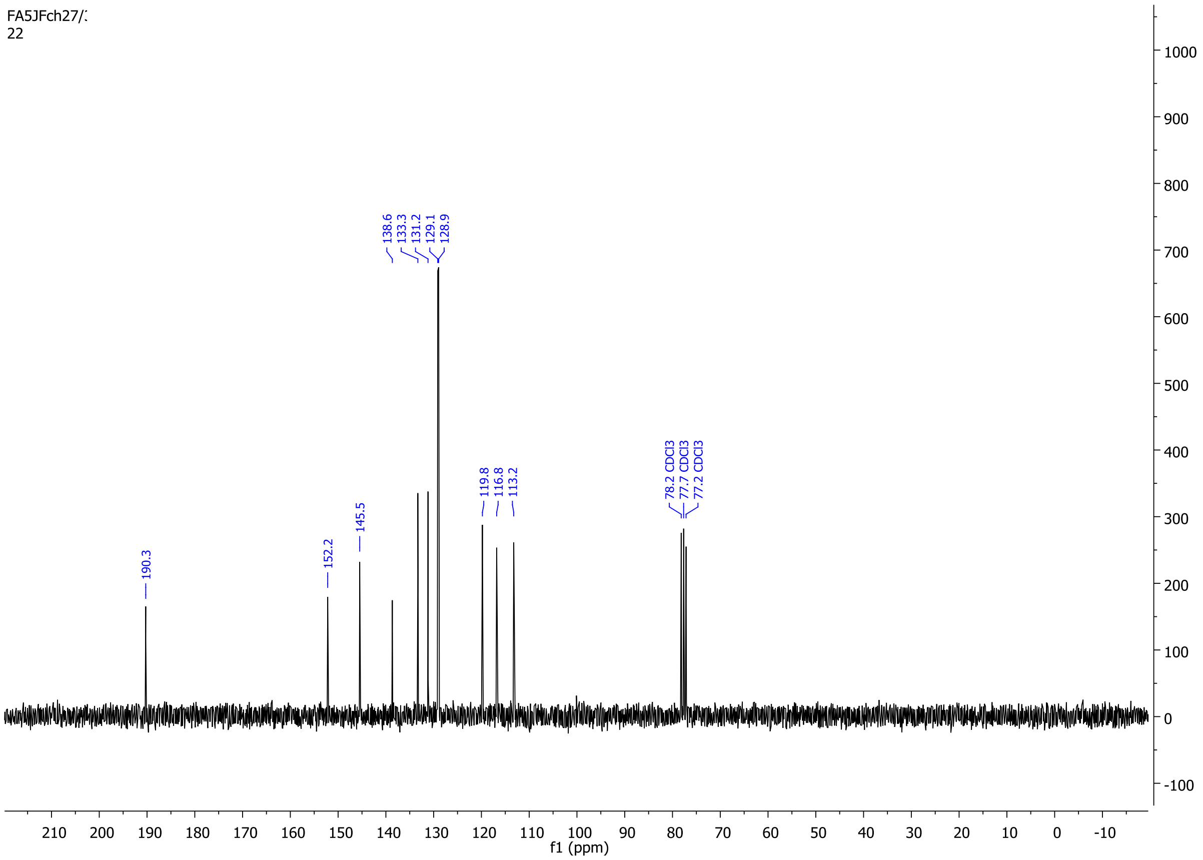

Figure S36. (*E*)-3-Phenyl-1-(furan-2-yl)-2-propen-1-one **3ak**.

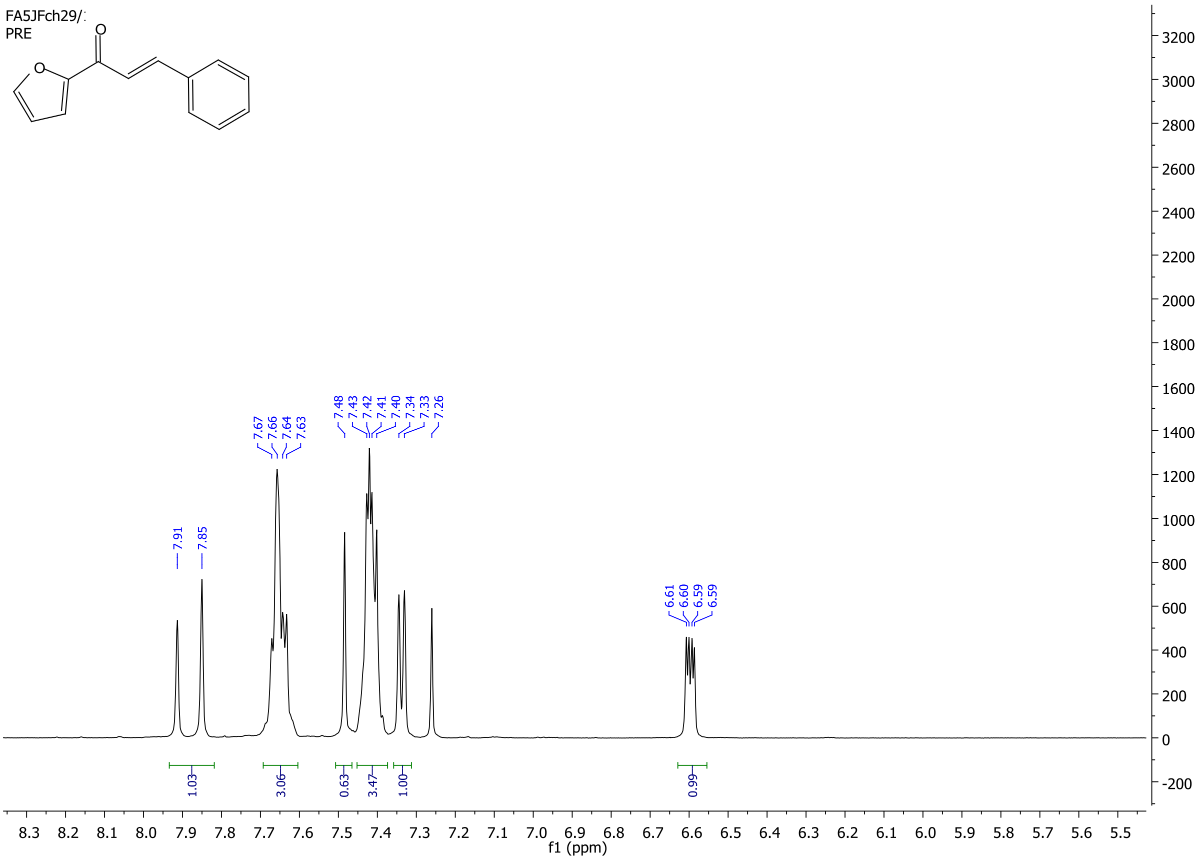

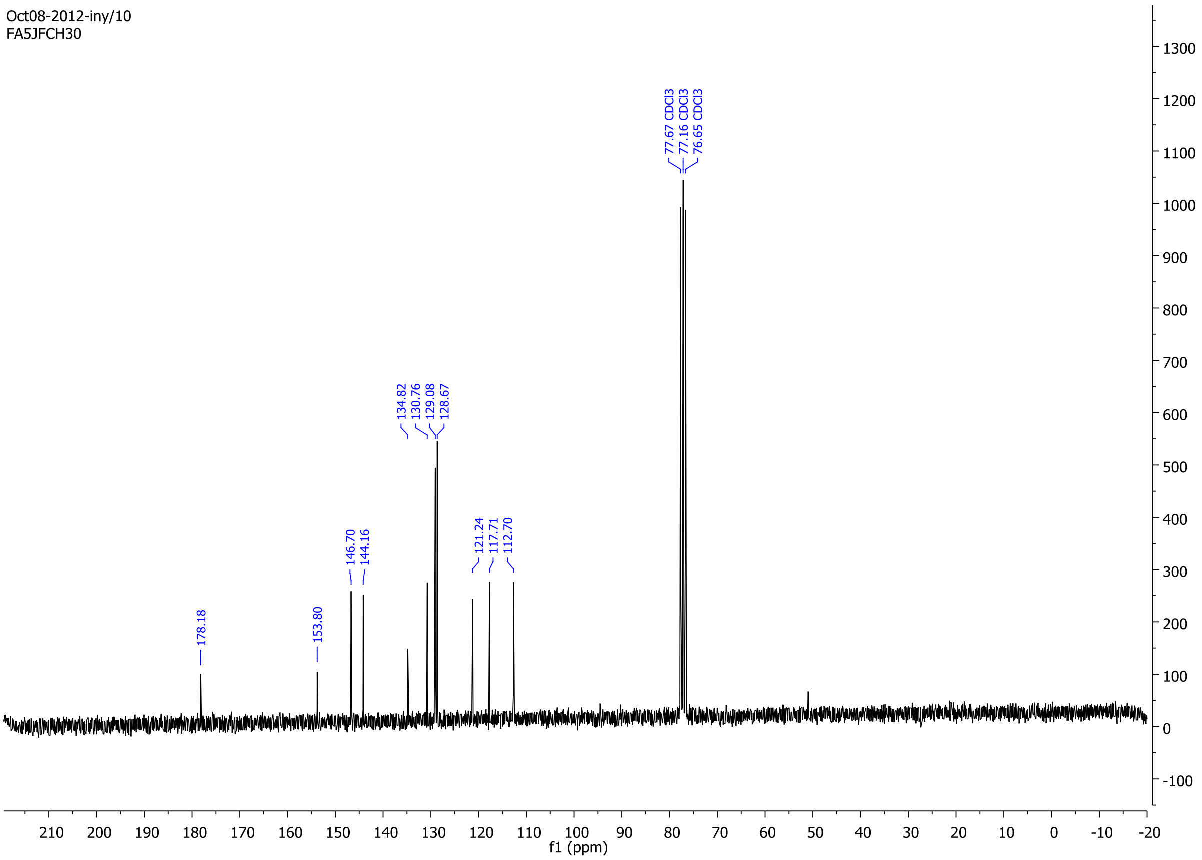

Figure S37. (*E*)-1,3-Di-(furan-2-yl)-2-propen-1-one **3al**.

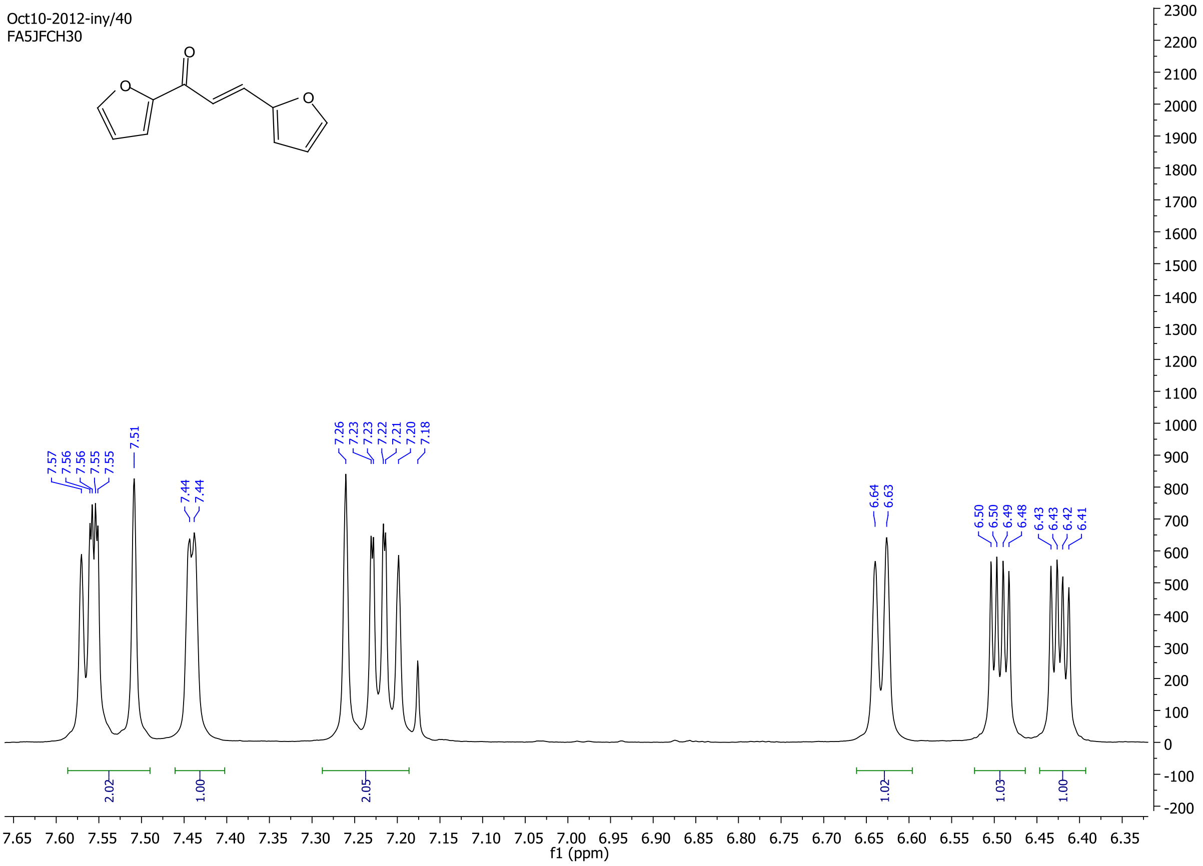

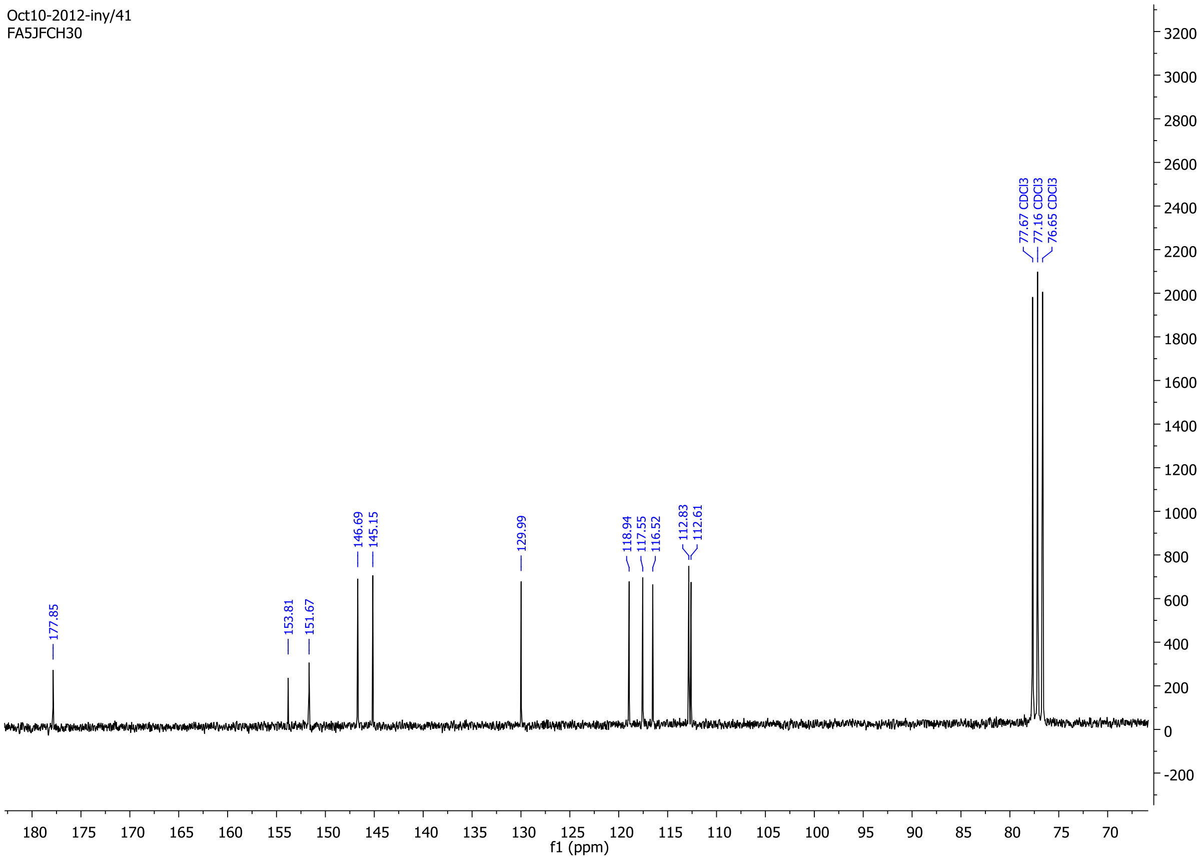

Figure S38. (*E*)-1-(Furan-2-yl)-3-(2-nitrophenyl)-2-propen-1-one **3am**.

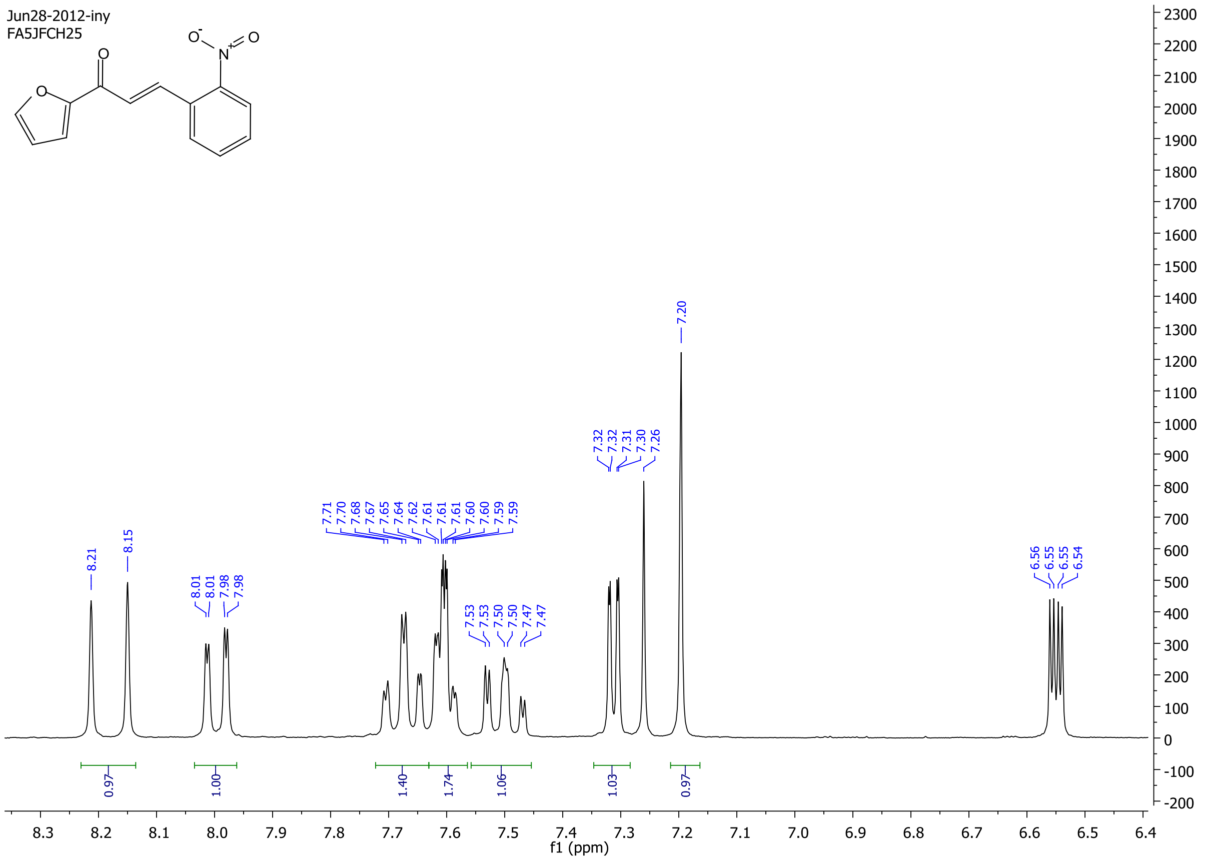

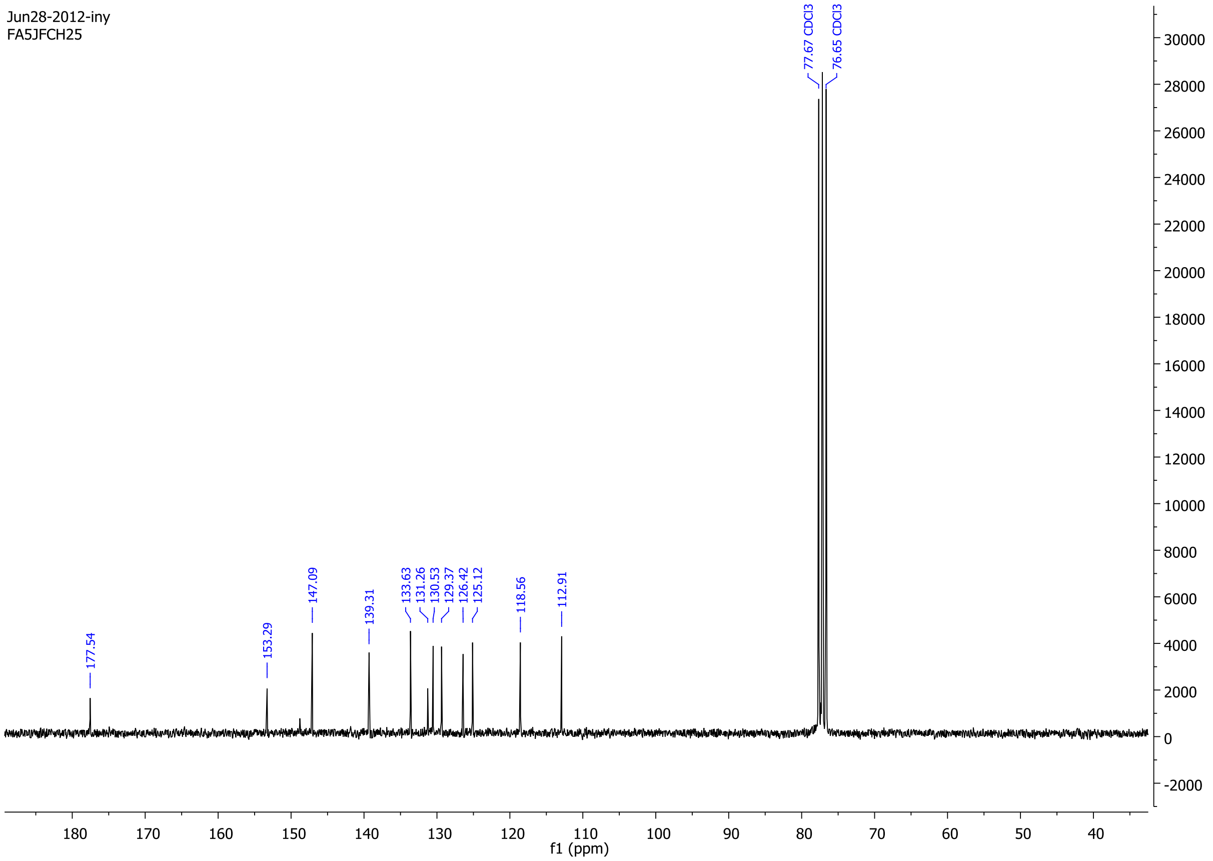

Figure S39. (*E*)-3-Phenyl-1-(thiophen-2-yl)-2-propen-1-one **3an**.

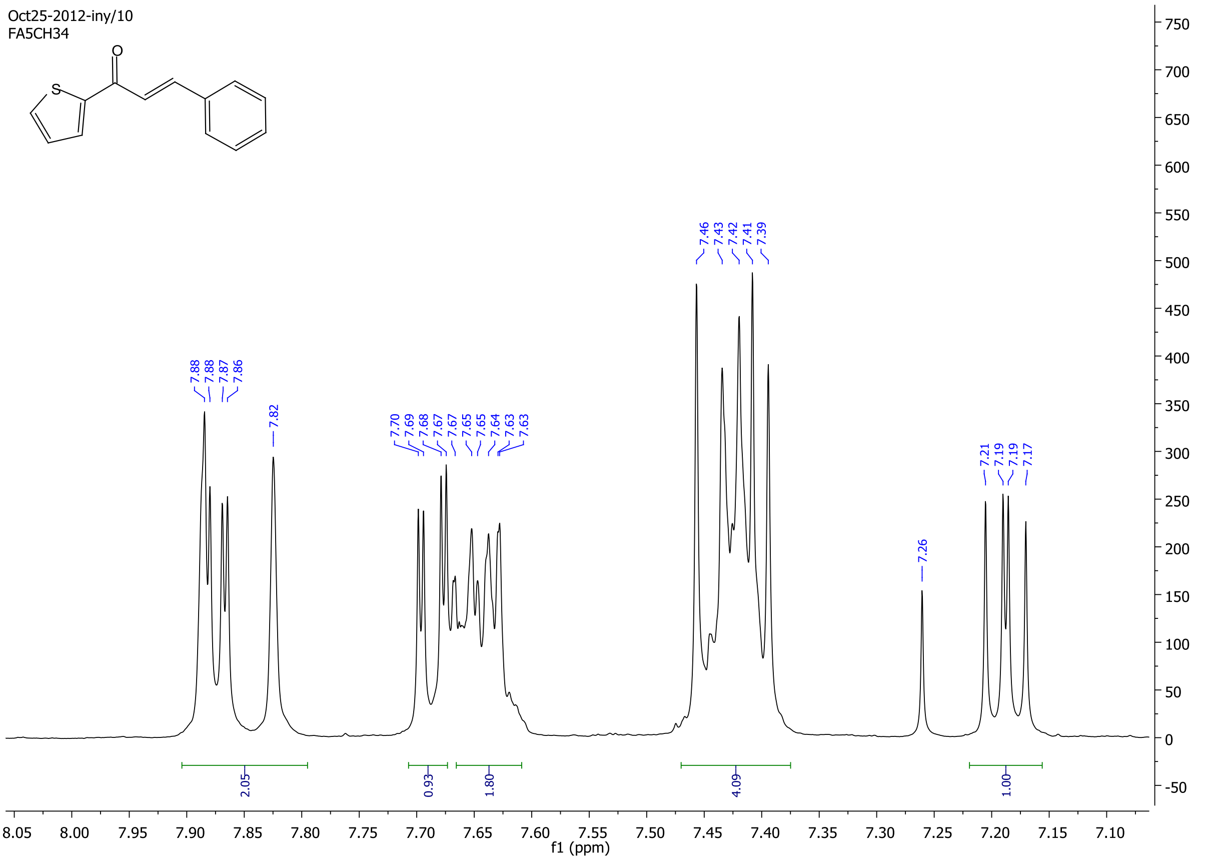

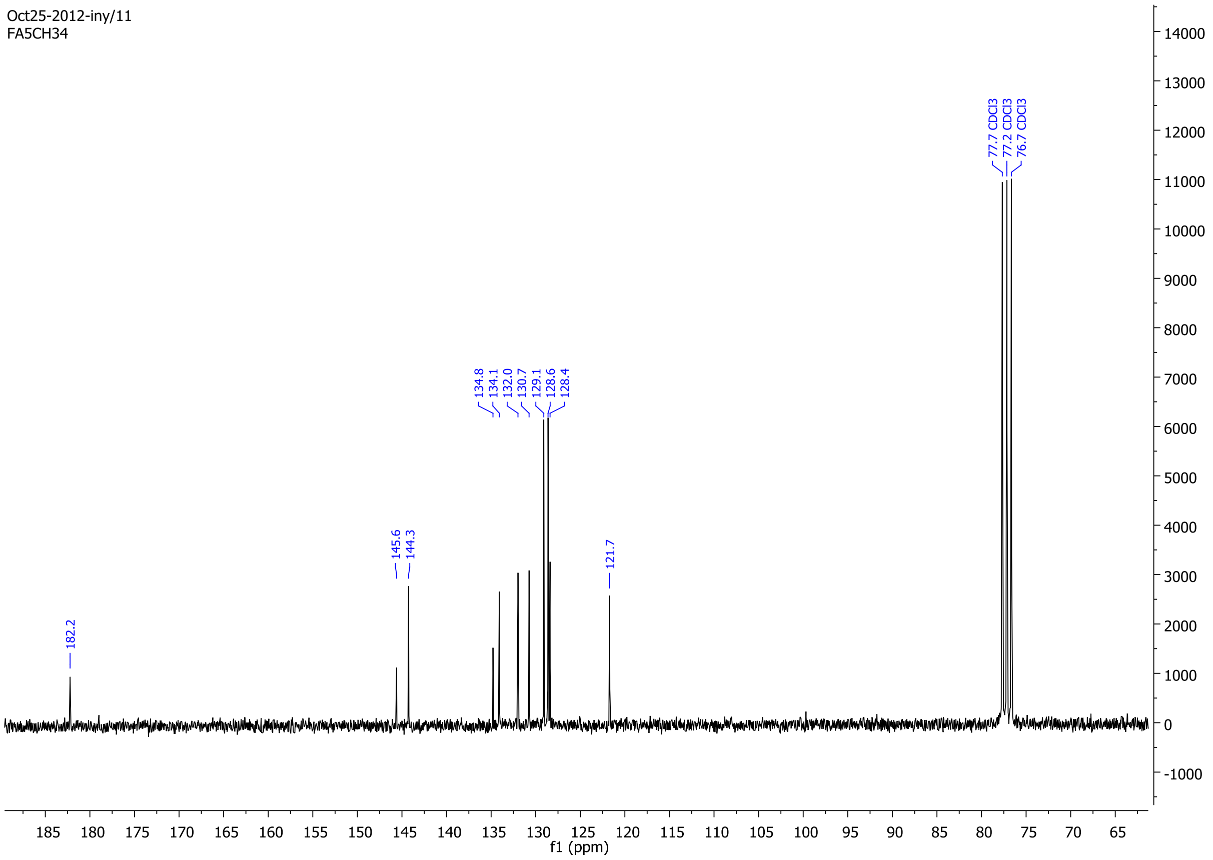

Figure S40. (*E*)-1-phenyl-3-(thiophen-2-yl)-2-propen-1-one **3ao**.

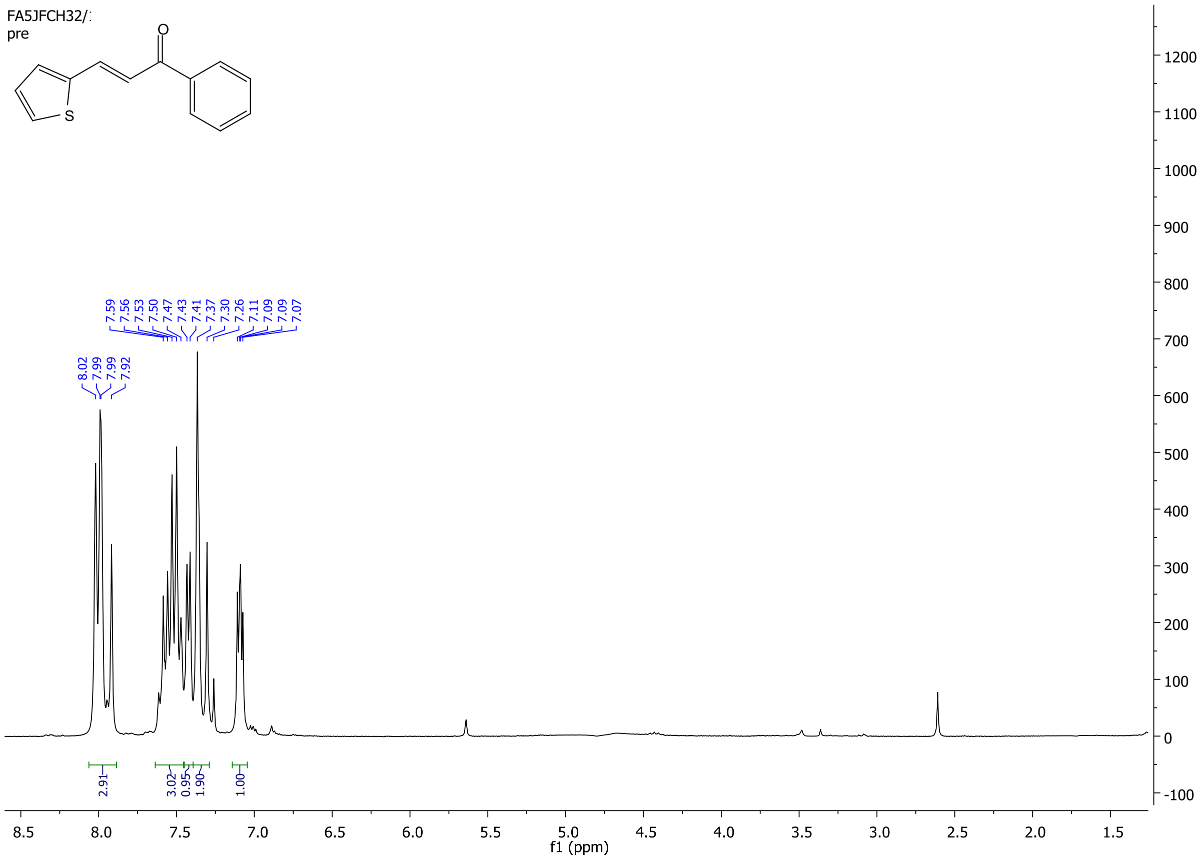

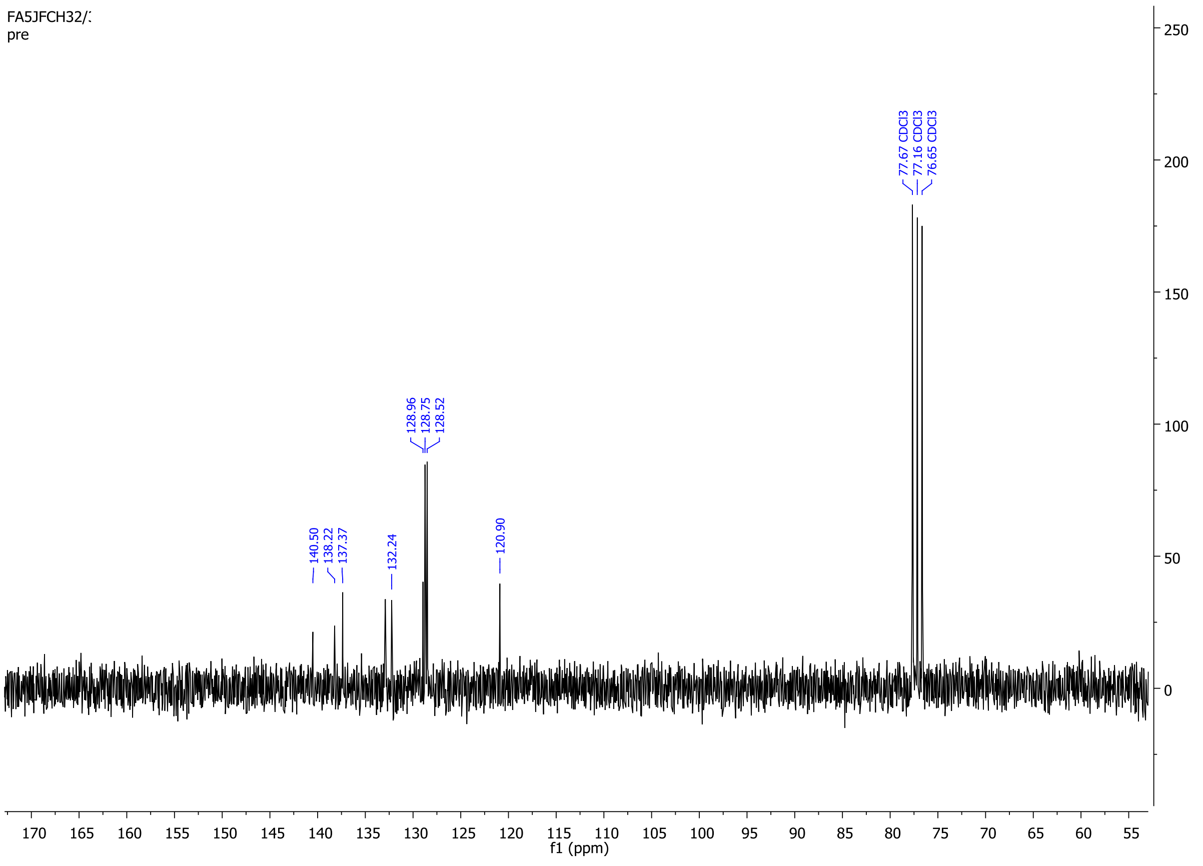

Figure S41. (*E*)-1,3-di(thiophen-2-yl)-2-propen-1-one **3ap**.

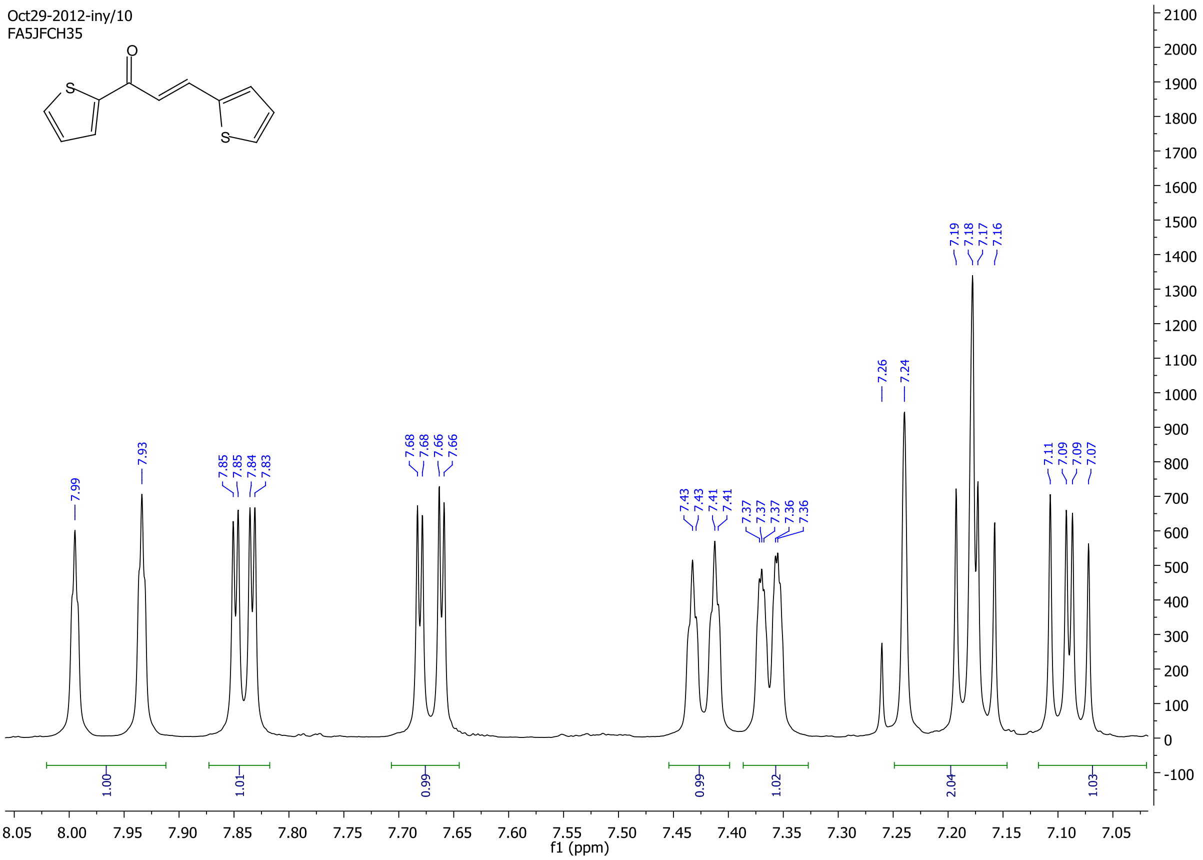

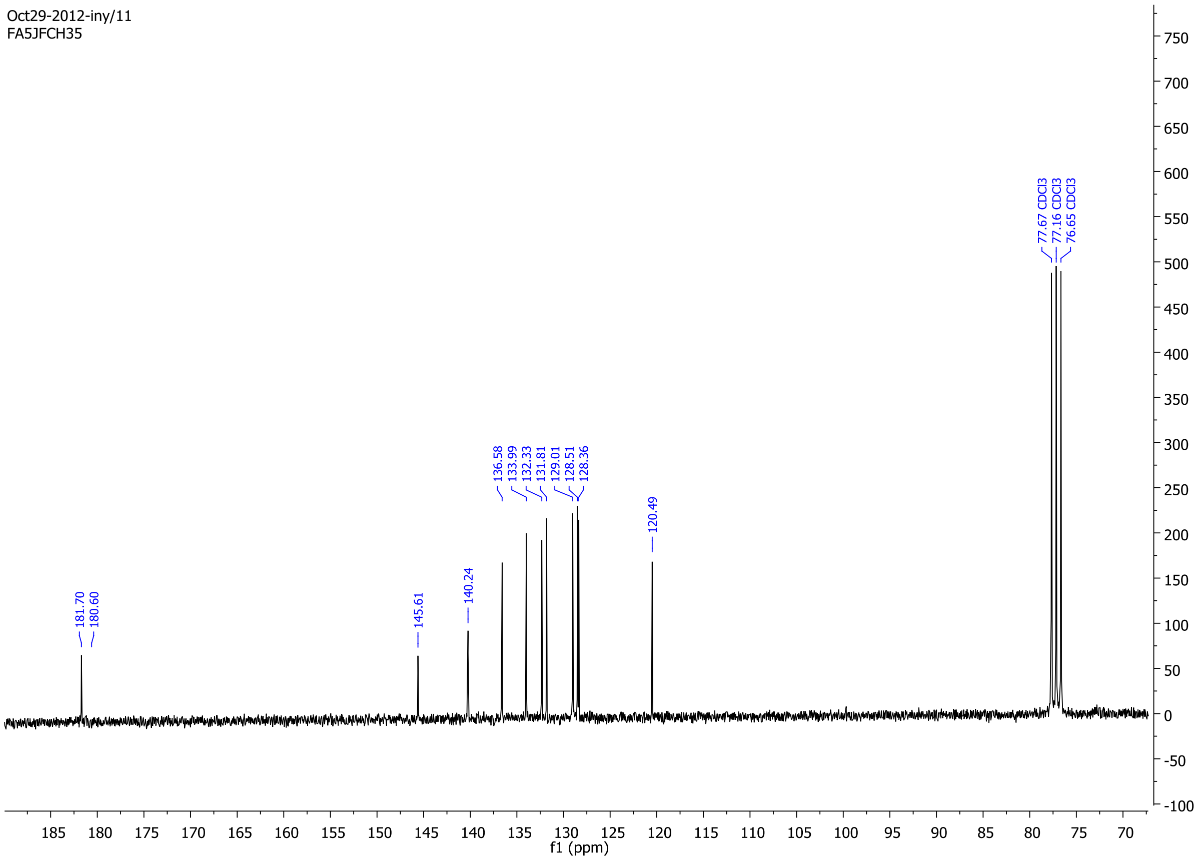

Figure S42. (2*E*,6*E*)-2,6-Dibenzylidenecyclohexanone **5**.

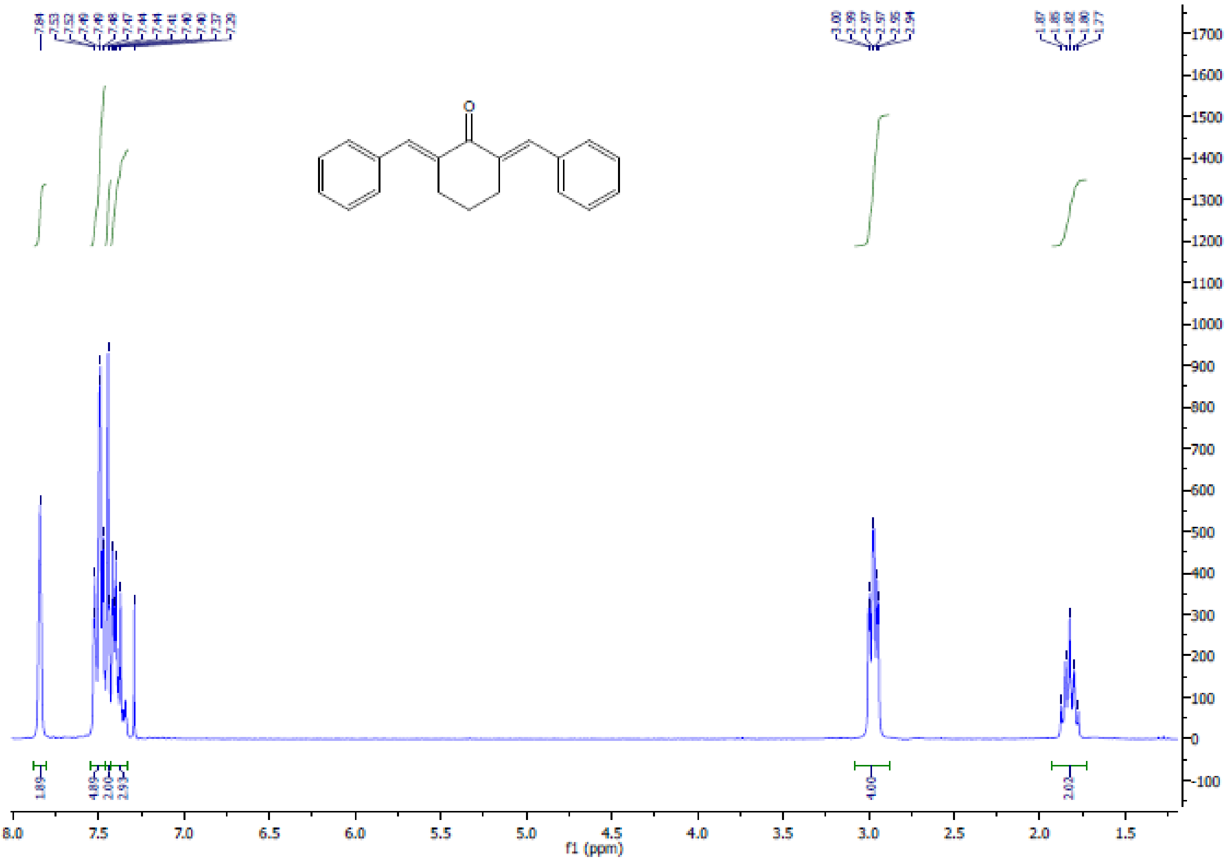

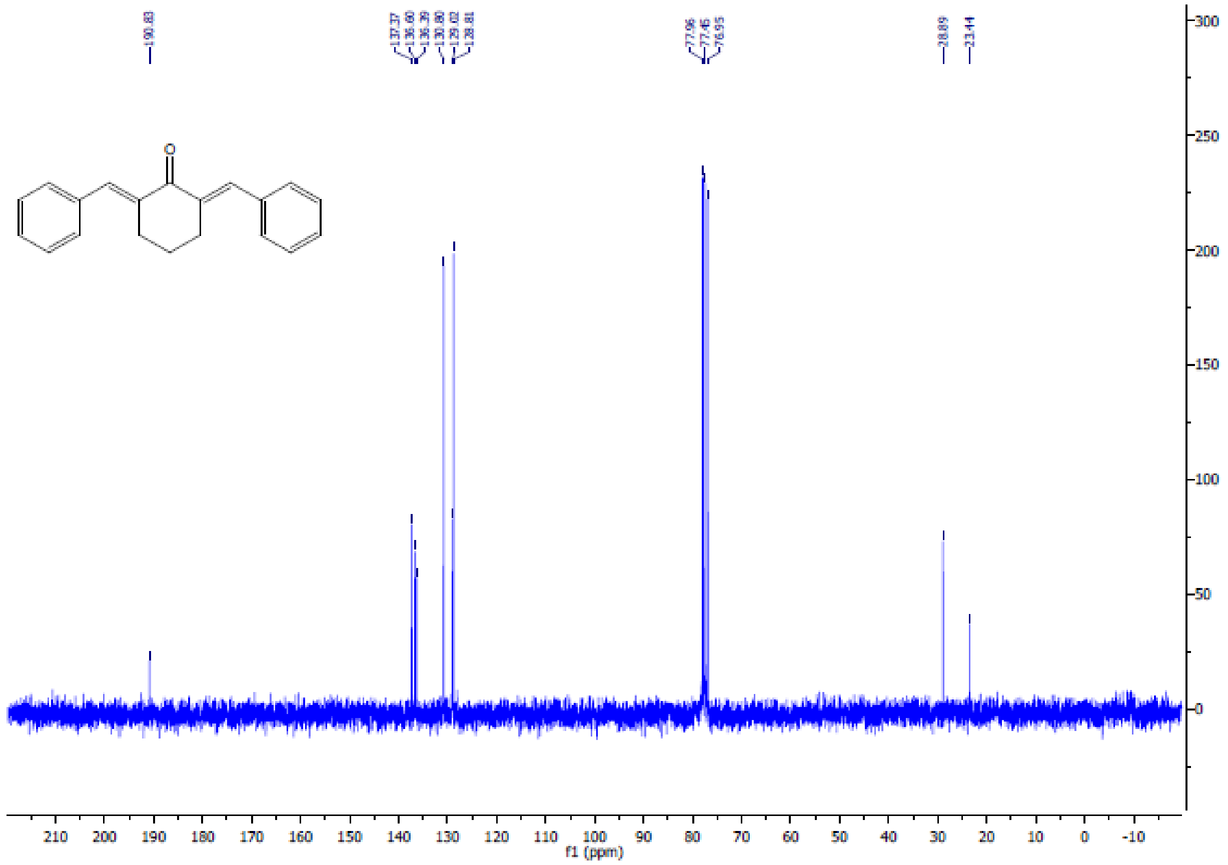

Figure S43. (1*E*,3*E*)-1,3-Dibenzylidene-3,4-dihydronaphthalen-2(1*H*)-one **6**.

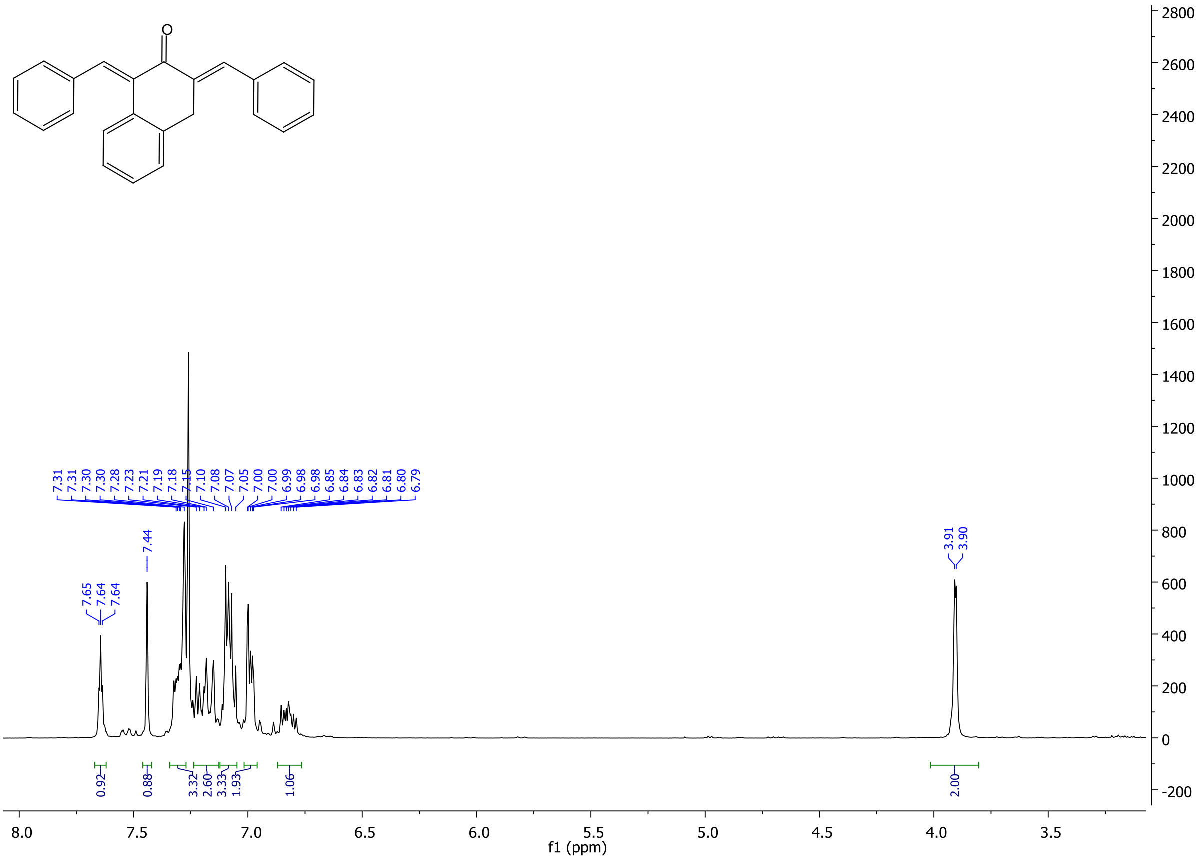

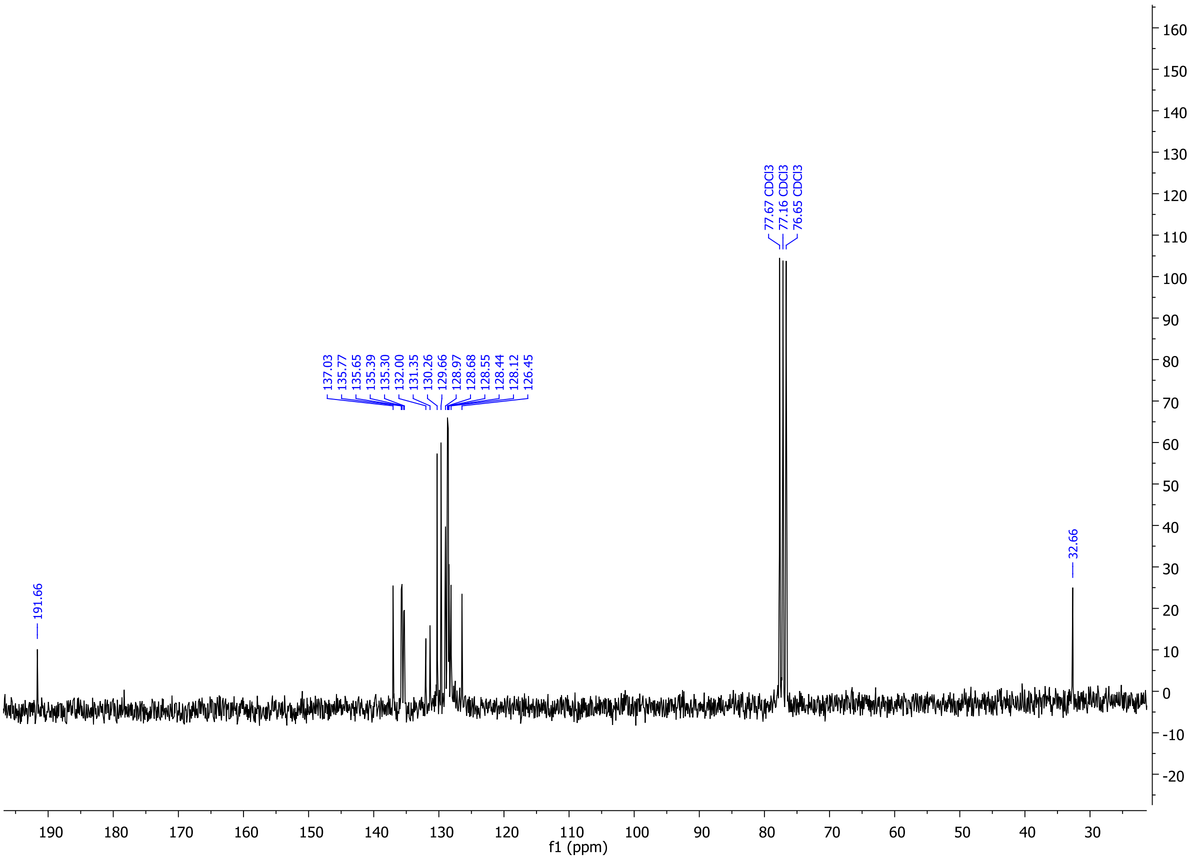

Figure S44. (3*E*,5*E*)-3,5-Dibenzylidene-1-methylpiperidin-4-one **7**.

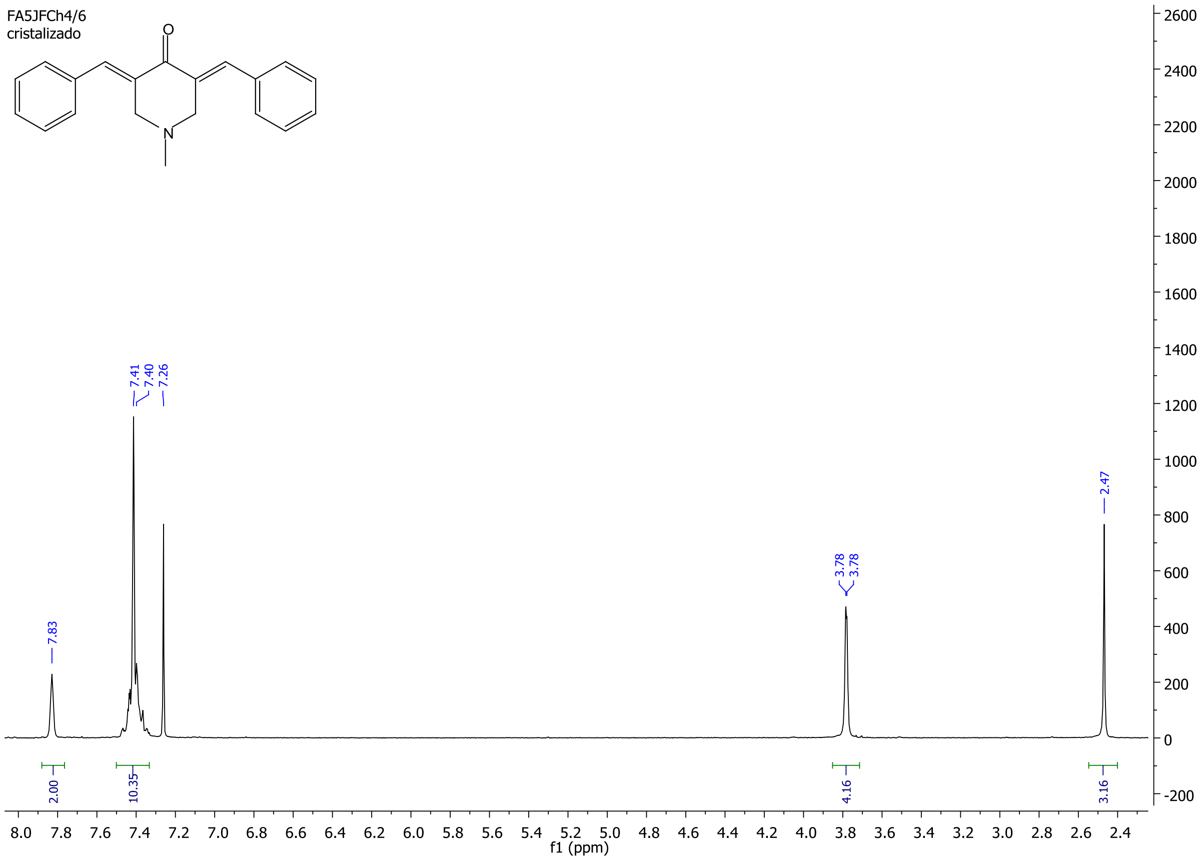

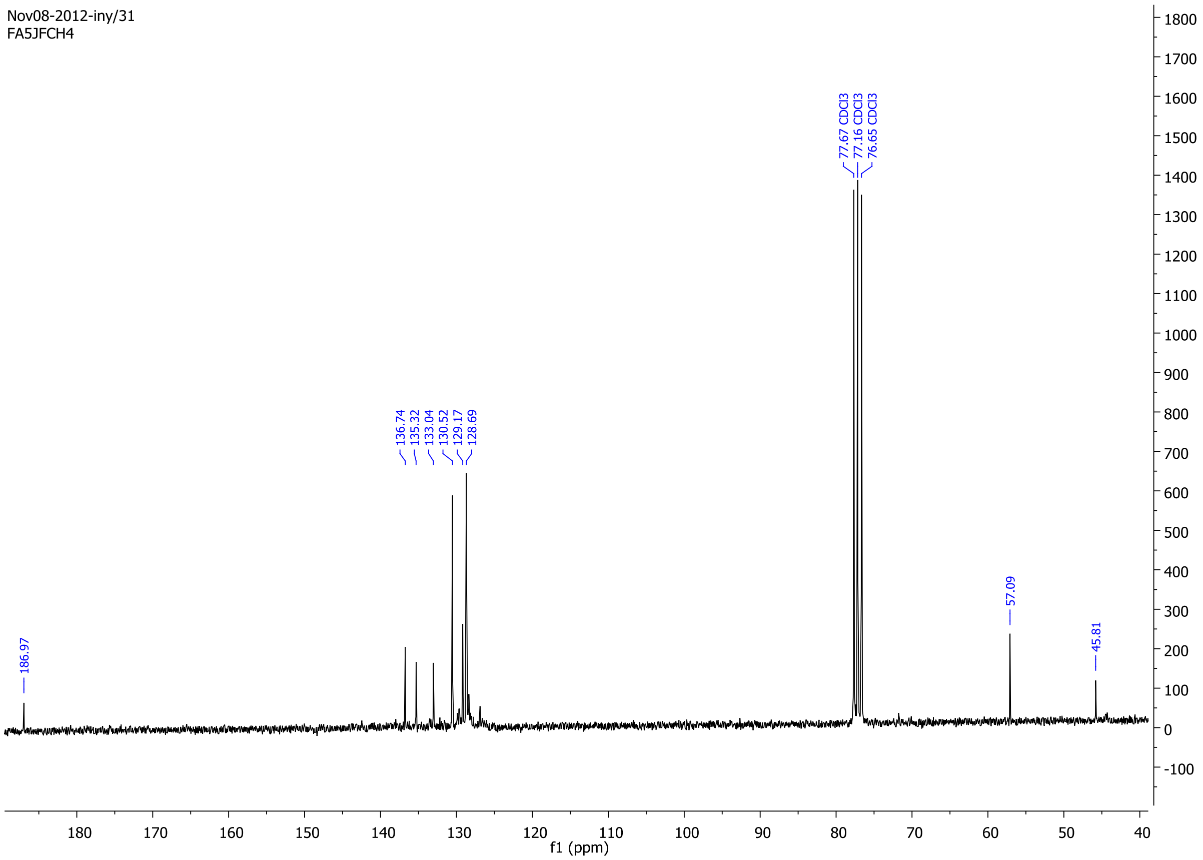

Figure S45. 3-benzylidenepentane-2,4-dione **8**.

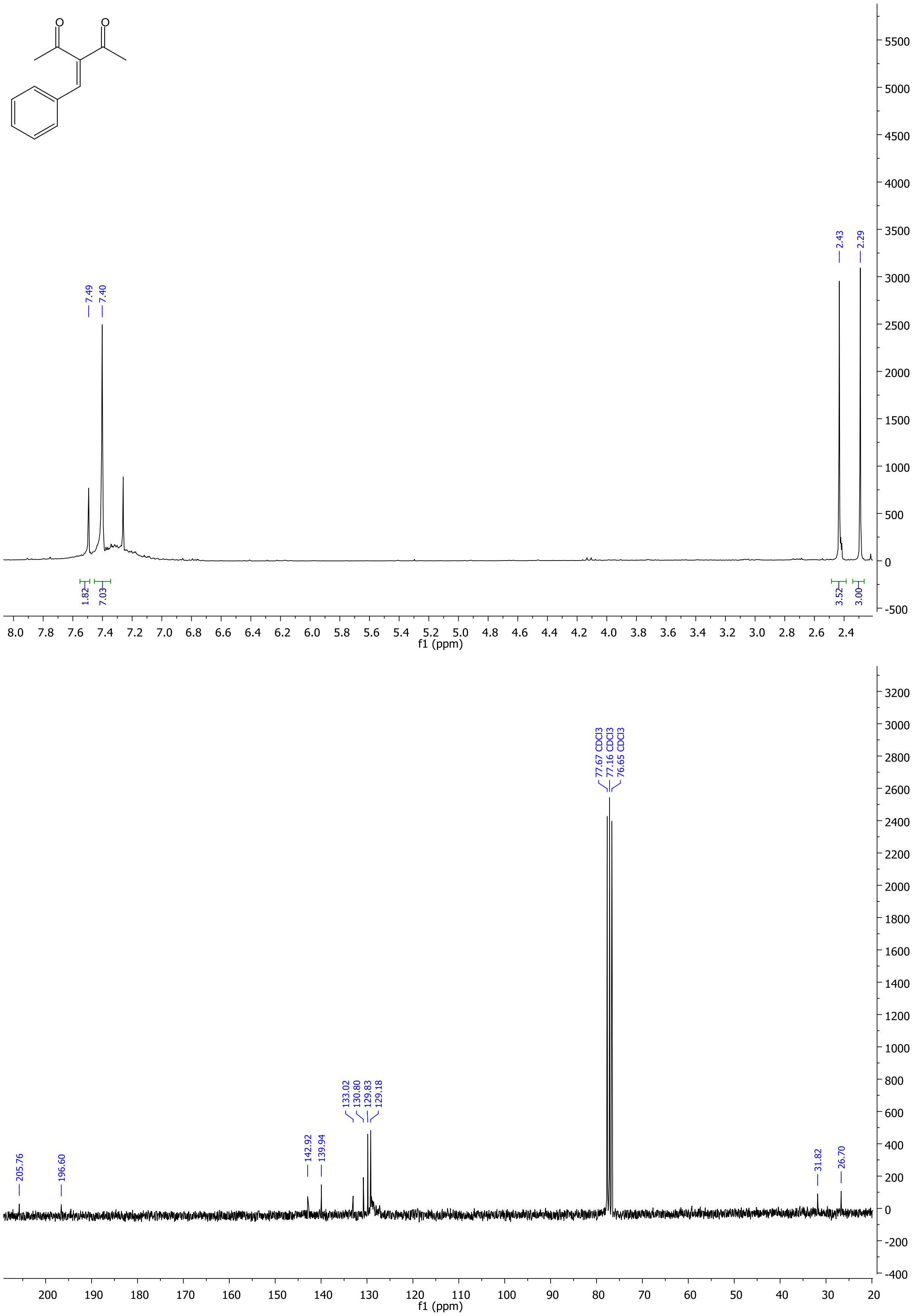

References

1. Urawa, Y.; Nishiura, K.; Souda, S.; Ogura, S. A convenient method for preparing aromatic α,β-unsaturated ketones from α,β-unsaturated acyl chlorides and arylboronic acids via Suzuki-Miyaura type coupling reaction. *Synthesis* **2003**, 2882–2885.
2. Cabrera, M.; Simoens, M.; Falchi, G.; Lavaggi, M.L.; Piro, O.E.; Castellano, E.E.; Vidal, A.; Azqueta, A.; Monge, A.; López de Ceráin, A.; *et al.* Synthetic chalcones, flavanones, and flavones as antitumoral agents: Biological evaluation and structure–activity relationships. *Bioorg. Med. Chem.* **2007**, *15*, 3356–3367.
3. Schramm, O.G.; Müller, T.J.J. Microwave-accelerated coupling-isomerization reaction (MACIR)—A general coupling-isomerization synthesis of 1,3-diarylprop-2-en-1-ones *Adv. Synth. Catal.* **2006**, *348*, 2565–2570.
4. Hayat, F.; Salahuddin, A.; Umar, S.; Azam, A. Synthesis, characterization, antiamoebic activity and cytotoxicity of novel series of pyrazoline derivatives bearing quinoline tail. *Eur. J. Med. Chem.* **2010**, *45*, 4669–4675.
5. Hu, Z.G.; Liu, J.; Dong, Z.B.; Gu, L.L.; Wang, D.; Zeng, P.L. Synthesis of chalcones catalysed by $SOCl_2$/EtOH. *J. Chem. Res.* **2004**, *2*, 158–159.
6. Huang, X.; Xie, L.; Wu, L.H. Synthetic applications of organotellurium compounds. 1. A facile synthesis of α,β-unsaturated esters, ketones, and nitriles. *J. Org. Chem*. **1988**, *53*, 4862–4864.
7. Kancyl, S.; David, B.J. Substituent effects on the carbonyl stretching frequency of chalcones. *J. Org. Chem*. **1970**, *35*, 759–764.
8. Powers, D.G.; Casebier, D.S.; Fokas, D.; Ryan, W.J.; Troth, J.R.; Coffen, D.L. Automated parallel synthesis of chalcone-based screening libraries. *Tetrahedron* **1998**, *54*, 4085–4096.
9. Kumar, A.; Akanksha. Zirconium chloride catalyzed efficient synthesis of 1,3-diaryl-2-propenones in solvent free conditions via aldol condensation. *J. Mol. Catal. A: Chem.* **2007**, *274*, 212–216.
10. Ashtekar, K.D.; Staples, R.J.; Borhan, B. Development of a formal catalytic asymmetric [4+2] addition of ethyl-2,3-butadienoate with acyclic enones. *Org. Lett*. **2011**, *13*, 5732–5735.
11. Ishikawa, T.; Mizuta, T.; Hagiwara, K.; Aikawa, T.; Kudo, T.; Saito, S. Catalytic alkynylation of ketones and aldehydes using quaternary ammonium hydroxide base. *J. Org. Chem.* **2003**, *68*, 3702–3705.
12. Fuson, R.C.; Jackson, H.L. Conjugate addition of mesitylmagnesium bromide to ethyl 2,4,6-trimethylcinnamate and to mesitalacetomesitylene. *J. Am. Chem. Soc.* **1950**, *72*, 1637–1638.
13. Sashidhara, K.V.; Rosaiah, J.N.; Kumar, A. Iodine-catalyzed mild and efficient method for the synthesis of chalcones. *Synth. Commun.* **2009**, *39*, 2288–2296.
14. Yang, J.-X.; Tao, X.-T.; Yuan, C.X.; Yan, Y.X.; Wang, L.; Liu, Z.; Ren, Y.; Jiang, M.H. A facile synthesis and properties of multicarbazole molecules containing multiple vinylene bridges. *J. Am. Chem. Soc.* **2005**, *127*, 3278–3279.
15. Rao, M.L.N.; Venkatesh, V.; Jadhav, D.N. A palladium catalyzed atom-efficient cross-coupling reactivity of triarylbismuths with α,β-unsaturated acyl chlorides. *J. Organomet. Chem.* **2008**, *693*, 2494–2498.

16. Zhou, J.; Wu, G.; Zhang, M.; Jie, X.; Su, W. Pd-Catalyzed cross-coupling of aryl carboxylic acids with propiophenones through a combination of decarboxylation and dehydrogenation. *Chem. Eur. J.* **2012**, *18*, 8032–8036.
17. Dannhardt, G.; Kiefer, W.; Kramer, G.; Maehrlein, S.; Nowe, U.; Fiebich, B. The pyrrole moiety as a template for COX-1/COX-2 inhibitors. *Eur. J. Med. Chem.* **2000**, *35*, 499–510.
18. Hayamizu, K.; Yanagisawa, M.; Ishii, T.; Yabe, A.; Yamamoto, O.; Nakayama, M.; Hayashi, A. ^{1}H and ^{13}C NMR spectra of 4,4′-substituted chalcones. *Magn. Res. Chem.* **1989**, *27*, 899–900.
19. Ono, M.; Haratake, M.; Morib, H.; Nakayama, M. Novel chalcones as probes for in vivo imaging of β-amyloid plaques in Alzheimer's brains. *Bioorg. Med. Chem.* **2007**, *15*, 6802–6809.
20. Buszek, K.R.; Brown, N. N-Vinylpyridinium and -ammonium tetrafluoroborate salts: New electrophilic coupling partners for Pd(0)-catalyzed Suzuki cross-coupling reactions. *Org. Lett.* **2007**, *9*, 707–710.
21. Tiwari, B.; Pratapwar, A.S.; Tapas, A.R.; Butle, S.R.; Vatkar, B.S. Synthesis and antimicrobial activity of some chalcone derivatives. *Int. J. Chem. Tech. Res*. **2010**, *2*, 499–503.
22. Wei, W.; Qunrong, W.; Liqin, D.; Aiqing, Z.; Duoyuan, W. Synthesis of dinitrochalcones by using ultrasonic irradiation in the presence of potassium carbonate. *Ultrason. Sonochem.* **2005**, *12*, 411–414.
23. Colotta, V.; Catarzi, D.; Varano, F.; Filacchioni, G.; Cecchi. L. Synthesis and binding activity of some pyrazolo[1,5-*c*]quinazolines as tools to verify an optional binding site of a benzodiazepine receptor ligand. *J. Med. Chem.* **1996**, *39*, 2915–2921.
24. Malia, R.S.; Tilve, S.G.; Desai, V.G. Synthesis of 2-acyl- and 2-benzoylindoles. *J. Chem. Res.* **2000**, *2000*, 8–9.
25. Cetin, A.; Cansiz, A.; Digrak, M. 3-Aryl-5-furylpyrazolines and their biological activities. *Heteroat. Chem*. **2003**, *14*, 345–347.
26. Arbuzova, S.N.; Glotova, T.E.; Dvorko, M.Y.; Ushakov, I.A.; Gusarova, N.K.; Trofimov, B.A. Stereoselective reduction of 1-acyl-2-phenylacetylenes with triphenylphosphine in water: An efficient synthesis of *E*-chalcones. *ARKIVOC* **2011**, *xi*, 183–188.
27. Liu, W.H.-M. Shi, H. Jin, H.-Y, Zhao, G.-P. Zhou, F. Wen, Z.-Y. Yu, T.-P. Hou. Design, synthesis and antifungal activity of a series of novel analogs based on diphenyl ketones. *Chem. Biol. Drug Des.* **2009**, *73*, 661–667.
28. Emerson, W.S.; Patrick, T.M. The preparation of 2-thiophenealdehyde and some of its derivatives. *J. Org. Chem.* **1949**, *14*, 790–797.
29. Shibata, K.; Katsuyama, I.; Matsui, M.; Muramatsu, H. Synthesis of 3-cyano-2-methylpyridines substituted with heteroaromatics. *J. Heterocycl. Chem*. **1991**, *28*, 161–165.
30. Ko, H.-H.; Tsao, L.-T.; Yu, K.-L.; Liu, C.-T.; Wang, J.-P.; Lin, C.-N. Structure–activity relationship studies on chalcone derivatives: The potent inhibition of chemical mediators release. *Bioorg. Med. Chem*. **2003**, *11*, 105–111.
31. Wang, L.; Sheng, J.; Tian, H.; Han, J.; Fan, Z.; Changtao, Q. A convenient synthesis of α,α′-bis(substituted benzylidene)cycloalkanones catalyzed by $Yb(OTf)_3$ under solvent-free conditions. *Synthesis* **2004**, 3060–3064.

32. Dimmock, J.R.; Padmanilyam, M.P.; Zello, G.A.; Quail, J.W.; Oloo, E.O.; Prisciak, J.S.; Kraatz, H.-B.; Cherkasov, A.; Lee, J.S.; Allen, T.M.; *et al.* Cytotoxic 1,3-diarylidene-2-tetralones and related compounds. *Eur. J. Med. Chem.* **2002**, *37*, 813–824.
33. Pati, H.N.; Das, U.; Das, S.; Bandy, B.; de Clercq, E.; Balzarini, J.; Kawase, M.; Sakagami, H.; Quail, J.W.; Stables, J.P.; *et al*. The cytotoxic properties and preferential toxicity to tumour cells displayed by some 2,4-bis(benzylidene)-8-methyl-8-azabicyclo[3.2.1]octan-3-ones and 3,5-bis(benzylidene)-1-methyl-4-piperidones. *Eur. J. Med. Chem.* **2009**, *44*, 54–62.
34. Yi, W.-B.; Cai, C. Perfluoroalkylated pyridine catalyzed Knoevenagel condensation: An important complement of fluorous catalysis without fluorous solvent. *Catal. Commun*. **2008**, *9*, 1291–1296.

© 2014 by the authors; licensee MDPI, Basel, Switzerland. This article is an open access article distributed under the terms and conditions of the Creative Commons Attribution license (http://creativecommons.org/licenses/by/3.0/).
